# Hepatitis B/C in the countries of the EU/EEA: a systematic review of the prevalence among at-risk groups

**DOI:** 10.1186/s12879-018-2988-x

**Published:** 2018-02-12

**Authors:** Abby May Falla, Sanne Henrietta Ina Hofstraat, Erika Duffell, Susan Josien Maria Hahné, Lara Tavoschi, Irene Karen Veldhuijzen

**Affiliations:** 1 Division of Infectious Disease Control, Municipal Public Health Service Rotterdam-Rijnmond, Rotterdam, the Netherlands; 2 Department of Public Health, Erasmus MC, University Medical Centre Rotterdam, Box 70032, 3000 LP Rotterdam, The Netherlands; 3 National Institute for Public Health and the Environment (RIVM), Centre for Infectious Disease Control, Postbus 1, 3720 BA Bilthoven, the Netherlands; 4 European Centre for Disease Prevention and Control, Granits väg 8, 171 65 Solna, Sweden

**Keywords:** Hepatitis B, Hepatitis C, Prevalence, Men who have sex with men, People who inject drugs, Prisoners, Higher risk groups, Systematic review [publication type]

## Abstract

**Background:**

In 2016, the World Health Organisation set a goal to eliminate viral hepatitis by 2030. Robust epidemiological information underpins all efforts to achieve elimination and this systematic review provides estimates of HBsAg and anti-HCV prevalence in the European Union/European Economic Area (EU/EEA) among three at-risk populations: people in prison, men who have sex with men (MSM), and people who inject drugs (PWID).

**Methods:**

Estimates of the prevalence among the three risk groups included in our study were derived from multiple sources. A systematic search of literature published during 2005–2015 was conducted without linguistic restrictions to identify studies among people in prison and HIV negative/HIV sero-status unknown MSM. National surveillance focal points were contacted to validate the search results. Studies were assessed for risk of bias and high quality estimates were pooled at country level. PWID data were extracted from the European Monitoring Centre for Drugs and Drug Addiction (EMCDDA) repository.

**Results:**

Despite gaps, we report 68 single study/pooled HBsAg/anti-HCV prevalence estimates covering 23/31 EU/EEA countries, 42 of which were of intermediate/high prevalence using the WHO endemicity threshold (of ≥2%). This includes 20 of the 23 estimates among PWID, 20 of the 28 high quality estimates among people in prison, and four of the 17 estimates among MSM. In general terms, the highest HBsAg prevalence was found among people in prison (range of 0.3% - 25.2%) followed by PWID (0.5% - 6.1%) and MSM (0.0% - 1.4%). The highest prevalence of anti-HCV was also found among people in prison (4.3% - 86.3%) and PWID (13.8% - 84.3%) followed by MSM (0.0% - 4.7%).

**Conclusions:**

Our results suggest prioritisation of PWID and the prison population as the key populations for HBV/HCV screening and treatment given their dynamic interaction and high prevalence. The findings of this study do not seem to strongly support the continued classification of MSM as a high risk group for chronic hepatitis B infection. However, we still consider MSM a key population for targeted action given the emerging evidence of viral hepatitis transmission within this risk group together with the complex interaction of HBV/HCV and HIV.

**Electronic supplementary material:**

The online version of this article (10.1186/s12879-018-2988-x) contains supplementary material, which is available to authorized users.

## Background

Chronic infection with the hepatitis B (HBV) or hepatitis C virus (HCV) is a significant cause of liver disease-related morbidity and mortality in the European Union/European Economic Area (EU/EEA) [1]. Both viruses are transmitted through contact with infected blood, blood products and other bodily fluids. HBV is vaccine preventable which, along with other primary prevention measures including health care infection control and antenatal screening, have led to a decrease in acute and chronic hepatitis B (CHB) incidence in many EU/EEA countries [2]. Health care infection control together with harm reduction programmes among people who inject drugs (PWID) have also led to some decrease in the HCV incidence in many countries [3]. Many EU/EEA countries now face a dichotomy: a declining incidence of new HBV/HCV infections in the general population due to the success of primary prevention [2, 4] alongside a projected increase in liver disease-related morbidity and mortality due to ageing of the chronically infected population [5, 6]. With the availability of antiviral treatment that can effectively halt disease progression in CHB, including progression to cirrhosis and hepatocellular carcinoma, and new direct acting antivirals for chronic hepatitis C (CHC) that report cure rates in more than 90% of cases [7, 8], elimination of chronic viral hepatitis is a possibility. Elimination requires expanded access to screening, efficient linkage to care and retention in treatment among risk populations. Timely, reliable prevalence data are needed to understand which populations are most affected to better target screening and treatment programmes, and to monitor the performance and impact of these activities at a strategic level. Indeed, for screening to have a more favourable cost-effectiveness ratio and lead to an overall net gain in population health, current evidence indicates that it should be targeted to higher prevalence populations including PWID and other risk populations, where the expected case yield would be highest. [9, 10] However, the prevalence threshold above which a favourable cost effectiveness ratio varies considerably between EU/EEA countries.

In terms of key at-risk populations, men who have sex with men (MSM) are considered a high risk population for viral hepatitis due to the efficacy of sexual contact in transmitting HBV and the high prevalence of other sexually transmitted infections especially Human Immuno-Deficiency Virus (HIV). Whilst sexual contact was historically considered an ineffective route of HCV transmission, an increased HCV incidence among MSM who have not/do not inject drugs has been reported since the early 2000s. There is increasing evidence of permucosal transmission of HCV, especially among HIV positive MSM, although sexually acquired HCV infection remains rare in HIV negative, non-injecting MSM. [11, 12] Hahné et al. reported Hepatitis B surface Antigen (HBsAg) and anti-HCV (measures of evidence of chronic HBV and chronic or resolved HCV infection respectively) prevalence among MSM in the EU/EEA ranging from < 1% to 4% and from > 1% to 2.9%, respectively [13].

People detained in prison settings are considered a high risk population for blood-borne virus infection due the criminalisation of high transmission risk behaviour such as injecting drug use and sex work, coupled with pre-detention social vulnerability (such as experience of domestic abuse, poverty and homelessness) among many people detained and convicted. Prison-acquired blood-borne virus infections may also occur due to the continuation of transmission risk behaviour, the limited availability of harm reduction services and the lack of adequate infection control practices [14, 15]. Dolan et al. meta-analysed data in Global Burden of Disease regions: in Western Europe, HBsAg and anti-HCV prevalence among people in prison was reported to be 2.4% and 15.5%, respectively, while in Eastern Europe it was 10.4% for HBsAg and 20.2% for anti-HCV [16]. HBsAg and anti-HCV estimates are also available for nine and 13 EU/EEA countries, respectively, although no study quality assessment nor country-level meta-analysis/pooling were performed.

Of the three at-risk populations included in this study, PWID are considered at highest risk due to the efficacy of unsafe injecting behaviour in transmitting HBV and HCV. This together with clustering of social and environmental risk factors in this marginalised population such as a history of incarceration, poverty, homelessness and multi-morbidity compound their vulnerability [17]. Nelson et al. conducted a global review of HBsAg and anti-HCV prevalence among PWID in 2010, and reported prevalence data for 30 EU/EEA countries for anti-HCV and for 26 EU/EEA countries for HBsAg. The prevalence of anti-HCV ranged from 21.1% in Finland to 90.5% in Latvia, whereas HBsAg prevalence ranged from 0.0% in Ireland and Cyprus to 21.3% in Estonia [17]. Wiessing et al. performed a systematic review of various epidemiological measures of the HCV epidemic among PWID in Europe [18]. Although anti-HCV prevalence was not an included outcome, their findings across the cascade of care show that 72% of anti-HCV infected PWID are viraemic; that 49% are unaware of their infection; and that 9.5% of diagnosed cases are reported to be on treatment. A review focused on the EU/EEA in 2009, Hahné et al. reported HBsAg prevalence among PWID to be between 0.0% and 21.3% and anti-HCV prevalence to be between 5.3% and 90% [13]. An updated synthesis of the prevalence in this priority population is required.

Our study is part of a larger project funded by the European Centre for Disease Prevention and Control (ECDC) that seeks to provide a timely update on available estimates across a number of low risk populations (the general population, pregnant women and first-time blood donors) and as a comparator/contrast to collate prevalence estimates in high risk populations. We describe the results of the study into chronic viral hepatitis low risk populations and among migrants elsewhere [19, 20]. In the study reported here, we seek to update and expand the work of the previous ECDC systematic review (from 2009) by Hahné et al. [21] of prevalence estimates for markers of hepatitis B (HBsAg) and C (anti-HCV) in three key risk groups: MSM, people who inject drugs and people incarcerated in prison. Our study seeks to contribute to the elimination of viral hepatitis in Europe by providing information to support the design and management of primary and secondary prevention strategies.

## Methods

### Data sources

Estimates of the prevalence among the three risk groups included in our study were derived from multiple sources. A systematic literature search was conducted according to PRISMA guidelines [22] to retrieve, assess and synthesize available data published in the period 2005–2015 on the prevalence of HBsAg and anti-HCV infection in MSM and people in prison. Data on the prevalence among PWID were retrieved from the European Monitoring Centre for Drugs and Drug Addiction (EMCDDA) [23, 24].

### Definitions

The key outcome was prevalence, which was defined as the proportion of study subjects with a positive finding of HBsAg or anti-HCV in serum, saliva or dry blood spot samples. MSM were not formally defined beyond the search term/inclusion criterion of ‘MSM’ and in practice this conceptualisation included men participating in studies in MSM-specific venues (e.g. saunas). People in prison were defined as people incarcerated in prison settings including prisons, remand centres, youth detention centres and psychiatric prison hospitals but excluding formerly incarcerated populations and other non-custodial secure institutions (such as secure psychiatric hospitals). PWID were defined by the EMCDDA as any person who has ever in their lifetime injected a drug for non-medical purposes [25].

### The prevalence in MSM and people in prison: Systematic review

#### Search strategy

A systematic search to retrieve original research articles was conducted in PubMed®, Embase® and Cochrane Library bibliographic databases in March 2015. The search strategy (described in the Additional file 1) combined controlled (i.e. MeSH/Emtree terms) and natural vocabulary (i.e. keywords) to define disease-related (HBV or HCV infection), outcome-related (prevalence), and geographic-related search parameters (EU/EEA). To maximise the yield of the search, no population-specific search terms were included. Population relevancy was instead assessed at the title/abstract and full text assessment stages, as described below. The search was limited to records published from 1 January 2005 to 12 March 2015. Articles in all EU/EEA languages were included. The results of the search were shared with ECDC National Focal Points [26] for viral hepatitis in all EU/EEA Member States in May 2015 to review and validate the list of included references for their country. The data extraction, risk of bias assessment and data analysis described below were all performed in Microsoft Excel.

### Inclusion/exclusion criteria

**Fig. 1:**
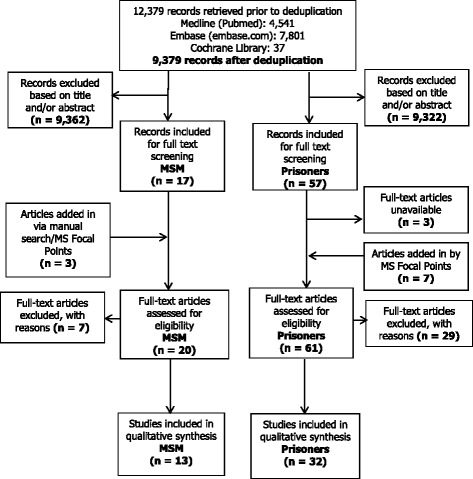
PRISMA flowchart for studies retrieved among MSM and people in prison

Inclusion/exclusion criteria included publication and data collection date ranges, geographical relevancy, the reporting of specific markers of hepatitis B/C infection, population relevancy (as outlined in the definitions section above) and study design. Criteria related to study design included the actual measured presence of viral markers in bodily fluid/dried blood spot samples (and thus the exclusion of self-reported infection) in human subjects, prevalence as an outcome measure, the exclusion of modelled data only, and the exclusion of guidelines, meta-analyses, systematic reviews and commentary/opinion pieces. These criteria were twice piloted and refined by two reviewers (AF/SHIH) on a random sample of 5% of articles in order to reach > 95% concordance. Following this, the title/abstract screening continued separately using Endnote. The full text of all publications included during title/abstract screening were individually assessed for relevance by members of the research team (for articles in Dutch, English, French, German and Italian) or by ECDC reviewers (for any other EU/EEA languages). Reviewers consulted each other in cases of uncertainty about in- or exclusion, and with a third team member (IV) to resolve further disagreement. The full search strategy, the in- and exclusion criteria and the PRISMA checklist are available in the Additional file 1 accompanying this article. See Fig. 1 for the PRISMA flowchart (reasons for full text exclusion are detailed in the Additional file 1).

#### Data extraction

Data extraction using a pre-defined set of variables was performed simultaneously with full text screening. The unit for data extraction was study, not article. A study was defined as the report of prevalence data on HBsAg or anti-HCV for a defined population group, in a defined country, over a discrete period of time and one article may therefore include more than one study. Studies published in more than one article were extracted only once and the article with the most detail about the study used as reference. For studies retrieved reporting the prevalence in MSM, only data on HIV negative or unknown/unmeasured HIV sero-status MSM were extracted. All results reported in the study relating to MSM are therefore among HIV negative or unknown HIV sero-status MSM.

#### Risk of bias assessment

For MSM and the prison population each study was evaluated for the risk of selection bias using a specifically developed assessment framework. To account for differences in sources of selection bias, separate assessment frameworks were developed for MSM and people in prison to determine the representativeness of sample for that specific target population and the robustness of the estimates in each study. For studies in MSM, just one domain was included, ‘sampling venue coverage’, where the risk of bias was considered smaller for studies in multiple venues or multiple venue types. For studies in the prison population, the domains of age, gender, proportion of PWID, sampling method and geographical coverage were considered as possible sources of selection bias. Points were awarded in each domain for representativeness or a lower risk of bias, and a total score calculated by summing the values in each domain. This resulted in a score of between zero and two for MSM and between zero and six for the prison population. We refer to the total score as study quality score, since a higher score indicates a lower risk of bias.

#### Data analysis

We recalculated 95% confidence intervals (CIs) for all crude and pooled estimates using the Fisher’s Exact method. All prevalence estimates retrieved for MSM, irrespective of the study quality score, are presented in separate (one for hepatitis B and one for hepatitis C) forest plots prepared using Microsoft Excel. HBsAg and anti-HCV prevalence estimates obtained from studies among adults in prison with a high study quality score (≥3) were pooled, when possible, by summing cases and sample size. Pooled or single study-derived high quality estimates retrieved for people in prison are presented in a forest plot, unless a study reported data over time whereby the most recent estimate was selected. Adult and juvenile (as defined by the included studies) estimates for the prison population are shown separately.

### The prevalence in PWID: Extraction from the EMCDDA data repository

The EMCDDA systematically retrieves, synthesizes and publishes comprehensive (and often unpublished) data on the prevalence of viral infections among PWID [23, 24]. We opted to draw on this repository due to its extensive scope as well as the potential to retrieve unpublished data that would be unavailable in scientific literature. The full data set retrieved for use in this study included country, year of study, geographical coverage, sample size and prevalence as well as limited information about study design and recruitment method/setting. We included only national level estimates, and where multiple national estimates were available for a country, the most recent estimate was selected. We did not assess the quality of the study beyond these parameters of geographical coverage and recency. Number positive was back calculated using prevalence and sample size, and a 95% CI re-calculated using the Fisher Exact method. Samples in fewer than 10 subjects were excluded and multiple national-level estimates (if available from a specific year for an EU/EEA country) were pooled by summing cases and sample size.

## Results

### Literature/database search retrievals

The literature search retrieved 9379 citations, from which 17 citations were included for MSM and 57 for people in prison based on title/abstract. Seventeen MS validated our search results or provided additional references. For people in prison, seven publications were added either through a manual search of retrieved studies or through the national viral hepatitis ECDC focal points. An additional three citations were added for MSM. Whilst all 20 full texts were retrieved for MSM, three of the 64 included for people in prison were unavailable. Following full text screening, 13 articles were included for MSM and 32 for people in prison. The database search of the EMCDDA data repository retrieved seven national level HBsAg and 16 national level anti-HCV prevalence estimates.

### The prevalence of HBsAg and anti-HCV among HIV negative/unknown HIV sero-status MSM

A total of 17 prevalence estimates, six for HBsAg and 11 for anti-HCV, were extracted from the 13 included studies about *HIV negative/unknown HIV sero-status* MSM. Key study details, including the risk of bias assessment, for all reported estimates among MSM are available in Annex 8 (HBV) and 9 (HCV) in the Additional file 1 for this article.

**Fig. 2:**
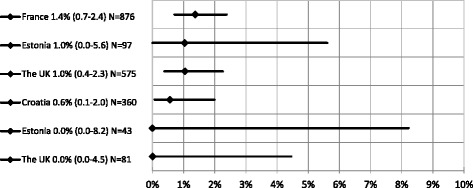
HBsAg Prevalence among *HIV negative/unknown HIV sero-status* MSM. Y axis - Country, prevalence estimate (95% CI) and sample size (N); X axis: HBsAg prevalence

The six HBsAg prevalence estimates covered four countries: one each from Croatia and France and two each from Estonia and the United Kingdom (UK). HBsAg prevalence ranged from 0.0% - 0.1% in Estonia [27, 28] and the UK to 1.4% in France (Fig. 2). The prevalence in the UK was derived from STI clinics in Scotland in 2001–2003 and ranged from 0.0% [29] to 1.0% [30], with the sample size of the study for the latter estimate considerably larger than the former study (*N* = 575 vs *N* = 81). The estimate from France is based on a large (*N* = 876), multi-centre, multi venue type study from 2009 [31].

**Fig. 3:**
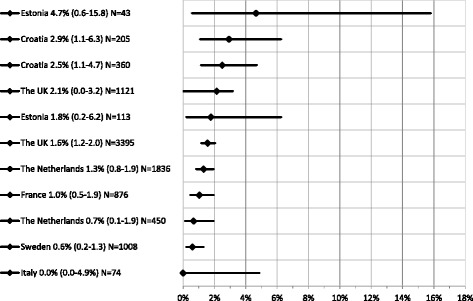
Anti-HCV Prevalence among *HIV negative/unknown HIV sero-status* MSM. Y axis - Country, prevalence estimate (95% CI) and sample size (N); X axis: Anti-HCV prevalence

The 11 anti-HCV estimates covered seven countries and the prevalence ranged from 0.0% in Italy [32] to 4.7% in Estonia (Fig. 3), with eight of the 11 data points ≥ 1%. Single estimates were available for France [31], Italy [32] and Sweden [33] whereas multiple estimates were available for Croatia, Estonia, the Netherlands and the UK. For Estonia, the two anti-HCV estimates range from 4.7% (in 2013) to 1.8% (in 2014–15). [27, 28] The two estimates for Croatia range from 2.5% [34] to 2.9% [35] and cover broadly the same time period (2003–2006) although the former sampled the population of Zagreb only while the latter covered seven cities. The two estimates from the Netherlands range from 0.7% to 1.3% and were derived using different study designs among the MSM population in Amsterdam; men attending a STI clinic opting out of HIV testing in 2007 [36] and a cohort study over the period 1984–2003, respectively [37]. The two estimates for the UK were: 2.2%, found in a multi-centre study between 2008/9 (*N* = 1121) in London gay bars, clubs and saunas; and 1.6% found among STI clinic attendees in Sheffield in 2009–2011 (*N* = 3395) [38, 39]. In summary, prevalence among MSM ranged from 0.0% to 1.4% for HBsAg and from 0.0% to 4.7% for anti-HCV.

### The prevalence of HBsAg and anti-HCV among people in prison

**Fig. 4:**
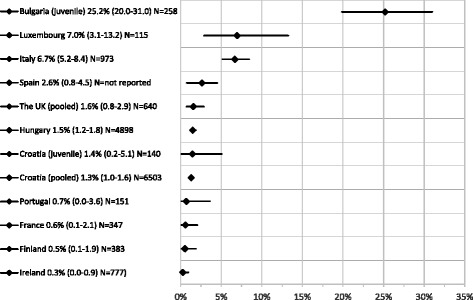
HBsAg prevalence among people (adults unless noted as juveniles) in prison. Y axis - Country, prevalence estimate (95% CI) and sample size (N); X axis: HBsAg prevalence

Fifteen HBsAg prevalence estimates for 12 countries were extracted from the 32 included articles, only one of which (in Romania) scored < 3 in the study quality assessment. Single estimates of HBsAg prevalence were retrieved for Bulgaria, Finland, France, Hungary, Ireland, Italy, Luxembourg, Portugal and Spain. Multiple (and therefore pooled) estimates were found for Croatia and the UK (Fig. 4). These data show considerable heterogeneity in HBsAg prevalence in the prison population in the EU/EEA from < 1% in the UK, Ireland [40], Finland [41] and France [42] to 6.7% in Italy [43], 7.0% in Luxembourg [44], 10.7% in Portugal [45] and 25.2% in Bulgaria [46]. Two estimates for Croatia obtained over 2004–2006 and 2005–2007 both report a HBsAg prevalence of 1.3% in adult inmates, with a third study from 2005 to 2007 reporting 1.4% among juvenile inmates. Two estimates obtained in the UK, one in a maximum security psychiatric hospital prison (reporting 0.0%) and the other in a general prison in London (reporting 2.0%), were pooled into an estimate of 1.6% (95% CI 0.8–2.9). Whilst diversity in sampling design was seen across the 14 high quality HBsAg prevalence estimates, just one study [44] was biased towards exclusive recruitment of PWID or use of injecting drug use as a sampling criterion. Key study details, including the risk of bias assessment, for all reported estimates among people in prison are available in Annex 10 (HBV) and 11 (HCV) in the Additional file 1 for this article.

**Fig. 5:**
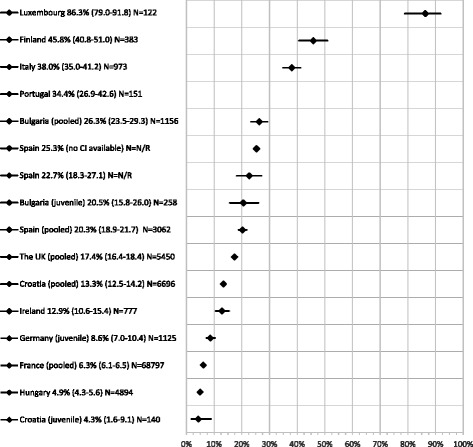
Anti-HCV prevalence among people (adults unless noted as juveniles) in prison. Y axis - Country, prevalence estimate (95% CI) and sample size (N) (N/R = not reported); X axis: Anti-HCV prevalence

Forty-five estimates of anti-HCV prevalence were retrieved from the included studies of which 37 estimates for 11 countries were considered high quality (i.e. a study quality score of ≥3). In 17 of the 45 anti-HCV estimates, injecting drug use was a study inclusion criterion or current/former PWID formed the majority of subjects. Figure 5 shows the final 16 single study/pooled high quality estimates included. There is considerable heterogeneity in the (mostly high i.e. ≥8%) prevalence among people in prison across the EU/EEA; all but four estimates (from Croatia (juvenile), France, Germany (juvenile) and Hungary) were above 10%, with an estimate from Luxembourg as high as > 80% prevalence [44]. Multiple high quality (and therefore pooled) estimates were available for Bulgaria, Croatia, France, Spain and the UK. Alongside a pooled estimate of 20.3% [47, 48], consecutive annual estimates report a decrease in anti-HCV prevalence in Spain from 44.9% in 2000 to 25.3% in 2009 [49]. Two multi-centre study-derived estimates from Bulgaria were pooled into an estimate of 26.3% (95% CI 23.5–29.3) [50, 51]. Four estimates from the UK were pooled into an overall prevalence of 17.4% (95% CI 16.4–18.4) [52–55]. The pooled prevalence of 6.3% for France is derived from seven studies screening more than 68,000 people in prison. The three estimates among juvenile inmates show considerable heterogeneity, from 20.5% prevalence in Bulgaria [46] to 8.6% in Germany [56] and 4.3% in Croatia [57]. To summarise, prevalence extracted (and pooled where possible) from the high quality studies ranged from 0.3% to 25.2% (for HBsAg) and from 4.3% to 86.3% (for anti-HCV).

### The prevalence of HBsAg and anti-HCV among PWID

The most recent, national level estimates of HBsAg and anti-HCV prevalence among PWID are presented in Table 1. National estimates of HBsAg prevalence were available for seven countries (Croatia, Cyprus, Greece, Hungary, Ireland, Latvia and Portugal), four of which were from studies conducted in 2013. The reported national prevalence ranges from 0.5% in Croatia, Hungary and Ireland, to more than 6% in Hungary and Portugal. National estimates of anti-HCV prevalence in the PWID population were available for 16 countries: Austria, Croatia, Cyprus, Czech Republic, Denmark, Finland, Greece, Hungary, Ireland, Italy, Latvia, Malta, Norway, Portugal, Slovenia and the UK. Anti-HCV prevalence was ≥30% in 13 of these countries and ≥50% in seven. In sum, the prevalence among PWID ranged from 0.5% to 6.1% (for HBsAg) and from 13.8% to 84.3% (for anti-HCV).

**Table 1:** HBsAg and anti-HCV prevalence in PWID in the EU/EEA

Country	HBsAg					Anti-HCV				
	Year	Sample size	Prevalence (95% CI)	Study design	Setting	Year	Sample size	Prevalence (95% CI)	Study design	Setting
Austria	–	–	–			2013	48	31.3 (18.7–46.3)	DT	ODD
Croatia	2007	200	0.5 (0.0–2.8)	SP	PRI	2007	200	44 (37.0–51.2)	SP	PRI
Cyprus	2013	82	6.1 (2.0–13.7)	DT	DTC	2013	82	47.6 (36.4–58.9)	DT	DTC
Czech Republic	–	–	–			2013	1889	14.6 (13.1–16.3)	DT	NSP
Denmark	–	–	–			2008	223	52.5 (45.7–59.2)	SP (UAT)	ODD
Finland	–	–	–			2009	682	60.5 (56.8–64.3)	SP (UAT)	NSP
Greece	2013	1337	3.0 (2.2–4.1)	DT	DTC; LTS; OTH; PHL; PRI; STR;	2013	1309	68.1 (65.5–70.6)	DT	DTC; LTS; PHL; PRI; OTH STR;
Hungary	2011	664	0.5 (0.1–1.3)	SP	DTC, NSP	2011	652	24.1 (20.8–27.6)	SP	DTC; NSP
Ireland	2010	200	0.5 (0.0–2.8)	SP	PRI	2010	200	41.5 (34.6–48.7)	SP	PRI
Italy	–	–	–			2010	743	60.5 (56.8–64.0)	DT	DTC
Latvia	2013	562	2.1 (1.1–3.7)	DT	DTC	2013	522	70.1 (66.0–74.0)	DT	NSP
Malta	–	–	–			2013	109	13.8 (7.9–21.7)	DT	ANT; DTC; HTC; OHC; PHL; STI
Norway	–		–			2013	6342	63.0 (61.8–64.2)	SP	DTC
Portugal	2013	399	6.3 (4.1–9.1)	DT	DTC	2013	414	84.3 (80.4–87.7)	DT	DTC
Slovenia	–	–	–			2009	112	32.1 (23.6–41.6)	DT	DTC
United Kingdom	–	–	–			2013	3144	49.1 (47.4–50.9)	SP (UAT)	DTC; LTS; OTH; NSP

Acronyms (study design): *DT* diagnostic testing, *SP* specific prevalence study, *UAT* unlinked anonymous testing

Acronyms (setting): *ANT* Antenatal Clinics**,** *DTC* Drug Treatment Centres, *HTC* HIV Testing Centres, *LTS* Low Threshold Services, *ODD* Overdose Deaths, *OHC* Other Hospitals or Clinics, *OTH* Other, *NSP* Needle Exchange Programmes, *PHL* Public Health Laboratories, *PRI* Prisons, *STI* STI clinics, *STR* Street

## Discussion

This is the first review to collate, assess and compare prevalence estimates across these three key at-risk groups in the EU/EEA. Although gaps in evidence exist, this study reports 68 HBsAg/anti-HCV single study/pooled prevalence estimates from 23 of 31 EU/EEA countries, 42 of which are considered as intermediate/high prevalence using the WHO endemicity threshold for HBV/HCV (≥2%) [58]. This includes 20 of the 23 estimates among PWID, 20 of the 28 high quality estimates among people in prison, and four of the 17 estimates among *HIV negative/unknown HIV sero-status* MSM. Geographical trends are difficult to determine due to heterogeneity of, and gaps in, evidence, although the reported data here are suggestive of higher prevalence among MSM (for anti-HCV) and among PWID (for both viruses) among countries in eastern and southern Europe.

Limitations in the estimates reported for people in prison and MSM relate to geographical and population coverage, study quality and heterogeneity of the included estimates. To retrieve estimates for people in prison and for MSM, we conducted a very broad search of the published literature with no language or population restrictions, and validated retrievals directly with countries, yet found many geographical gaps in the data. Indeed, only a third of EU/EEA countries are represented among the studies that met the inclusion criteria for people in prison and only seven countries reported estimates among MSM. It is unlikely we failed to identify and include all existing high quality data, and consider it most likely that the data just do not exist or are not published. In the absence of larger, more robust studies from which prevalence can be derived, we consider the data reported here are the best available although there may have been more recent estimates published since the date of our search (March 2015). Significant heterogeneity in study design within and between risk groups hamper the statistical comparison and pooling of prevalence across countries and populations. To control for strong sources of bias in studies among people in prison when pooling data, we developed and applied a study quality assessment. The five domains were considered equally important sources of bias and it is possible that estimates included in the analysis have residual selection biases. Further, our study quality assessment did not consider sample size and there is clearly more uncertainty in the estimates derived from smaller studies.

For pragmatic reasons, we extracted prevalence estimates for PWID from the data repository coordinated by EMCDDA. With limited methodological information accompanying the EMCDDA data sets, it is possible that this data set is not exhaustive. However, EMCDDA were recently identified by another wide-ranging systematic review as the source of the most routinely collected, European-level data on the viral hepatitis prevalence among PWID [18]. We adopted an algorithmic approach favouring the most recent national level data to select estimates, and found estimates meeting this criteria for just seven MS for HBsAg and 16 MS for anti-HCV. As with the retrievals from the systematic search, there is considerable heterogeneity in study design (intervention-related and observational), sampling method (single and multi-centre sampling methods) and sample size (from < 50 to > 6000 participants) across these 23 estimates. Beyond favouring the geographical and time-frame parameters, we did not systematically assess the quality of these studies and selection biases relating to study setting, population and sample size are likely to exist.

Using 2% prevalence as the endemicity threshold set out in the 2017 WHO HBV and HCV testing guidelines, [58] by comparing risk group prevalence with the general population prevalence (from previously published reviews of the literature [59–64]) and by comparing across risk groups, our findings generally support the continued classification of PWID and people in prison as the key populations for both chronic hepatitis B and C infection. Whilst this study does not seem to support the continued classification of *HIV negative/unknown HIV sero-status* MSM as a high (> 2%) prevalence population for chronic hepatitis B infection, we are cautious in this conclusion given the wide confidence interval (that sometimes includes the 2% threshold) around the MSM-derived HBV estimates. We therefore still consider MSM a key population for targeted action, given that anti-HCV prevalence in MSM is higher than in the general population, the evidence of the ongoing transmission of viral hepatitis among MSM populations and the complex interaction of viral hepatitis and HIV [11, 12]. Both infection with, and antiretroviral treatment for, HIV are suspected to increase progression to chronicity as well as to accelerate fibrosis [11, 12]. Global anti-HCV prevalence among HIV positive MSM has been estimated as high as 6.4% [65], and end-stage liver disease is a leading key cause of death among co-infected HIV positive patients in some high income countries [66]. A cohort study found an unexpectedly high proportion of MSM (23% compared to the 5–10% chronicity rate expected in the general adult population [67]) develop a chronic hepatitis B infection following HBV exposure regardless of HIV status, with a younger (adult) age at infection significantly associated with an increased risk of developing CHB [68]. It would therefore seem an effective use of health resources to (continue to) offer HCV screening to HIV positive men in addition to MSM-wide HBV vaccination [69]. Specific cost-effectiveness analyses of offering individual or combined blood-borne virus screening in MSM would also greatly aid public health decision making and be a useful addition to the evidence.

Differences in the prevalence among people in prison between countries are related to the differential distribution of risk factors among the prison population together with differences in prison conditions, such as the availability of harm reduction interventions and infection control practices and infrastructure across the EU/EEA countries represented in this study [16]. The high prevalence of HBsAg in the prison population in some countries could be attributable to the incarceration of people born in intermediate or high prevalence countries and consequent over-representation of migrants in the incarcerated population. Recent estimates suggest that the proportion of the prison population that is foreign-born ranges from < 5% in Bulgaria and Hungary, to more than 15% in France, Germany, Italy, Portugal and Spain, and up to 72% in Luxembourg [70]. It is possible that the incarceration of foreign-born migrants is a driver of the high prevalence of chronic hepatitis B and C infection in prisons although as there is no systematic EU-wide data collection on the demographic profile of the incarcerated population, our understanding of the dynamics of migration, incarceration and chronic infection is limited [70].

Our study seeks to contribute to the elimination of viral hepatitis in Europe by providing information to support the design and management of primary and secondary prevention strategies. However, expected prevalence is just one of a number of factors that affects the cost-effectiveness of testing strategies. Programme-related factors such as ease of reaching the target population, uptake of screening, actual (viraemic) prevalence, linkage to care and treatment initiation also playing a key role [9, 10, 3].

We see four key public health implications emerging from our experience in this study. The first is the indication for systematic screening and linkage to care of people in prison given the high prevalence, the overlap with the PWID population, and the possible continuation of risk behaviour. Secondly, we see significant public health benefits of providing treatment as prevention, especially for CHC, among populations that share risk behaviour, in line with national and international clinical and public health guidelines [58]. Analyses have shown that treatment among high-risk dynamically interactive populations such as PWIDs, is cost-effective, especially given the shorter and more tolerable treatment regimens [8]. Thirdly, the need for diagnostic testing and treatment is particularly important for PWID where the prevalence, and therefore risk of intra-population transmission, of hepatitis C is very high. Targeted PWID screening in accessible locations as part of broader harm reduction measures may help break down barriers of stigma and among this vulnerable and high risk population [16, 71]. The criminalisation of drug use has been suggested to be as the single most important determinant of the high blood-borne virus prevalence among people in prison [16]. Finally, the lower prevalence of CHB we found among some risk populations in some countries is likely a direct result of the adoption and implementation of primary prevention measures, especially childhood immunisation, in the general population [72]. This highlights the importance of adequately resourcing primary prevention measures as well as continuing to offer HBV vaccination to risk groups to protect public health.

The limitations of this study also provide ideas for future research, specifically the improvement in the design of studies and greater geographical representation to fill the gaps in evidence. The development and consistent application of an EU/EEA or international standard for the design and quality assessment of seroprevalence studies to inform pooling and/or statistical comparison of data across studies and populations would also greatly improve understanding of prevalence across countries and populations. Finally, and probably most importantly, there is clear and urgent need for more implementation studies to determine the features of screening programmes and strategies among risk populations that effectively reach, diagnose and link to care people infected with chronic viral hepatitis.

## Conclusion

Our study highlights the heterogeneity in prevalence across risk groups across Europe. Prevalence generally increases in an Eastern and Southern direction. There are also many countries, especially in the Eastern and Southern part of Europe, that are not represented in our results, highlighting the need to build capacity for and resource the development of robust epidemiological studies among key risk groups. Step One of elimination action planning is to know your epidemic, the ‘who’ and the ‘where’, [73] and both the evidence and the data gaps contained in this review should contribute to this strategic aim.

## Additional file

Additional file 1: Methodological details; summary results. Search strategy for studies in PubMed, Embase (Embase.com) and Cochrane Library (CDSR, DARE, HTA, EED); Definitions of subgroups included; Study inclusion and exclusion criteria; PRISMA Checklist; Reasons for exclusion of full text for studies in MSM and people in prison; Risk of bias framework for studies in MSM and among people in prison; Study characteristics for HBsAg/anti-HCV prevalence estimates in MSM and in people in prison. (DOCX 116 kb)

## Abbreviations

CHB: Chronic Hepatitis B
CHC: Chronic Hepatitis C
CIs: Confidence Intervals
DBS: Dried Blood Spot
ECDC: European Centre for Disease Prevention and Control
EMCDDA: European Centre for Drug Dependency and Addiction
EU/EEA: European Union/European Economic Area
HBV: Hepatitis B virus
HCV: Hepatitis C virus
MS: Member State
MSM: Men Who Have Sex With Men
NFP: National Focal Point
PRISMA: Preferred Reporting Items for Systematic Reviews and Meta-Analyses
PWID: People Who Inject Drugs
STIs: Sexually Transmitted Infections
HIV: Human Immuno-deficiency Virus
UK: United Kingdom
