# Supplementary material for: Hepatitis B/C in the countries of the EU/EEA: a systematic review of the prevalence among at-risk groups
Source: BMC Infect Dis. 2018 Feb 12;18:79. doi: 10.1186/s12879-018-2988-x (PMC5809955; doi:10.1186/s12879-018-2988-x)
Supplement: Supplementary file 1 — Methodological details; summary results. Search strategy for studies in PubMed, Embase (Embase.com) and Cochrane Library (CDSR, DARE, HTA, EED); Definitions of subgroups included; Study inclusion and exclusion criteria; PRISMA Checklist; Reasons for exclusion of full text for studies in MSM and people in prison; Risk of bias framework for studies in MSM and among people in prison; Study characteristics for HBsAg/anti-HCV prevalence estimates in MSM and in people in prison. (DOCX 116 kb) [file 12879_2018_2988_MOESM1_ESM.docx]

Additional file

Table of Contents

[Annex 1: Search strategy for studies reporting the prevalence of HBsAg/anti-HCV in the EU/EEA 3](#_Toc504483921)

[PubMed Search 3](#_Toc504483922)

[Embase search (Embase.com) 13](#_Toc504483923)

[Cochrane Library (CDSR, DARE, HTA, EED) 25](#_Toc504483924)

[Annex 2: List and definition of key subgroups included in the systematic review 28](#_Toc504483925)

[Annex 3: Study inclusion and exclusion criteria 29](#_Toc504483926)

[Annex 4: PRISMA Checklist 30](#_Toc504483927)

[Annex 5: Reasons for exclusion of full text for studies retrieved for MSM and prisoners 32](#_Toc504483928)

[Annex 6: Risk of bias framework for studies among MSM 34](#_Toc504483929)

[Annex 7. Risk of bias framework for studies among prisoners 34](#_Toc504483930)

[Annex 8: Study characteristics for HBsAg prevalence estimates in MSM 35](#_Toc504483931)

[Annex 9: Study characteristics for Anti-HCV prevalence estimates in MSM 36](#_Toc504483932)

[Annex 10: Study characteristics for HBsAg prevalence estimates in people in prison 37](#_Toc504483933)

[Annex 11: Study characteristics for anti-HCV prevalence estimates in people in prison 39](#_Toc504483934)

# Annex 1: Search strategy for studies reporting the prevalence of HBsAg/anti-HCV in the EU/EEA

## PubMed Search

Date of the search: 16/03/2015

Language limit: no limitis

Date limits: from 2005 to 2015

Number of results: 4541

| Search | Query | Items found |
| --- | --- | --- |
| [#4](http://www.ncbi.nlm.nih.gov/pubmed/advanced) | Search **#1 AND #2 AND #3** | [10461](http://www.ncbi.nlm.nih.gov/pubmed/?cmd=HistorySearch&querykey=4) |
| [#5](http://www.ncbi.nlm.nih.gov/pubmed/advanced) | Search **#1 AND #2 AND #3** Filters: **Publication date from 2005/01/01** | [4541](http://www.ncbi.nlm.nih.gov/pubmed/?cmd=HistorySearch&querykey=5) |
| [#3](http://www.ncbi.nlm.nih.gov/pubmed/advanced) | Search **"hepatitis B"[MeSH Terms] OR "hepatitis c"[MeSH Terms] OR "Hepatitis B virus"[Mesh] OR "hepacivirus"[Mesh] OR "hepatitis b"[TIAB] OR "hepatitis c"[TIAB] OR hepaciviru*[TIAB] OR "hbv"[TIAB] OR "hcv"[TIAB] OR "hbsag"[TIAB] OR "hbs ag"[TIAB] OR "hepatitis b surface antigens"[MeSH Terms] OR “Australia Antigen”[TIAB] OR “Australia Antigens”[TIAB] OR "hepatitis c antibodies"[MeSH Terms] OR "Hepatitis C Antigens"[Mesh] OR "Hepatitis B Antibodies"[Mesh]** | [135899](http://www.ncbi.nlm.nih.gov/pubmed/?cmd=HistorySearch&querykey=3) |
| [#2](http://www.ncbi.nlm.nih.gov/pubmed/advanced) | Search **"Prevalence"[Mesh] OR Prevalence*[TIAB] OR "Population Surveillance"[Mesh] OR "Seroepidemiologic Studies"[Mesh:NoExp] OR prevalence*[TIAB] OR seroepidemiolog*[TIAB] OR "sero epidemiologic"[TIAB] OR "sero epidemiological"[TIAB] OR "sero epidemiology"[TIAB] OR serosurvey*[TIAB] OR serolog*[TI] OR epidemiolog*[TI] OR surveillance[TI]** | [643490](http://www.ncbi.nlm.nih.gov/pubmed/?cmd=HistorySearch&querykey=2) |
| [#1](http://www.ncbi.nlm.nih.gov/pubmed/advanced) | Search **(((("United kingdom"[ad] OR Britain[ad] OR British[ad] OR (England[ad] NOT ("New England"[ad])) OR English[ad] OR Scotland[ad] OR Scottish[ad] OR Wales[ad] OR Welsh[ad] OR "Northen Ireland"[ad] OR London[ad] OR "East midlands"[ad] OR "West midlands"[ad] OR Yorkshire[ad] OR "East Anglia"[ad] OR Bedfordshire[ad] OR Hertfordshire[ad] OR Essex[ad] OR Peterborough[ad] OR Cambridgeshire[ad] OR Norfolk[ad] OR Suffolk[ad] OR Luton[ad] OR Bedford[ad] OR "Southend on sea"[ad] OR Thurrock[ad] OR Derbyshire[ad] OR Nottinghamshire[ad] OR Leicestershire[ad] OR Rutland[ad] OR Northamptonshire[ad] OR Lincolnshire[ad] OR Derby[ad] OR Leicester[ad] OR Northamptonshire[ad] OR Nottingham[ad] OR Northumberland[ad] OR "Tyne and Wear"[ad] OR "Tees Valley"[ad] OR "Durham"[ad] OR Darlington[ad] OR Hartlepool[ad] OR "Stockton on tees"[ad] OR Northumberland[ad] OR Teesside[ad] OR Sunderland[ad] OR Tyneside[ad] OR Cumbria[ad] OR Cheshire[ad] OR Manchester[ad] OR Lancashire[ad] OR Merseyside[ad] OR (Blackburn[ad] AND Darwen[ad]) OR Blackpool[ad] OR Chester[ad] OR Liverpool[ad] OR Sefton[ad] OR Warrington[ad] OR Wirral[ad] OR Berkshire[ad] OR Buckinghamshire[ad] OR Oxfordshire[ad] OR Hampshire[ad] OR "Isle of Wight"[ad] OR Kent[ad] OR Surrey[ad] OR Sussex[ad] OR (Brighton[ad] AND Hove[ad]) OR Medway[ad] OR "Milton keynes"[ad] OR Portsmouth[ad] OR Southampton[ad] OR Devon[ad] OR Dorset[ad] OR Somerset[ad] OR Gloucestershire[ad] OR Wiltshire[ad] OR Bristol[ad] OR Bath[ad] OR Bournemouth[ad] OR Poole[ad] OR Bristol[ad] OR Plymouth[ad] OR Swindon[ad] OR Torbay[ad] OR Herefordshire[ad] OR Worcestershire[ad] OR Warwickshire[ad] OR Shropshire[ad] OR Staffordshire[ad] OR Birmingham[ad] OR Coventry[ad] OR Dudley[ad] OR Sandwell[ad] OR Shropshire[ad] OR Solihull[ad] OR "stoke on trent"[ad] OR Telford[ad] OR Wrekin[ad] OR Walsall[ad] OR Warwickshire[ad] OR Wolverhampton[ad] OR Worcestershire[ad] OR Barnsley[ad] OR Doncaster[ad] OR Rotherham[ad] OR Bradford[ad] OR Calderdale[ad] OR Kirklees[ad] OR Kingston[ad] OR Leeds[ad] OR Sheffield[ad] OR Wakefield[ad] OR (York[ad] NOT ("New York"[ad])) OR Antrim[ad] OR Ards[ad] OR Armagh[ad] OR Ballymena[ad] OR Ballymoney[ad] OR Banbridge[ad] OR Carrickfergus[ad] OR Castlereagh[ad] OR Coleraine[ad] OR Cookstown[ad] OR Craigavon[ad] OR Derry[ad] OR (Down[ad] AND (district[ad] OR council[ad])) OR Fermanagh[ad] OR Dungannon[ad] OR Fermanagh[ad] OR Larne[ad] OR Limavady[ad] OR Lisburn[ad] OR Magherafelt[ad] OR Moyle[ad] OR (Newry[ad] AND Mourne[ad]) OR Newtownabbey[ad] OR Omagh[ad] OR Strabane[ad] OR Londonderry[ad] OR Tyrone[ad] OR Belfast[ad] OR Aberdeen[ad] OR Aberdeenshire[ad] OR Angus[ad] OR Dundee[ad] OR (Argyll[ad] AND bute[ad]) OR Clackmannanshire[ad] OR Fife[ad] OR Ayrshire[ad] OR Dunbartonshire[ad] OR Lothian[ad] OR Renfrewshire[ad] OR Edinburgh[ad] OR Falkirk[ad] OR Glasgow[ad] OR Highland*[ad] OR Inverclyde[ad] OR Midlothian[ad] OR Moray[ad] OR Lanarkshire[ad] OR (Perth[ad] AND Kinross[ad]) OR Stirling[ad] OR "Orkney Islands"[ad] OR "Eileanan Siar"[ad] OR "Shetland Islands"[ad] OR Bridgend[ad] OR "Neath Port Talbot"[ad] OR Cardiff[ad] OR (Vale[ad] AND Glamorgan[ad]) OR "Central Valleys"[ad] OR Conwy[ad] OR Denbighshire[ad] OR Flintshire[ad] OR Wrexham[ad] OR "Gwent Valleys"[ad] OR Gwynedd[ad] OR (Isle[ad] AND Anglesey[ad]) OR "Monmouthshire"[ad] OR "Newport"[ad] OR Powys[ad] OR Swansea[ad] OR Ceredigion[ad] OR Carmarthenshire[ad] OR Pembrokeshire[ad] OR "Merthyr Tydfil"[ad] OR "Rhondda Cynon Taff"[ad] OR "Blaenau Gwent"[ad] OR Caerphilly[ad] OR Torfaen[ad] OR Caithness[ad] OR "Sutherland and Ross"[ad] OR Cromarty[ad] OR Teeside[ad] OR Tyneside[ad] OR Wearside[ad] OR "West Mercia"[ad] OR Avon[ad] OR Ulster[ad] OR Derry[ad] OR Medway[ad] OR "East Riding"[ad] OR "West Riding"[ad] OR "Lake District"[ad] OR "Peak District"[ad] OR Cumberland[ad] OR Dartmoor[ad] OR Exmoor[ad])) OR ("United kingdom"[tw] OR Britain[tw] OR British[tw] OR (England[tw] NOT ("New England"[tw])) OR English[tw] OR Scotland[tw] OR Scottish[tw] OR Wales[tw] OR Welsh[tw] OR "Northen Ireland"[tw] OR London[tw] OR "East midlands"[tw] OR "West midlands"[tw] OR Yorkshire[tw] OR "East Anglia"[tw] OR Bedfordshire[tw] OR Hertfordshire[tw] OR Essex[tw] OR Peterborough[tw] OR Cambridgeshire[tw] OR Norfolk[tw] OR Suffolk[tw] OR Luton[tw] OR Bedford[tw] OR "Southend on sea"[tw] OR Thurrock[tw] OR Derbyshire[tw] OR Nottinghamshire[tw] OR Leicestershire[tw] OR Rutland[tw] OR Northamptonshire[tw] OR Lincolnshire[tw] OR Derby[tw] OR Leicester[tw] OR Northamptonshire[tw] OR Nottingham[tw] OR Northumberland[tw] OR "Tyne and Wear"[tw] OR "Tees Valley"[tw] OR "Durham"[tw] OR Darlington[tw] OR Hartlepool[tw] OR "Stockton on tees"[tw] OR Northumberland[tw] OR Teesside[tw] OR Sunderland[tw] OR Tyneside[tw] OR Cumbria[tw] OR Cheshire[tw] OR Manchester[tw] OR Lancashire[tw] OR Merseyside[tw] OR (Blackburn[tw] AND Darwen[tw]) OR Blackpool[tw] OR Chester[tw] OR Liverpool[tw] OR Sefton[tw] OR Warrington[tw] OR Wirral[tw] OR Berkshire[tw] OR Buckinghamshire[tw] OR Oxfordshire[tw] OR Hampshire[tw] OR "Isle of Wight"[tw] OR Kent[tw] OR Surrey[tw] OR Sussex[tw] OR (Brighton[tw] AND Hove[tw]) OR Medway[tw] OR "Milton keynes"[tw] OR Portsmouth[tw] OR Southampton[tw] OR Devon[tw] OR Dorset[tw] OR Somerset[tw] OR Gloucestershire[tw] OR Wiltshire[tw] OR Bristol[tw] OR Bath[tw] OR Bournemouth[tw] OR Poole[tw] OR Bristol[tw] OR Plymouth[tw] OR Swindon[tw] OR Torbay[tw] OR Herefordshire[tw] OR Worcestershire[tw] OR Warwickshire[tw] OR Shropshire[tw] OR Staffordshire[tw] OR Birmingham[tw] OR Coventry[tw] OR Dudley[tw] OR Sandwell[tw] OR Shropshire[tw] OR Solihull[tw] OR "stoke on trent"[tw] OR Telford[tw] OR Wrekin[tw] OR Walsall[tw] OR Warwickshire[tw] OR Wolverhampton[tw] OR Worcestershire[tw] OR Barnsley[tw] OR Doncaster[tw] OR Rotherham[tw] OR Bradford[tw] OR Calderdale[tw] OR Kirklees[tw] OR Kingston[tw] OR Leeds[tw] OR Sheffield[tw] OR Wakefield[tw] OR (York[tw] NOT ("New York"[tw])) OR Antrim[tw] OR Ards[tw] OR Armagh[tw] OR Ballymena[tw] OR Ballymoney[tw] OR Banbridge[tw] OR Carrickfergus[tw] OR Castlereagh[tw] OR Coleraine[tw] OR Cookstown[tw] OR Craigavon[tw] OR Derry[tw] OR (Down[tw] AND (district[tw] OR council[tw])) OR Fermanagh[tw] OR Dungannon[tw] OR Fermanagh[tw] OR Larne[tw] OR Limavady[tw] OR Lisburn[tw] OR Magherafelt[tw] OR Moyle[tw] OR (Newry[tw] AND Mourne[tw]) OR Newtownabbey[tw] OR Omagh[tw] OR Strabane[tw] OR Londonderry[tw] OR Tyrone[tw] OR Belfast[tw] OR Aberdeen[tw] OR Aberdeenshire[tw] OR Angus[tw] OR Dundee[tw] OR (Argyll[tw] AND bute[tw]) OR Clackmannanshire[tw] OR Fife[tw] OR Ayrshire[tw] OR Dunbartonshire[tw] OR Lothian[tw] OR Renfrewshire[tw] OR Edinburgh[tw] OR Falkirk[tw] OR Glasgow[tw] OR Highland*[tw] OR Inverclyde[tw] OR Midlothian[tw] OR Moray[tw] OR Lanarkshire[tw] OR (Perth[tw] AND Kinross[tw]) OR Stirling[tw] OR "Orkney Islands"[tw] OR "Eileanan Siar"[tw] OR "Shetland Islands"[tw] OR Bridgend[tw] OR "Neath Port Talbot"[tw] OR Cardiff[tw] OR (Vale[tw] AND Glamorgan[tw]) OR "Central Valleys"[tw] OR Conwy[tw] OR Denbighshire[tw] OR Flintshire[tw] OR Wrexham[tw] OR "Gwent Valleys"[tw] OR Gwynedd[tw] OR (Isle[tw] AND Anglesey[tw]) OR "Monmouthshire"[tw] OR "Newport"[tw] OR Powys[tw] OR Swansea[tw] OR Ceredigion[tw] OR Carmarthenshire[tw] OR Pembrokeshire[tw] OR "Merthyr Tydfil"[tw] OR "Rhondda Cynon Taff"[tw] OR "Blaenau Gwent"[tw] OR Caerphilly[tw] OR Torfaen[tw] OR Caithness[tw] OR "Sutherland and Ross"[tw] OR Cromarty[tw] OR Teeside[tw] OR Tyneside[tw] OR Wearside[tw] OR "West Mercia"[tw] OR Avon[tw] OR Ulster[tw] OR Derry[tw] OR Medway[tw] OR "East Riding"[tw] OR "West Riding"[tw] OR "Lake District"[tw] OR "Peak District"[tw] OR Cumberland[tw] OR Dartmoor[tw] OR Exmoor[tw])) OR (((Sweden[ad] OR Sverige[ad] OR Swedish[ad] OR Svenska[ad] OR Stockholm*[ad] OR Norrland[ad] OR Svealand[ad] OR Mellansverige[ad] OR Smaland[ad] OR Sydsverige[ad] OR Vastsverige[ad] OR Orebro[ad] OR Ostergotland*[ad] OR Vastergotland*[ad] OR Skara*[ad] OR Bohus*[ad] OR Dalsland[ad] OR Narke[ad] OR Sodermanland[ad] OR Uppsala[ad] OR Uppland[ad] OR Vastmanland*[ad] OR Jamtland*[ad] OR Harjedalen[ad] OR Vasternorrland*[ad] OR Dalarna[ad] OR Kopparberg[ad] OR Gavleborg*[ad] OR Gastrikland[ad] OR Halsingland[ad] OR Varmland*[ad] OR Gotland*[ad] OR Oland[ad] OR Jonkoping*[ad] OR Kalmar*[ad] OR Kronoberg*[ad] OR Blekinge[ad] OR Skane*[ad] OR Norrbotten*[ad] OR Vasterbotten*[ad] OR Lappland[ad] OR Angermanland[ad] OR Medelpad[ad] OR Halland*[ad] OR Gotaland*[ad] OR Gothenburg[ad] OR Goteborg*[ad] OR Malmo*[ad] OR Vasteras[ad] OR Linkoping[ad] OR Helsingborg[ad] OR Halsingborg[ad] OR Norrkoping[ad] OR Gavle[ad] OR Umea[ad] OR Lulea[ad] OR Karlstad[ad] OR Kalmar[ad] OR Huddinge[ad] OR Solna[ad] OR Ostersjo*[ad] OR Malaren*[ad] OR Malardalen[ad])) OR (Sweden[tw] OR Sverige[tw] OR Swedish[tw] OR Svenska[tw] OR Stockholm*[tw] OR Norrland[tw] OR Svealand[tw] OR Mellansverige[tw] OR Smaland[tw] OR Sydsverige[tw] OR Vastsverige[tw] OR Orebro[tw] OR Ostergotland*[tw] OR Vastergotland*[tw] OR Skara*[tw] OR Bohus*[tw] OR Dalsland[tw] OR Narke[tw] OR Sodermanland[tw] OR Uppsala[tw] OR Uppland[tw] OR Vastmanland*[tw] OR Jamtland*[tw] OR Harjedalen[tw] OR Vasternorrland*[tw] OR Dalarna[tw] OR Kopparberg[tw] OR Gavleborg*[tw] OR Gastrikland[tw] OR Halsingland[tw] OR Varmland*[tw] OR Gotland*[tw] OR Oland[tw] OR Jonkoping*[tw] OR Kalmar*[tw] OR Kronoberg*[tw] OR Blekinge[tw] OR Skane*[tw] OR Norrbotten*[tw] OR Vasterbotten*[tw] OR Lappland[tw] OR Angermanland[tw] OR Medelpad[tw] OR Halland*[tw] OR Gotaland*[tw] OR Gothenburg[tw] OR Goteborg*[tw] OR Malmo*[tw] OR Vasteras[tw] OR Linkoping[tw] OR Helsingborg[tw] OR Halsingborg[tw] OR Norrkoping[tw] OR Gavle[tw] OR Umea[tw] OR Lulea[tw] OR Karlstad[tw] OR Kalmar[tw] OR Huddinge[tw] OR Solna[tw] OR Ostersjo*[tw] OR Malaren*[tw] OR Malardalen[tw])) OR (((Spain[ad] OR Espana[ad] OR Spanish[ad] OR Espanol*[ad] OR Spaniard*[ad] OR Madrid[ad] OR Andalucia[ad] OR Andalusia[ad] OR Aragon[ad] OR Cantabria[ad] OR Canarias[ad] OR "Canary Islands"[ad] OR "Castile and leon"[ad] OR "Castilla y Leon"[ad] OR "Castile La Mancha"[ad] OR "Castilla La Mancha"[ad] OR Cataluna[ad] OR Catalonia[ad] OR Ceuta[ad] OR Melilla[ad] OR Navarra[ad] OR Navarre[ad] OR Valencia[ad] OR Valencian[ad] OR Extremadura[ad] OR Galicia[ad] OR Balears[ad] OR "Balearic Islands"[ad] OR Baleares[ad] OR "La Rioja"[ad] OR "Pais Vasco"[ad] OR "Basque Country"[ad] OR Asturias[ad] OR Murcia[ad] OR Coruna[ad] OR Alava[ad] OR Araba[ad] OR Albacete[ad] OR Alicante[ad] OR Alacant[ad] OR Almeria[ad] OR Asturias[ad] OR Avila[ad] OR Badajoz[ad] OR Badajos[ad] OR Barcelona[ad] OR Burgos[ad] OR Caceres[ad] OR Cadiz[ad] OR Castellon[ad] OR Castello[ad] OR "Ciudad Real"[ad] OR Cordoba[ad] OR Cuenca[ad] OR Eivissa[ad] OR Ibiza[ad] OR Formentera[ad] OR "El Hierro"[ad] OR Fuerteventura[ad] OR Girona[ad] OR Gerona[ad] OR "Gran Canaria"[ad] OR Granada[ad] OR Guadalajara[ad] OR Guipuzcoa[ad] OR Gipuzkoa[ad] OR Huelva[ad] OR Huesca[ad] OR Jaen[ad] OR "La Gomera"[ad] OR "La Palma"[ad] OR Lanzarote[ad] OR Leon[ad] OR Lleida[ad] OR Lerida[ad] OR Lugo[ad] OR Malaga[ad] OR Mallorca[ad] OR Majorca[ad] OR Menorca[ad] OR Minorca[ad] OR Murcia[ad] OR Ourense[ad] OR Orense[ad] OR Palencia[ad] OR Pontevedra[ad] OR Salamanca[ad] OR Segovia[ad] OR Sevilla[ad] OR Seville[ad] OR Soria[ad] OR Tarragona[ad] OR Tenerife[ad] OR Teruel[ad] OR Toledo[ad] OR Valencia[ad] OR Valladolid[ad] OR Vizcaya[ad] OR Biscay[ad] OR Zamora[ad] OR Zaragoza[ad] OR Saragossa[ad] OR Bilbao[ad] OR Bilbo[ad] OR Compostela[ad] OR "San Sebastian"[ad] OR Donostia[ad] OR Vitoria[ad] OR Oviedo[ad] OR Pamplona[ad] OR Logrono[ad] OR Gasteiz[ad])) OR (Spain[tw] OR Espana[tw] OR Spanish[tw] OR Espanol*[tw] OR Spaniard*[tw] OR Madrid[tw] OR Andalucia[tw] OR Andalusia[tw] OR Aragon[tw] OR Cantabria[tw] OR Canarias[tw] OR "Canary Islands"[tw] OR "Castile and leon"[tw] OR "Castilla y Leon"[tw] OR "Castile La Mancha"[tw] OR "Castilla La Mancha"[tw] OR Cataluna[tw] OR Catalonia[tw] OR Ceuta[tw] OR Melilla[tw] OR Navarra[tw] OR Navarre[tw] OR Valencia[tw] OR Valencian[tw] OR Extremadura[tw] OR Galicia[tw] OR Balears[tw] OR "Balearic Islands"[tw] OR Baleares[tw] OR "La Rioja"[tw] OR "Pais Vasco"[tw] OR "Basque Country"[tw] OR Asturias[tw] OR Murcia[tw] OR Coruna[tw] OR Alava[tw] OR Araba[tw] OR Albacete[tw] OR Alicante[tw] OR Alacant[tw] OR Almeria[tw] OR Asturias[tw] OR Avila[tw] OR Badajoz[tw] OR Badajos[tw] OR Barcelona[tw] OR Burgos[tw] OR Caceres[tw] OR Cadiz[tw] OR Castellon[tw] OR Castello[tw] OR "Ciudad Real"[tw] OR (Cordoba[tw] NOT Argent*[tw]) OR Cuenca[tw] OR Eivissa[tw] OR Ibiza[tw] OR Formentera[tw] OR "El Hierro"[tw] OR Fuerteventura[tw] OR Girona[tw] OR Gerona[tw] OR "Gran Canaria"[tw] OR Granada[tw] OR (Guadalajara[tw] NOT Mexic*[tw]) OR Guipuzcoa[tw] OR Gipuzkoa[tw] OR Huelva[tw] OR Huesca[tw] OR Jaen[tw] OR "La Gomera"[tw] OR "La Palma"[tw] OR Lanzarote[tw] OR Leon[tw] OR Lleida[tw] OR Lerida[tw] OR Lugo[tw] OR Malaga[tw] OR Mallorca[tw] OR Majorca[tw] OR Menorca[tw] OR Minorca[tw] OR Murcia[tw] OR Ourense[tw] OR Orense[tw] OR Palencia[tw] OR Pontevedra[tw] OR Salamanca[tw] OR Segovia[tw] OR Sevilla[tw] OR Seville[tw] OR Soria[tw] OR Tarragona[tw] OR Tenerife[tw] OR Teruel[tw] OR Toledo[tw] OR Valencia[tw] OR Valladolid[tw] OR Vizcaya[tw] OR Biscay[tw] OR Zamora[tw] OR Zaragoza[tw] OR Saragossa[tw] OR Bilbao[tw] OR Bilbo[tw] OR Compostela[tw] OR "San Sebastian"[tw] OR Donostia[tw] OR Vitoria[tw] OR Oviedo[tw] OR Pamplona[tw] OR Logrono[tw] OR Gasteiz[tw])) OR (((Slovenia*[ad] OR Slovenija[ad] OR slovensk*[ad] OR Ljubljana[ad] OR Gorenjska[ad] OR Carniola[ad] OR Goriska[ad] OR Gorizia[ad] OR Koroska[ad] OR Carinthia[ad] OR "Notranjsko kraska"[ad] OR "Obalno kraska"[ad] OR "Coastal krast"[ad] OR Podravska[ad] OR Pomurska[ad] OR Savinjska[ad] OR Spodnjeposavska[ad] OR Zasavska[ad] OR Osrednjeslovenska[ad] OR Maribor[ad] OR Celje[ad] OR Kranj[ad] OR Velenje[ad] OR Koper[ad] OR Capodistria[ad] OR "Novo mesto"[ad] OR Ptuj[ad] OR Trbovlje[ad] OR Kamnik[ad] OR Murska[ad] OR Sobota[ad] OR "Nova Gorica"[ad])) OR (Slovenia*[tw] OR Slovenija[tw] OR slovensk*[tw] OR Ljubljana[tw] OR Gorenjska[tw] OR Carniola[tw] OR Goriska[tw] OR Gorizia[tw] OR Koroska[tw] OR Carinthia[tw] OR "Notranjsko kraska"[tw] OR "Obalno kraska"[tw] OR "Coastal krast"[tw] OR Podravska[tw] OR Pomurska[tw] OR Savinjska[tw] OR Spodnjeposavska[tw] OR Zasavska[tw] OR Osrednjeslovenska[tw] OR Maribor[tw] OR Celje[tw] OR Kranj[tw] OR Velenje[tw] OR Koper[tw] OR Capodistria[tw] OR "Novo mesto"[tw] OR Ptuj[tw] OR Trbovlje[tw] OR Kamnik[tw] OR Murska[tw] OR Sobota[tw] OR "Nova Gorica"[tw])) OR ((Slovakia[tw] OR Slovensk*[tw] OR Slovak*[tw] OR Bratislav*[tw] OR Trnav*[tw] OR Trnava[tw] OR Nitrian*[tw] OR Nitra[tw] OR Trencian*[tw] OR Trencin[tw] OR Banskobystri*[tw] OR "Banska Bystrica"[tw] OR Zilina[tw] OR Zilin*[tw] OR Trnava[tw] OR Trnav*[tw] OR Presov[tw] OR Presov*[tw] OR Kosic*[tw] OR (Martin[tw] AND (city[tw] OR Svaty[tw])) OR Poprad[tw])) OR (Slovakia[ad] OR Slovensk*[ad] OR Slovak*[ad] OR Bratislav*[ad] OR Trnav*[ad] OR Nitrian*[ad] OR Nitra[ad] OR Trencian*[ad] OR Trencin[ad] OR Banskobystri*[ad] OR "Banska Bystrica"[ad] OR Zilina[ad] OR Zilin*[ad] OR Trnava[ad] OR Trnav*[ad] OR Presov*[ad] OR Kosic*[ad] OR (Martin[ad] AND (city[ad] OR Svaty[ad])) OR Poprad[ad])) OR ((((Romania[tw] OR Rumania[tw] OR Roumania[tw] OR Romanian[tw] OR Roman[tw] OR Bucharest[tw] OR Bucuresti[tw] OR Alba[tw] OR Brasov[tw] OR Covasna[tw] OR Harghita[tw] OR Mures[tw] OR Sibiu[tw] OR Bacau[tw] OR Botosani[tw] OR Iasi[tw] OR Neamt[tw] OR Suceava[tw] OR Vaslui[tw] OR Bihor[tw] OR "Bistrita Nasaud"[tw] OR Cluj[tw] OR Maramures[tw] OR Salaj[tw] OR "Satu Mare"[tw] OR Arges[tw] OR Calarasi[tw] OR Dambovita[tw] OR Giurgiu[tw] OR Ialomita[tw] OR Prahova[tw] OR Teleorman[tw] OR Braila[tw] OR Buzau[tw] OR Constanta[tw] OR Galati[tw] OR Tulcea[tw] OR Vrancea[tw] OR Dolj[tw] OR Gorj[tw] OR Mehedinti[tw] OR (Olt[tw] AND (river[tw] OR county[tw] OR region[tw] OR judetul[tw] OR Raul[tw])) OR Valcea[tw] OR Vilcea[tw] OR Arad[tw] OR "Caras-Severin"[tw] OR Hunedoara[tw] OR Timis[tw] OR Ilfov[tw] OR Timisoara[tw] OR Constanta[tw] OR Craiova[tw] OR Ploiesti[tw] OR Oradea[tw] OR "Cluj-Napoca"[tw] OR Deva[tw])) OR (Romania[ad] OR Rumania[ad] OR Roumania[ad] OR Romanian[ad] OR Roman[ad] OR Bucharest[ad] OR Bucuresti[ad] OR Alba[ad] OR Brasov[ad] OR Covasna[ad] OR Harghita[ad] OR Mures[ad] OR Sibiu[ad] OR Bacau[ad] OR Botosani[ad] OR Iasi[ad] OR Neamt[ad] OR Suceava[ad] OR Vaslui[ad] OR Bihor[ad] OR "Bistrita Nasaud"[ad] OR Cluj[ad] OR Maramures[ad] OR Salaj[ad] OR "Satu Mare"[ad] OR Arges[ad] OR Calarasi[ad] OR Dambovita[ad] OR Giurgiu[ad] OR Ialomita[ad] OR Prahova[ad] OR Teleorman[ad] OR Braila[ad] OR Buzau[ad] OR Constanta[ad] OR Galati[ad] OR Tulcea[ad] OR Vrancea[ad] OR Dolj[ad] OR Gorj[ad] OR Mehedinti[ad] OR (Olt[ad] AND (river[ad] OR county[ad] OR region[ad] OR judetul[ad] OR Raul[ad])) OR Valcea[ad] OR Vilcea[ad] OR Arad[ad] OR "Caras-Severin"[ad] OR Hunedoara[ad] OR Timis[ad] OR Ilfov[ad] OR Timisoara[ad] OR Constanta[ad] OR Craiova[ad] OR Ploiesti[ad] OR Oradea[ad] OR "Cluj-Napoca"[ad] OR Deva[ad])) OR (((Portugal[tw] OR Portugues*[tw] OR Lisboa[tw] OR Lisbon[tw] OR Leira[tw] OR Santarem[tw] OR Beja[tw] OR Faro[tw] OR Evora[tw] OR Portalegre[tw] OR "Castelo Branco"[tw] OR Guarda[tw] OR Aveiro[tw] OR Viseu[tw] OR Braganca[tw] OR "Vila real"[tw] OR "Viana do Castelo"[tw] OR Alentejo[tw] OR Azores[tw] OR Acores[tw] OR Madeira[tw] OR "Os Montes"[tw] OR (Ave[tw] AND (community[tw] OR intermunicipal[tw] OR comunidade[tw])) OR Mondego[tw] OR Vouga[tw] OR Beira[tw] OR Cavado[tw] OR Lafoes[tw] OR Douro[tw] OR Porto[tw] OR Tejo[tw] OR Minho[tw] OR Setubal[tw] OR Pinhal[tw] OR "Serra da Estrela"[tw] OR Tamega[tw] OR Algarve[tw] OR Gaia[tw] OR Amadora[tw] OR Braga[tw] OR (Agualva[tw] AND Cacem[tw]) OR Funchal[tw] OR Coimbra[tw] OR Almada[tw])) OR (Portugal[ad] OR Portugues*[ad] OR Lisboa[ad] OR Lisbon[ad] OR Leira[ad] OR Santarem[ad] OR Beja[ad] OR Faro[ad] OR Evora[ad] OR Portalegre[ad] OR "Castelo Branco"[ad] OR Guarda[ad] OR Aveiro[ad] OR Viseu[ad] OR Braganca[ad] OR "Vila real"[ad] OR "Viana do Castelo"[ad] OR Alentejo[ad] OR Azores[ad] OR Acores[ad] OR Madeira[ad] OR "Os Montes"[ad] OR (Ave[ad] AND (community[ad] OR intermunicipal[ad] OR comunidade[ad])) OR Mondego[ad] OR Vouga[ad] OR Beira[ad] OR Cavado[ad] OR Lafoes[ad] OR Douro[ad] OR Porto[ad] OR Tejo[ad] OR Minho[ad] OR Setubal[ad] OR Pinhal[ad] OR "Serra da Estrela"[ad] OR Tamega[ad] OR Algarve[ad] OR Gaia[ad] OR Amadora[ad] OR Braga[ad] OR (Agualva[ad] AND Cacem[ad]) OR Funchal[ad] OR Coimbra[ad] OR Almada[ad])) OR (((Poland[ad] OR Polska[ad] OR Polish[ad] OR Polski[ad] OR Pole[ad] OR Poles[ad] OR Polak[ad] OR Polka[ad] OR Polacy[ad] OR Polacy[ad] OR Warsaw[ad] OR Warszawa[ad] OR Wielkopolskie[ad] OR Pomerania*[ad] OR Pomorskie[ad] OR Kuyavian[ad] OR Kujawsko[ad] OR Malopolskie[ad] OR Lodz[ad] OR Lodzkie[ad] OR Silesia*[ad] OR Slask[ad] OR Dolnoslaskie[ad] OR Lublin[ad] OR Lubelskie[ad] OR Lubus[ad] OR Lubusz[ad] OR Lubuskie[ad] OR Masovia[ad] OR Mazowske[ad] OR Masovian[ad] OR Mazowieckie[ad] OR Opole[ad] OR Opolskie[ad] OR Podlaskie[ad] OR Podlachia[ad] OR Podlasie[ad] OR Subcarpathian*[ad] OR Carpathian*[ad] OR Podkarpackie[ad] OR Swietokrzyskie[ad] OR Slaskie[ad] OR Slask[ad] OR "Varmia Mazuria"[ad] OR "Varmian Mazurian"[ad] OR "Varmia Masuria"[ad] OR "Varmian Masurian"[ad] OR "Warmia Mazury"[ad] OR "Warminsko Mazurskie"[ad] OR Zachodniopomorskie[ad] OR Krakow[ad] OR Cracow[ad] OR Wroclaw[ad] OR Poznan[ad] OR Gdansk[ad] OR Szczecin[ad] OR Bydgoszcz[ad] OR Katowice[ad] OR Bialystok[ad] OR Olsztyn[ad] OR Kielce[ad] OR "Zielona Gora"[ad] OR Torun[ad] OR "Gorzow Wielkopolski"[ad])) OR (Poland[tw] OR Polska[tw] OR Polish[tw] OR Polski[tw] OR Pole[tw] OR Poles[tw] OR Polak[tw] OR Polka[tw] OR Polacy[tw] OR Polacy[tw] OR Warsaw[tw] OR Warszawa[tw] OR Wielkopolskie[tw] OR Pomerania*[tw] OR Pomorskie[tw] OR Kuyavian[tw] OR Kujawsko[tw] OR Malopolskie[tw] OR Lodz[tw] OR Lodzkie[tw] OR Silesia*[tw] OR Slask[tw] OR Dolnoslaskie[tw] OR Lublin[tw] OR Lubelskie[tw] OR Lubus[tw] OR Lubusz[tw] OR Lubuskie[tw] OR Masovia[tw] OR Mazowske[tw] OR Masovian[tw] OR Mazowieckie[tw] OR Opole[tw] OR Opolskie[tw] OR Podlaskie[tw] OR Podlachia[tw] OR Podlasie[tw] OR Subcarpathian*[tw] OR Carpathian*[tw] OR Podkarpackie[tw] OR Swietokrzyskie[tw] OR Slaskie[tw] OR Slask[tw] OR "Varmia Mazuria"[tw] OR "Varmian Mazurian"[tw] OR "Varmia Masuria"[tw] OR "Varmian Masurian"[tw] OR "Warmia Mazury"[tw] OR "Warminsko Mazurskie"[tw] OR Zachodniopomorskie[tw] OR Krakow[tw] OR Cracow[tw] OR Wroclaw[tw] OR Poznan[tw] OR Gdansk[tw] OR Szczecin[tw] OR Bydgoszcz[tw] OR Katowice[tw] OR Bialystok[tw] OR Olsztyn[tw] OR Kielce[tw] OR "Zielona Gora"[tw] OR Torun[tw] OR "Gorzow Wielkopolski"[tw])) OR (((Netherlands[ad] OR Nederland*[ad] OR Dutch*[ad] OR Amsterdam[ad] OR Drenthe[ad] OR Flevoland[ad] OR Friesland[ad] OR Fryslan[ad] OR Gelderland[ad] OR Guelders[ad] OR Groningen[ad] OR Limburg[ad] OR "North Brabant"[ad] OR "Noord Brabant"[ad] OR Holland[ad] OR Overijssel[ad] OR Overissel[ad] OR Utrecht[ad] OR Zeeland[ad] OR Rotterdam[ad] OR Hague[ad] OR Eindhoven[ad] OR Tilburg[ad] OR Almere[ad] OR Breda[ad] OR Nijmegen[ad] OR Nimeguen[ad])) OR (Netherlands[tw] OR Nederland*[tw] OR Dutch*[tw] OR Amsterdam[tw] OR Drenthe[tw] OR Flevoland[tw] OR Friesland[tw] OR Fryslan[tw] OR Gelderland[tw] OR Guelders[tw] OR Groningen[tw] OR Limburg[tw] OR "North Brabant"[tw] OR "Noord Brabant"[tw] OR Holland[tw] OR Overijssel[tw] OR Overissel[tw] OR Utrecht[tw] OR Zeeland[tw] OR Rotterdam[tw] OR Hague[tw] OR Eindhoven[tw] OR Tilburg[tw] OR Almere[tw] OR Breda[tw] OR Nijmegen[tw] OR Nimeguen[tw])) OR (Malta[tw] OR Maltese[tw] OR Valletta[tw] OR Gozo[tw] OR Ghawdex[tw] OR Malta[ad] OR Maltese[ad] OR Valletta[ad] OR Gozo[ad] OR Ghawdex[ad]) OR (Luxembourg*[tw] OR Luxemburg[tw] OR Letzebuerg[tw] OR Diekirch[tw] OR Grevenmacher[tw] OR Luxembourg*[ad] OR Luxemburg[ad] OR Letzebuerg[ad] OR Diekirch[ad] OR Grevenmacher[ad]) OR (((Lithuania*[tw] OR "Lietuvos Respublika"[tw] OR Lietuva[tw] OR lietuviu[tw] OR Vilnius[tw] OR Vilniaus[tw] OR Kaunas[tw] OR Kauno[tw] OR Klaipeda[tw] OR Klaipedos[tw] OR Panevezys[tw] OR Panevezio[tw] OR Siauliai[tw] OR Siauliu[tw] OR Alytus[tw] OR Alytaus[tw] OR Taurages[tw] OR Taurage[tw] OR Marijampoles[tw] OR Marijampole[tw] OR Telsiu[tw] OR Telsiai[tw] OR Utenos[tw] OR Utena[tw] OR Mazeikiai[tw] OR Jonava[tw] OR Mazeikiu[tw] OR Jonavos[tw])) OR (Lithuania*[ad] OR "Lietuvos Respublika"[ad] OR Lietuva[ad] OR lietuviu[ad] OR Vilnius[ad] OR Vilniaus[ad] OR Kaunas[ad] OR Kauno[ad] OR Klaipeda[ad] OR Klaipedos[ad] OR Panevezys[ad] OR Panevezio[ad] OR Siauliai[ad] OR Siauliu[ad] OR Alytus[ad] OR Alytaus[ad] OR Taurages[ad] OR Taurage[ad] OR Marijampoles[ad] OR Marijampole[ad] OR Telsiu[ad] OR Telsiai[ad] OR Utenos[ad] OR Utena[ad] OR Mazeikiai[ad] OR Jonava[ad] OR Mazeikiu[ad] OR Jonavos[ad])) OR (((Latvi*[tw] OR Latvija*[tw] OR Riga[tw] OR Courland[tw] OR Kurzeme[tw] OR Kurland[tw] OR Latgale[tw] OR Lettgallia[tw] OR Latgola[tw] OR Vidzeme[tw] OR Vidumo[tw] OR Semigallia[tw] OR Semigalia[tw] OR Zemgale[tw] OR Pieriga[tw] OR Daugavpils[tw] OR Dinaburg[tw] OR Liepaja[tw] OR Libau[tw] OR Jelgava[tw] OR Jurmala[tw] OR Jekabpils[tw] OR Jakobstadt[tw] OR Rezekne[tw] OR Rezne[tw] OR Rositten[tw] OR Valmiera[tw] OR Wolmar[tw] OR Ventspils[tw] OR Windau[tw])) OR (Latvi*[ad] OR Latvija*[ad] OR Riga[ad] OR Courland[ad] OR Kurzeme[ad] OR Kurland[ad] OR Latgale[ad] OR Lettgallia[ad] OR Latgola[ad] OR Vidzeme[ad] OR Vidumo[ad] OR Semigallia[ad] OR Semigalia[ad] OR Zemgale[ad] OR Pieriga[ad] OR Daugavpils[ad] OR Dinaburg[ad] OR Liepaja[ad] OR Libau[ad] OR Jelgava[ad] OR Jurmala[ad] OR Jekabpils[ad] OR Jakobstadt[ad] OR Rezekne[ad] OR Rezne[ad] OR Rositten[ad] OR Valmiera[ad] OR Wolmar[ad] OR Ventspils[ad] OR Windau[ad]))) OR ((((Italy[tw] OR Italia*[tw] OR Rome[tw] OR Roma[tw] OR Abruzzo[tw] OR Abruzzi[tw] OR Basilicata[tw] OR Lucania[tw] OR Calabria[tw] OR Campania[tw] OR "Emilia Romagna"[tw] OR "friuli venezia giulia"[tw] OR Lazio[tw] OR Latium[tw] OR Liguria*[tw] OR Lombardy[tw] OR Lombardia[tw] OR Marche[tw] OR Marches[tw] OR Molisano[tw] OR Molise[tw] OR Piedmont*[tw] OR Piemonte[tw] OR Sardinia[tw] OR Sardegna[tw] OR Sicily[tw] OR Sicilia[tw] OR Toscana[tw] OR Tuscany[tw] OR Trentino[tw] OR Trento[tw] OR Umbria[tw] OR Veneto[tw] OR Triveneto[tw] OR Puglia[tw] OR Apulia[tw] OR Bolzano[tw] OR Bozen[tw] OR Milan[tw] OR Milano[tw] OR Naples[tw] OR Napoli[tw] OR Turin[tw] OR Torino[tw] OR Palermo[tw] OR Genoa[tw] OR Genova[tw] OR Bologna[tw] OR Florence[tw] OR Firenze[tw] OR Bari[tw] OR Catania[tw] OR Venezia[tw] OR Venice[tw] OR Padova[tw] OR Padua[tw] OR Siena[tw] OR Bologna[tw] OR Trieste[tw] OR Urbino[tw] OR Aosta[tw] OR Aoste[tw] OR Perugia[tw] OR Brescia[tw] OR Cagliari[tw] OR Catanzaro[tw] OR "L Aquila"[tw] OR Ancona[tw])) OR (Italy[ad] OR Italia*[ad] OR Rome[ad] OR Roma[ad] OR Abruzzo[ad] OR Abruzzi[ad] OR Basilicata[ad] OR Lucania[ad] OR Calabria[ad] OR Campania[ad] OR "Emilia Romagna"[ad] OR "friuli venezia giulia"[ad] OR Lazio[ad] OR Latium[ad] OR Liguria*[ad] OR Lombardy[ad] OR Lombardia[ad] OR Marche[ad] OR Marches[ad] OR Molisano[ad] OR Molise[ad] OR Piedmont*[ad] OR Piemonte[ad] OR Sardinia[ad] OR Sardegna[ad] OR Sicily[ad] OR Sicilia[ad] OR Toscana[ad] OR Tuscany[ad] OR Trentino[ad] OR Trento[ad] OR Umbria[ad] OR Veneto[ad] OR Triveneto[ad] OR Puglia[ad] OR Apulia[ad] OR Bolzano[ad] OR Bozen[ad] OR Milan[ad] OR Milano[ad] OR Naples[ad] OR Napoli[ad] OR Turin[ad] OR Torino[ad] OR Palermo[ad] OR Genoa[ad] OR Genova[ad] OR Bologna[ad] OR Florence[ad] OR Firenze[ad] OR Bari[ad] OR Catania[ad] OR Venezia[ad] OR Venice[ad] OR Padova[ad] OR Padua[ad] OR Siena[ad] OR Bologna[ad] OR Trieste[ad] OR Urbino[ad] OR Aosta[ad] OR Aoste[ad] OR Perugia[ad] OR Brescia[ad] OR Cagliari[ad] OR Catanzaro[ad] OR "L Aquila"[ad] OR Ancona[ad])) OR (((Ireland[tw] OR Eire[tw] OR Irish*[tw] OR Dublin[tw] OR Fingal[tw] OR "Dun Laoghaire"[tw] OR Wicklow[tw] OR Wexford[tw] OR Carlow[tw] OR Kildare[tw] OR Meath[tw] OR Louth[tw] OR Monaghan[tw] OR Cavan[tw] OR Longford[tw] OR Westmeath[tw] OR Offaly[tw] OR Laois[tw] OR Kilkenny[tw] OR Waterford[tw] OR Cork[tw] OR Kerry[tw] OR Limerick[tw] OR Tipperary[tw] OR Clare[tw] OR Galway[tw] OR Mayo[tw] OR Roscommon[tw] OR Sligo[tw] OR Leitrim[tw] OR Donegal[tw] OR Drogheda[tw] OR Dundalk[tw] OR Swords[tw] OR Bray[tw] OR Navan[tw] OR Munster[tw] OR Leinster[tw] OR Connacht[tw])) OR (Ireland[ad] OR Eire[ad] OR Irish*[ad] OR Dublin[ad] OR Fingal[ad] OR "Dun Laoghaire"[ad] OR Wicklow[ad] OR Wexford[ad] OR Carlow[ad] OR Kildare[ad] OR Meath[ad] OR Louth[ad] OR Monaghan[ad] OR Cavan[ad] OR Longford[ad] OR Westmeath[ad] OR Offaly[ad] OR Laois[ad] OR Kilkenny[ad] OR Waterford[ad] OR Cork[ad] OR Kerry[ad] OR Limerick[ad] OR Tipperary[ad] OR Clare[ad] OR Galway[ad] OR Mayo[ad] OR Roscommon[ad] OR Sligo[ad] OR Leitrim[ad] OR Donegal[ad] OR Drogheda[ad] OR Dundalk[ad] OR Swords[ad] OR Bray[ad] OR Navan[ad] OR Munster[ad] OR Leinster[ad] OR Connacht[ad])) OR (((Hungar*[tw] OR Budapest[tw] OR Transdanubia[tw] OR Magyarorszag[tw] OR magyar[tw] OR Dunantuli[tw] OR Dunantul[tw] OR "Great Plain"[tw] OR "Alfold es eszak"[tw] OR "Eszak Alfold"[tw] OR "Del Alfold"[tw] OR Bacs[tw] OR Kiskun[tw] OR "Northen Alfold"[tw] OR "Sourthen Alfold"[tw] OR Baranya[tw] OR Bekes[tw] OR borsod[tw] OR Abauj[tw] OR Zemplen[tw] OR Fovaros[tw] OR Csongrad[tw] OR Fejer[tw] OR gyor[tw] OR moson[tw] OR sopron[tw] OR hajdu[tw] OR bihar[tw] OR Heves[tw] OR "jasz nagykun szolnok"[tw] OR komarom[tw] OR esztergom[tw] OR Nograd[tw] OR Pest[tw] OR Somogy[tw] OR szabolcs[tw] OR szatmar[tw] OR bereg[tw] OR Tolna[tw] OR Vas[tw] OR Veszprem[tw] OR Zala[tw] OR Zalaegerszeg[tw] OR Debrecen[tw] OR Miskolc[tw] OR Szeged[tw] OR Pecs[tw] OR Gyor[tw] OR Nyiregyhaza[tw] OR Kecskemet[tw] OR Szekesfehervar[tw] OR Szombathely[tw] OR Bekescsaba[tw] OR Eger[tw] OR Tatabanya[tw] OR Salgotarjan[tw] OR Kaposvar[tw] OR Szekszard[tw])) OR (Hungar*[ad] OR Budapest[ad] OR Transdanubia[ad] OR Magyarorszag[ad] OR magyar[ad] OR Dunantuli[ad] OR Dunantul[ad] OR "Great Plain"[ad] OR "Alfold es eszak"[ad] OR "Eszak Alfold"[ad] OR "Del Alfold"[ad] OR Bacs[ad] OR Kiskun[ad] OR "Northen Alfold"[ad] OR "Sourthen Alfold"[ad] OR Baranya[ad] OR Bekes[ad] OR borsod[ad] OR Abauj[ad] OR Zemplen[ad] OR Fovaros[ad] OR Csongrad[ad] OR Fejer[ad] OR gyor[ad] OR moson[ad] OR sopron[ad] OR hajdu[ad] OR bihar[ad] OR Heves[ad] OR "jasz nagykun szolnok"[ad] OR komarom[ad] OR esztergom[ad] OR Nograd[ad] OR Pest[ad] OR Somogy[ad] OR szabolcs[ad] OR szatmar[ad] OR bereg[ad] OR Tolna[ad] OR Vas[ad] OR Veszprem[ad] OR Zala[ad] OR Zalaegerszeg[ad] OR Debrecen[ad] OR Miskolc[ad] OR Szeged[ad] OR Pecs[ad] OR Gyor[ad] OR Nyiregyhaza[ad] OR Kecskemet[ad] OR Szekesfehervar[ad] OR Szombathely[ad] OR Bekescsaba[ad] OR Eger[ad] OR Tatabanya[ad] OR Salgotarjan[ad] OR Kaposvar[ad] OR Szekszard[ad])) OR (((Greece[ad] OR "Hellenic republic"[ad] OR Greek*[ad] OR Ellada[ad] OR "Elliniki Dimokratia"[ad] OR Hellas[ad] OR Hellenes[ad] OR Attica[ad] OR Attiki[ad] OR Makedonia[ad] OR Macedonia[ad] OR Thraki[ad] OR Thrace[ad] OR Crete[ad] OR Kriti[ad] OR Epirus[ad] OR Ipeiros[ad] OR "Ionia Nisia"[ad] OR "Ionion neson"[ad] OR "Ionian islands"[ad] OR "North aegean"[ad] OR "Aegean islands"[ad] OR "Nisoi Agaiou"[ad] OR "Notio Aigaio"[ad] OR Peloponnese[ad] OR Peloponnisos[ad] OR "Voreio Aigaio"[ad] OR "South aegean"[ad] OR Thessaly[ad] OR Thessalia[ad] OR Cycklades[ad] OR Kiklades[ad] OR Dodecanese[ad] OR Dodekanisa[ad] OR "Mount athos"[ad] OR "Omicronros Alphathos"[ad] OR Athens[ad] OR Athina[ad] OR Thessaloniki[ad] OR Thessalonica[ad] OR Patras[ad] OR Patra[ad] OR Heraklion[ad] OR Heraclion[ad] OR Iraklion[ad] OR Irakleion[ad] OR Iraklio[ad] OR Larissa[ad] OR Larisa[ad] OR Volos[ad] OR Rhodes[ad] OR Rodos[ad] OR Ioannina[ad] OR Janina[ad] OR Yannena[ad] OR Chania[ad] OR Chalcis[ad] OR Chalkida[ad] OR Alexandroupoli[ad])) OR (Greece[tw] OR "Hellenic republic"[tw] OR Greek*[tw] OR Ellada[tw] OR "Elliniki Dimokratia"[tw] OR Hellas[tw] OR Hellenes[tw] OR Attica[tw] OR Attiki[tw] OR Makedonia[tw] OR Macedonia[tw] OR Thraki[tw] OR Thrace[tw] OR Crete[tw] OR Kriti[tw] OR Epirus[tw] OR Ipeiros[tw] OR "Ionia Nisia"[tw] OR "Ionion neson"[tw] OR "Ionian islands"[tw] OR "North aegean"[tw] OR "Aegean islands"[tw] OR "Nisoi Agaiou"[tw] OR "Notio Aigaio"[tw] OR Peloponnese[tw] OR Peloponnisos[tw] OR "Voreio Aigaio"[tw] OR "South aegean"[tw] OR Thessaly[tw] OR Thessalia[tw] OR Cycklades[tw] OR Kiklades[tw] OR Dodecanese[tw] OR Dodekanisa[tw] OR "Mount athos"[tw] OR "Omicronros Alphathos"[tw] OR Athens[tw] OR Athina[tw] OR Thessaloniki[tw] OR Thessalonica[tw] OR Patras[tw] OR Patra[tw] OR Heraklion[tw] OR Heraclion[tw] OR Iraklion[tw] OR Irakleion[tw] OR Iraklio[tw] OR Larissa[tw] OR Larisa[tw] OR Volos[tw] OR Rhodes[tw] OR Rodos[tw] OR Ioannina[tw] OR Janina[tw] OR Yannena[tw] OR Chania[tw] OR Chalcis[tw] OR Chalkida[tw] OR Alexandroupoli[tw])) OR (((German*[ad] OR Deutschland[ad] OR Deutsch*[ad] OR Bundesrepublik[ad] OR Westdeutschland[ad] OR Ostdeutschland[ad] OR Baden[ad] OR Wuerttemberg[ad] OR Wurttemberg[ad] OR Bayern[ad] OR Bavaria[ad] OR Berlin[ad] OR Brandenburg[ad] OR Bremen[ad] OR Hamburg[ad] OR Hessen[ad] OR Hesse[ad] OR Hessia[ad] OR Mecklenburg[ad] OR Vorpommern[ad] OR Pomerania[ad] OR Niedersachsen[ad] OR Neddersassen[ad] OR Saxony[ad] OR Niederbayern[ad] OR "North Rhine"[ad] OR Westphalia[ad] OR Westfalen[ad] OR "Rhineland Palatinate"[ad] OR "Rheinland Pfalz"[ad] OR Saarland[ad] OR Sachsen[ad] OR "Schleswig Holstein"[ad] OR Thuringia[ad] OR Thuringen[ad] OR Thueringen[ad] OR Freiburg[ad] OR Karlsruhe[ad] OR Calsruhe[ad] OR Stuttgart[ad] OR Tubingen[ad] OR Oberbayern[ad] OR "Upper palatinate"[ad] OR Oberpfalz[ad] OR Franken[ad] OR Franconia[ad] OR Oberfranken[ad] OR Mittelfranken[ad] OR Schwaben[ad] OR Unterfranken[ad] OR Swabia[ad] OR Darmstadt[ad] OR Giessen[ad] OR Giessen[ad] OR Kassel[ad] OR Arnsberg[ad] OR Cologne[ad] OR Koln[ad] OR Koeln[ad] OR Detmold[ad] OR Dusseldorf[ad] OR Duesseldorf[ad] OR Munster[ad] OR Muenster[ad] OR Munich[ad] OR Munchen[ad] OR Muenchen[ad] OR Frankfurt[ad] OR Dortmund[ad] OR Essen[ad] OR Nurnberg[ad] OR Nuernberg[ad] OR Nuremberg[ad] OR Hanover[ad] OR Hannover[ad] OR Leipzig[ad] OR Dresden[ad] OR Ruhrgebiet[ad] OR Revier[ad] OR Ruhrpott[ad] OR Pott[ad] OR Ruhr[ad])) OR (German*[tw] OR Deutschland[tw] OR Deutsch*[tw] OR Bundesrepublik[tw] OR Westdeutschland[tw] OR Ostdeutschland[tw] OR Baden[tw] OR Wuerttemberg[tw] OR Wurttemberg[tw] OR Bayern[tw] OR Bavaria[tw] OR Berlin[tw] OR Brandenburg[tw] OR Bremen[tw] OR Hamburg[tw] OR Hessen[tw] OR Hesse[tw] OR Hessia[tw] OR Mecklenburg[tw] OR Vorpommern[tw] OR Pomerania[tw] OR Niedersachsen[tw] OR Neddersassen[tw] OR Saxony[tw] OR Niederbayern[tw] OR "North Rhine"[tw] OR Westphalia[tw] OR Westfalen[tw] OR "Rhineland Palatinate"[tw] OR "Rheinland Pfalz"[tw] OR Saarland[tw] OR Sachsen[tw] OR "Schleswig Holstein"[tw] OR Thuringia[tw] OR Thuringen[tw] OR Thueringen[tw] OR Freiburg[tw] OR Karlsruhe[tw] OR Calsruhe[tw] OR Stuttgart[tw] OR Tubingen[tw] OR Oberbayern[tw] OR "Upper palatinate"[tw] OR Oberpfalz[tw] OR Franken[tw] OR Franconia[tw] OR Oberfranken[tw] OR Mittelfranken[tw] OR Schwaben[tw] OR Unterfranken[tw] OR Swabia[tw] OR Darmstadt[tw] OR Giessen[tw] OR Giessen[tw] OR Kassel[tw] OR Arnsberg[tw] OR Cologne[tw] OR Koln[tw] OR Koeln[tw] OR Detmold[tw] OR Dusseldorf[tw] OR Duesseldorf[tw] OR Munster[tw] OR Muenster[tw] OR Munich[tw] OR Munchen[tw] OR Muenchen[tw] OR Frankfurt[tw] OR Dortmund[tw] OR Essen[tw] OR Nurnberg[tw] OR Nuernberg[tw] OR Nuremberg[tw] OR Hanover[tw] OR Hannover[tw] OR Leipzig[tw] OR Dresden[tw] OR Ruhrgebiet[tw] OR Revier[tw] OR Ruhrpott[tw] OR Pott[tw] OR Ruhr[tw])) OR (((France[ad] OR French*[ad] OR Francais[ad] OR Alsace[ad] OR Aquitaine[ad] OR Auvergne[ad] OR Brittany[ad] OR Bretagne[ad] OR Bourgogne[ad] OR Burgundy[ad] OR "Champagne Ardenne"[ad] OR "Franche Comte"[ad] OR "Ile de France"[ad] OR "Languedoc Roussillon"[ad] OR Limousin[ad] OR Lorraine[ad] OR Normandie[ad] OR Normandy[ad] OR "Midi Pyrenees"[ad] OR "Nord Pas de Calais"[ad] OR Loire[ad] OR Picardie[ad] OR Picardy[ad] OR "Poitou Charentes"[ad] OR Provence[ad] OR "Rhone Alpes"[ad] OR Corse[ad] OR Corsica[ad] OR Guiana[ad] OR Guyane[ad] OR Guadeloupe[ad] OR Martinique[ad] OR Reunion[ad] OR Mayotte[ad] OR Ain[ad] OR Aisne[ad] OR Allier[ad] OR "Alpes de Haute Provence"[ad] OR "Haute Alpes"[ad] OR "Alpes Maritimes"[ad] OR Ardeche[ad] OR Ardennes[ad] OR Ariege[ad] OR Aube[ad] OR Aude[ad] OR Aveyron[ad] OR "Bas Rhin"[ad] OR "Bouches du Rhone"[ad] OR Calvados[ad] OR Cantal[ad] OR Charente[ad] OR Cher[ad] OR Correze[ad] OR "Corse du Sud"[ad] OR "Cote d Or"[ad] OR "Cotes d Armor"[ad] OR "Cote d Azur"[ad] OR Creuse[ad] OR "Deux Sevres"[ad] OR Dordogne[ad] OR Doubs[ad] OR Drome[ad] OR Essonne[ad] OR Eure[ad] OR Finistere[ad] OR Gard[ad] OR Gers[ad] OR Gironde[ad] OR "Haute Corse"[ad] OR "Haute Garonne"[ad] OR "Haute Marne"[ad] OR "Hautes Alpes"[ad] OR "Haute Saone"[ad] OR "Haute Savoie"[ad] OR "Hautes Pyrenees"[ad] OR "Haute Vienne"[ad] OR "Haut Rhin"[ad] OR "Hauts de Seine"[ad] OR Herault[ad] OR "Ille et Vilaine"[ad] OR Indre[ad] OR Isere[ad] OR Jura[ad] OR Landes[ad] OR Loire[ad] OR Loiret[ad] OR (Lot[ad] AND (departement[ad] OR department[ad])) OR "Lot et Garonne"[ad] OR "Loir et Cher"[ad] OR Lozere[ad] OR Manche[ad] OR Marne[ad] OR Mayenne[ad] OR "Meurthe et Moselle"[ad] OR Meuse[ad] OR Morbihan[ad] OR Moselle[ad] OR (Nord[ad] AND (department[ad] OR departement[ad])) OR Nievre[ad] OR Oise[ad] OR Orne[ad] OR "Pas de calais"[ad] OR Paris[ad] OR "Puy de dome"[ad] OR "Pyrenees Atlantiques"[ad] OR "Pyrenees Orientales"[ad] OR Rhone[ad] OR Sarthe[ad] OR Savoie[ad] OR "Seine et Marne"[ad] OR "Seine Maritime"[ad] OR Somme[ad] OR Tarn[ad] OR "Territoire de Belfort"[ad] OR "Val de Marne"[ad] OR "Val d Oise"[ad] OR Var[ad] OR Vaucluse[ad] OR Vendee[ad] OR Vienne[ad] OR Vosges[ad] OR Yonne[ad] OR Yvelines[ad] OR Marseille[ad] OR Lyon[ad] OR Nice[ad] OR Nantes[ad] OR Strasbourg[ad] OR Montpellier[ad] OR Bordeaux[ad] OR Lille[ad] OR Toulouse[ad])) OR (France[tw] OR French*[tw] OR Francais[tw] OR Alsace[tw] OR Aquitaine[tw] OR Auvergne[tw] OR Brittany[tw] OR Bretagne[tw] OR Bourgogne[tw] OR Burgundy[tw] OR "Champagne Ardenne"[tw] OR "Franche Comte"[tw] OR "Ile de France"[tw] OR "Languedoc Roussillon"[tw] OR Limousin[tw] OR Lorraine[tw] OR Normandie[tw] OR Normandy[tw] OR "Midi Pyrenees"[tw] OR "Nord Pas de Calais"[tw] OR Loire[tw] OR Picardie[tw] OR Picardy[tw] OR "Poitou Charentes"[tw] OR Provence[tw] OR "Rhone Alpes"[tw] OR Corse[tw] OR Corsica[tw] OR Guiana[tw] OR Guyane[tw] OR Guadeloupe[tw] OR Martinique[tw] OR Reunion[tw] OR Mayotte[tw] OR Ain[tw] OR Aisne[tw] OR Allier[tw] OR "Alpes de Haute Provence"[tw] OR "Haute Alpes"[tw] OR "Alpes Maritimes"[tw] OR Ardeche[tw] OR Ardennes[tw] OR Ariege[tw] OR Aube[tw] OR Aude[tw] OR Aveyron[tw] OR "Bas Rhin"[tw] OR "Bouches du Rhone"[tw] OR Calvados[tw] OR Cantal[tw] OR Charente[tw] OR Cher[tw] OR Correze[tw] OR "Corse du Sud"[tw] OR "Cote d Or"[tw] OR "Cotes d Armor"[tw] OR "Cote d Azur"[tw] OR Creuse[tw] OR "Deux Sevres"[tw] OR Dordogne[tw] OR Doubs[tw] OR Drome[tw] OR Essonne[tw] OR Eure[tw] OR Finistere[tw] OR Gard[tw] OR Gers[tw] OR Gironde[tw] OR "Haute Corse"[tw] OR "Haute Garonne"[tw] OR "Haute Marne"[tw] OR "Hautes Alpes"[tw] OR "Haute Saone"[tw] OR "Haute Savoie"[tw] OR "Hautes Pyrenees"[tw] OR "Haute Vienne"[tw] OR "Haut Rhin"[tw] OR "Hauts de Seine"[tw] OR Herault[tw] OR "Ille et Vilaine"[tw] OR Indre[tw] OR Isere[tw] OR Jura[tw] OR Landes[tw] OR Loire[tw] OR Loiret[tw] OR (Lot[tw] AND (departement[tw] OR department[tw])) OR "Lot et Garonne"[tw] OR "Loir et Cher"[tw] OR Lozere[tw] OR Manche[tw] OR Marne[tw] OR Mayenne[tw] OR "Meurthe et Moselle"[tw] OR Meuse[tw] OR Morbihan[tw] OR Moselle[tw] OR (Nord[tw] AND (department[tw] OR departement[tw])) OR Nievre[tw] OR Oise[tw] OR Orne[tw] OR "Pas de calais"[tw] OR Paris[tw] OR "Puy de dome"[tw] OR "Pyrenees Atlantiques"[tw] OR "Pyrenees Orientales"[tw] OR Rhone[tw] OR Sarthe[tw] OR Savoie[tw] OR "Seine et Marne"[tw] OR "Seine Maritime"[tw] OR Somme[tw] OR Tarn[tw] OR "Territoire de Belfort"[tw] OR "Val de Marne"[tw] OR "Val d Oise"[tw] OR Var[tw] OR Vaucluse[tw] OR Vendee[tw] OR Vienne[tw] OR Vosges[tw] OR Yonne[tw] OR Yvelines[tw] OR Marseille[tw] OR Lyon[tw] OR Nice[tw] OR Nantes[tw] OR Strasbourg[tw] OR Montpellier[tw] OR Bordeaux[tw] OR Lille[tw] OR Toulouse[tw])) OR (((Finland[tw] OR Finnish*[tw] OR Suomi*[tw] OR Lapland[tw] OR Lappi[tw] OR Lappland[tw] OR Ostrobothnia[tw] OR Pohjanmaa[tw] OR Osterbotten[tw] OR Kainuu[tw] OR Kajanaland*[tw] OR Karelia[tw] OR Karjala[tw] OR Karelen[tw] OR Savonia[tw] OR Savo[tw] OR Savolax[tw] OR Pirkanmaa[tw] OR Birkaland[tw] OR Satakunta[tw] OR Satakunda[tw] OR Tavastia[tw] OR Tavastland[tw] OR "Paijat Hame"[tw] OR "Kanta Hame"[tw] OR Uusimaa[tw] OR Nyland[tw] OR Kymenlaakso[tw] OR Kymmenedalen[tw] OR Aland[tw] OR Ahvenanmaa[tw] OR Helsinki[tw] OR Helsingfors[tw] OR Espoo[tw] OR Esbo[tw] OR Tampere[tw] OR Tammerfors[tw] OR Vantaa[tw] OR Vanda[tw] OR Oulu[tw] OR Uleaborg[tw] OR Turku[tw] OR Abo[tw] OR Jyvaskyla[tw] OR Kuopio[tw] OR Lathi[tw] OR Lahtis[tw] OR Kouvola[tw])) OR (Finland[ad] OR Finnish*[ad] OR Suomi*[ad] OR Lapland[ad] OR Lappi[ad] OR Lappland[ad] OR Ostrobothnia[ad] OR Pohjanmaa[ad] OR Osterbotten[ad] OR Kainuu[ad] OR Kajanaland*[ad] OR Karelia[ad] OR Karjala[ad] OR Karelen[ad] OR Savonia[ad] OR Savo[ad] OR Savolax[ad] OR Pirkanmaa[ad] OR Birkaland[ad] OR Satakunta[ad] OR Satakunda[ad] OR Tavastia[ad] OR Tavastland[ad] OR "Paijat Hame"[ad] OR "Kanta Hame"[ad] OR Uusimaa[ad] OR Nyland[ad] OR Kymenlaakso[ad] OR Kymmenedalen[ad] OR Aland[ad] OR Ahvenanmaa[ad] OR Helsinki[ad] OR Helsingfors[ad] OR Espoo[ad] OR Esbo[ad] OR Tampere[ad] OR Tammerfors[ad] OR Vantaa[ad] OR Vanda[ad] OR Oulu[ad] OR Uleaborg[ad] OR Turku[ad] OR Abo[ad] OR Jyvaskyla[ad] OR Kuopio[ad] OR Lathi[ad] OR Lahtis[ad] OR Kouvola[ad])) OR (((Estonia*[tw] OR Eesti[tw] OR Esti[tw] OR Tallinn[tw] OR Harju[tw] OR Harjumaa[tw] OR Hiiu[tw] OR Hiiumaa[tw] OR "Ida Viru"[tw] OR "Ida Virumaa"[tw] OR Jarvamaa[tw] OR Jarva[tw] OR Jogevamaa[tw] OR Jogeva[tw] OR Laanemma[tw] OR Laane[tw] OR Parnu[tw] OR Parnumaa[tw] OR Polva[tw] OR Polvamaa[tw] OR Rapla[tw] OR Raplamaa[tw] OR Saare[tw] OR Saaremaa[tw] OR Tartu[tw] OR Tartumaa[tw] OR Valga[tw] OR Valgamaa[tw] OR Valgamaakond[tw] OR Viljandimaa[tw] OR Viljandi[tw] OR Voru[tw] OR Vorumaa[tw] OR Narva[tw] OR Parnu[tw] OR "Kohtla Jarve"[tw] OR Viljandi[tw] OR Rakvere[tw] OR Maardu[tw] OR Sillamae[tw] OR Kuressaare[tw])) OR (Estonia*[ad] OR Eesti[ad] OR Esti[ad] OR Tallinn[ad] OR Harju[ad] OR Harjumaa[ad] OR Hiiu[ad] OR Hiiumaa[ad] OR "Ida Viru"[ad] OR "Ida Virumaa"[ad] OR Jarvamaa[ad] OR Jarva[ad] OR Jogevamaa[ad] OR Jogeva[ad] OR Laanemma[ad] OR Laane[ad] OR Parnu[ad] OR Parnumaa[ad] OR Polva[ad] OR Polvamaa[ad] OR Rapla[ad] OR Raplamaa[ad] OR Saare[ad] OR Saaremaa[ad] OR Tartu[ad] OR Tartumaa[ad] OR Valga[ad] OR Valgamaa[ad] OR Valgamaakond[ad] OR Viljandimaa[ad] OR Viljandi[ad] OR Voru[ad] OR Vorumaa[ad] OR Narva[ad] OR Parnu[ad] OR "Kohtla Jarve"[ad] OR Viljandi[ad] OR Rakvere[ad] OR Maardu[ad] OR Sillamae[ad] OR Kuressaare[ad]))) OR ((((Denmark[tw] OR Danish*[tw] OR Danmark[tw] OR dansk*[tw] OR Hovedstaden[tw] OR Midtjylland[tw] OR Nordjylland[tw] OR Sjaelland[tw] OR Sealand[tw] OR Syddanmark[tw] OR Jutland[tw] OR Jylland[tw] OR Nordjylland[tw] OR Sonderjyllands[tw] OR "Zealand region"[tw] OR "region Zealand"[tw] OR Hillerod[tw] OR Viborg[tw] OR Aalborg[tw] OR Alborg[tw] OR Soro[tw] OR Vejle[tw] OR Copenhagen[tw] OR Kobenhavn[tw] OR Arhus[tw] OR Aarhus[tw] OR Roskilde[tw] OR Odense[tw] OR Frederiksberg[tw] OR Esbjerg[tw] OR Gentofte[tw] OR Gladsaxe[tw] OR Randers[tw] OR Kolding[tw])) OR (Denmark[ad] OR Danish*[ad] OR Danmark[ad] OR dansk*[ad] OR Hovedstaden[ad] OR Midtjylland[ad] OR Nordjylland[ad] OR Sjaelland[ad] OR Sealand[ad] OR Syddanmark[ad] OR Jutland[ad] OR Jylland[ad] OR Nordjylland[ad] OR Sonderjyllands[ad] OR "Zealand region"[ad] OR "region Zealand"[ad] OR Hillerod[ad] OR Viborg[ad] OR Aalborg[ad] OR Alborg[ad] OR Soro[ad] OR Vejle[ad] OR Copenhagen[ad] OR Kobenhavn[ad] OR Arhus[ad] OR Aarhus[ad] OR Roskilde[ad] OR Odense[ad] OR Frederiksberg[ad] OR Esbjerg[ad] OR Gentofte[ad] OR Gladsaxe[ad] OR Randers[ad] OR Kolding[ad])) OR ((Czech*[tw] OR Cesk*[tw] OR Stredoces*[tw] OR Jihoces*[tw] OR Bohemia[tw] OR "Bohemian region"[tw] OR Kralovehradec*[tw] OR "Hradec Kralove"[tw] OR Karlovars*[tw] OR "Karlovy Vary"[tw] OR Liberec*[tw] OR Moravskoslezs*[tw] OR "Moravian Silesian"[tw] OR Olomouc*[tw] OR Pardubic*[tw] OR Plzen*[tw] OR Pilsen[tw] OR Prage[tw] OR Praha[tw] OR Prag[tw] OR Jihomorav*[tw] OR Moravia[tw] OR Moravian[tw] OR Morava[tw] OR Usteck*[tw] OR Usti[tw] OR Vysocina[tw] OR Zlin[tw] OR Zlinsk*[tw] OR "Ceske Budejovice"[tw] OR Budweis[tw] OR Brno[tw] OR Ostrava[tw])) OR (Czech*[ad] OR Cesk*[ad] OR Stredoces*[ad] OR Jihoce*[ad] OR Bohemia[ad] OR "Bohemian region"[ad] OR Kralovehradec*[ad] OR "Hradec Kralove"[ad] OR Karlovars*[ad] OR "Karlovy Vary"[ad] OR Liberec*[ad] OR Moravskoslezsk*[ad] OR "Moravian Silesian"[ad] OR Olomouc*[ad] OR Pardubic*[ad] OR Pardubice[ad] OR Plzen*[ad] OR Pilsen[ad] OR Prage[ad] OR Praha[ad] OR Prag[ad] OR Jihomorav*[ad] OR Moravia[ad] OR Moravian[ad] OR Morava[ad] OR Usteck*[ad] OR Usti[ad] OR Vysocina[ad] OR Zlin[ad] OR Zlinsk*[ad] OR "Ceske Budejovice"[ad] OR Budweis[ad] OR Brno[ad] OR Ostrava[ad]) OR (((Cyprus[tw] OR Cypriot*[tw] OR Kypros[tw] OR Kibris[tw] OR kypriaki*[tw] OR Nicosia[tw] OR Lefkosa[tw] OR Lefkosia[tw] OR Famagusta[tw] OR Magusa[tw] OR Ammochostos[tw] OR Gazimagusa[tw] OR Kyrenia[tw] OR Girne[tw] OR Keryneia[tw] OR Larnaca[tw] OR Larnaka[tw] OR Limassol[tw] OR Lemesos[tw] OR Limasol[tw] OR Leymosun[tw] OR Paphos[tw] OR Pafos[tw] OR Baf[tw] OR Gazibaf[tw] OR Protaras[tw] OR Pergamos[tw] OR Beyarmudu[tw] OR Morfou[tw] OR Guzelyurt[tw] OR Omorfo[tw] OR Morphou[tw] OR Aradippou[tw])) OR (Cyprus[ad] OR Cypriot*[ad] OR Kypros[ad] OR Kibris[ad] OR kypriaki*[ad] OR Nicosia[ad] OR Lefkosa[ad] OR Lefkosia[ad] OR Famagusta[ad] OR Magusa[ad] OR Ammochostos[ad] OR Gazimagusa[ad] OR Kyrenia[ad] OR Girne[ad] OR Keryneia[ad] OR Larnaca[ad] OR Larnaka[ad] OR Limassol[ad] OR Lemesos[ad] OR Limasol[ad] OR Leymosun[ad] OR Paphos[ad] OR Pafos[ad] OR Baf[ad] OR Gazibaf[ad] OR Protaras[ad] OR Pergamos[ad] OR Beyarmudu[ad] OR Morfou[ad] OR Guzelyurt[ad] OR Omorfo[ad] OR Morphou[ad] OR Aradippou[ad])) OR (((Croatia*[tw] OR Hrvatsk*[tw] OR hrvat[tw] OR Bjelovar[tw] OR "Bjelovarsko bilogorska"[tw] OR "Brod Posavina"[tw] OR "Brodsko posavska"[tw] OR "Dubrovnik Neretva"[tw] OR "dubrovacko neretvanska"[tw] OR Istria[tw] OR Istarska[tw] OR Karlovacka[tw] OR Karlovac[tw] OR "Koprivnicko krizevacka"[tw] OR Koprivnica[tw] OR Krizevci[tw] OR "Krapina Zagorje"[tw] OR "Krapinsko zagorska"[tw] OR "Lika Senj"[tw] OR "Licko senjska"[tw] OR Medimurska[tw] OR Medimurje[tw] OR Osijek[tw] OR Osjecko[tw] OR Baranja[tw] OR "Osjecko baranjska"[tw] OR "Pozega Slavonia"[tw] OR "Pozesko slavonska"[tw] OR "Primorje Gorski Kotar"[tw] OR "Primorsko goranska"[tw] OR "Sibensko kninska"[tw] OR "Sibensko kninske"[tw] OR Sibenik[tw] OR Knin[tw] OR Sisak[tw] OR "Sisacko moslavacka"[tw] OR Moslavina[tw] OR "Splitsko dalmatinska"[tw] OR Split[tw] OR Dalmatia[tw] OR Varazdin[tw] OR Varazdinska[tw] OR "Viroviticko-podravska"[tw] OR Virovitica[tw] OR Podravina[tw] OR "Vukovarsko srijemska"[tw] OR Vukovar[tw] OR Srijem[tw] OR Zadar[tw] OR Zadarska[tw] OR Zagreb[tw] OR Zagrebacka[tw] OR Rijeka[tw] OR "Velika gorica"[tw] OR "Slavonski brod"[tw] OR Pula[tw])) OR (Croatia*[ad] OR Hrvatsk*[ad] OR hrvat[ad] OR Bjelovar[ad] OR "Bjelovarsko bilogorska"[ad] OR "Brod Posavina"[ad] OR "Brodsko posavska"[ad] OR "Dubrovnik Neretva"[ad] OR "dubrovacko neretvanska"[ad] OR Istria[ad] OR Istarska[ad] OR Karlovacka[ad] OR Karlovac[ad] OR "Koprivnicko krizevacka"[ad] OR Koprivnica[ad] OR Krizevci[ad] OR "Krapina Zagorje"[ad] OR "Krapinsko zagorska"[ad] OR "Lika Senj"[ad] OR "Licko senjska"[ad] OR Medimurska[ad] OR Medimurje[ad] OR Osijek[ad] OR Osjecko[ad] OR Baranja[ad] OR "Osjecko baranjska"[ad] OR "Pozega Slavonia"[ad] OR "Pozesko slavonska"[ad] OR "Primorje Gorski Kotar"[ad] OR "Primorsko goranska"[ad] OR "Sibensko kninska"[ad] OR "Sibensko kninske"[ad] OR Sibenik[ad] OR Knin[ad] OR Sisak[ad] OR "Sisacko moslavacka"[ad] OR Moslavina[ad] OR "Splitsko dalmatinska"[ad] OR Split[ad] OR Dalmatia[ad] OR Varazdin[ad] OR Varazdinska[ad] OR "Viroviticko-podravska"[ad] OR Virovitica[ad] OR Podravina[ad] OR "Vukovarsko srijemska"[ad] OR Vukovar[ad] OR Srijem[ad] OR Zadar[ad] OR Zadarska[ad] OR Zagreb[ad] OR Zagrebacka[ad] OR Rijeka[ad] OR "Velika gorica"[ad] OR "Slavonski brod"[ad] OR Pula[ad])) OR (((Bulgaria*[tw] OR Sofia[tw] OR Gabrovo[tw] OR Blagoevgrad[tw] OR "Pirin Macedonia"[tw] OR Burgas[tw] OR Dobrich[tw] OR Haskovo[tw] OR Kardzhali[tw] OR Kurdzhali[tw] OR Kyustendil[tw] OR Lovech[tw] OR Montana[tw] OR Pazardzhik[tw] OR Pernik[tw] OR Pleven[tw] OR Plovdiv[tw] OR Razgrad[tw] OR Rousse[tw] OR Ruse[tw] OR Shumen[tw] OR Sliven[tw] OR Silistra[tw] OR Smolyan[tw] OR "Stara Zagora"[tw] OR Targovishte[tw] OR Varna[tw] OR Tarnovo[tw] OR Vidin[tw] OR Vratsa[tw] OR Vratza[tw] OR Yambol[tw])) OR (Bulgaria*[ad] OR Sofia[ad] OR Gabrovo[ad] OR Blagoevgrad[ad] OR "Pirin Macedonia"[ad] OR Burgas[ad] OR Dobrich[ad] OR Haskovo[ad] OR Kardzhali[ad] OR Kurdzhali[ad] OR Kyustendil[ad] OR Lovech[ad] OR Montana[ad] OR Pazardzhik[ad] OR Pernik[ad] OR Pleven[ad] OR Plovdiv[ad] OR Razgrad[ad] OR Rousse[ad] OR Ruse[ad] OR Shumen[ad] OR Sliven[ad] OR Silistra[ad] OR Smolyan[ad] OR "Stara Zagora"[ad] OR Targovishte[ad] OR Varna[ad] OR Tarnovo[ad] OR Vidin[ad] OR Vratsa[ad] OR Vratza[ad] OR Yambol[ad])) OR (((Belgi*[tw] OR Belge[tw] OR Belgisch[tw] OR Brussel*[tw] OR Bruxelles[tw] OR Bruxelloise[tw] OR Flemish[tw] OR Flamand[tw] OR Flemisch[tw] OR Flanders[tw] OR Flandern[tw] OR Flandre[tw] OR Vlaanderen[tw] OR Vlaams[tw] OR Flamande[tw] OR Waals[tw] OR Walloon*[tw] OR Wallon*[tw] OR Antwerp*[tw] OR Anvers[tw] OR Ostflandern[tw] OR "Vlaams Brabant"[tw] OR Limbourg[tw] OR Limburg[tw] OR Hainault[tw] OR Hainaut[tw] OR Henegouwen[tw] OR Hennegau[tw] OR Liege[tw] OR Luik[tw] OR Luttich[tw] OR Namur[tw] OR Namen[tw] OR Westflandern[tw] OR "Waals Brabant"[tw] OR Ghent[tw] OR Gent[tw] OR Gand[tw] OR Charleroi[tw] OR Bruges[tw] OR Brugge[tw] OR Schaerbeek[tw] OR Schaarbeek[tw] OR Anderlecht[tw] OR Leuven[tw] OR Louvain[tw] OR Hasselt[tw] OR Mons[tw] OR Wavre[tw] OR Waver[tw])) OR (Belgi*[ad] OR Belge[ad] OR Belgisch[ad] OR Brussel*[ad] OR Bruxelles[ad] OR Bruxelloise[ad] OR Flemish[ad] OR Flamand[ad] OR Flemisch[ad] OR Flanders[ad] OR Flandern[ad] OR Flandre[ad] OR Vlaanderen[ad] OR Vlaams[ad] OR Flamande[ad] OR Waals[ad] OR Walloon*[ad] OR Wallon*[ad] OR Antwerp*[ad] OR Anvers[ad] OR Ostflandern[ad] OR "Vlaams Brabant"[ad] OR Limbourg[ad] OR Limburg[ad] OR Hainault[ad] OR Hainaut[ad] OR Henegouwen[ad] OR Hennegau[ad] OR Liege[ad] OR Luik[ad] OR Luttich[ad] OR Namur[ad] OR Namen[ad] OR Westflandern[ad] OR "Waals Brabant"[ad] OR Ghent[ad] OR Gent[ad] OR Gand[ad] OR Charleroi[ad] OR Bruges[ad] OR Brugge[ad] OR Schaerbeek[ad] OR Schaarbeek[ad] OR Anderlecht[ad] OR Leuven[ad] OR Louvain[ad] OR Hasselt[ad] OR Mons[ad] OR Wavre[ad] OR Waver[ad])) OR (((Austria*[tw] OR Vienna[tw] OR Wien[tw] OR Osterreich*[tw] OR Sudosterreich[tw] OR Westosterreich[tw] OR Niederosterreich[tw] OR Burgenland[tw] OR Carinthia[tw] OR Karinthia[tw] OR Karnten[tw] OR Oberosterreich[tw] OR Styria[tw] OR Steiermark[tw] OR Salzburg[tw] OR Saizburg[tw] OR Tyrol[tw] OR Tirol[tw] OR Becs[tw] OR Vorarlberg[tw] OR Bregenz[tw] OR Linz[tw] OR Eisenstadt[tw] OR Innsbruck[tw] OR Graz[tw] OR Klagenfurt[tw] OR Polten[tw] OR Villach[tw] OR Wels[tw] OR Dornbirn[tw] OR Feldkirch[tw] OR Steyr[tw])) OR (Austria*[ad] OR Vienna[ad] OR Wien[ad] OR Osterreich*[ad] OR Sudosterreich[ad] OR Westosterreich[ad] OR Niederosterreich[ad] OR Burgenland[ad] OR Carinthia[ad] OR Karnten[ad] OR Oberosterreich[ad] OR Styria[ad] OR Steiermark[ad] OR Salzburg[ad] OR Saizburg[ad] OR Tyrol[ad] OR Tirol[ad] OR Becs[ad] OR Vorarlberg[ad] OR Bregenz[ad] OR Linz[ad] OR Eisenstadt[ad] OR Innsbruck[ad] OR Graz[ad] OR Klagenfurt[ad] OR Polten[ad] OR Villach[ad] OR Wels[ad] OR Dornbirn[ad] OR Feldkirch[ad] OR Steyr[ad]))) OR (((((((((Iceland[tw] OR Icelandic*[tw] OR islenska*[tw] OR Icelander*[tw] OR islendinga*[tw] OR Reykjavik[tw] OR Reykjavikurborg[tw] OR Hofudborgarsvaedid[tw] OR Sudurnes[tw] OR Vesturland[tw] OR Vestfirdir[tw] OR Westfjords[tw] OR Nordurland[tw] OR Austurland[tw] OR Sudurland[tw] OR Kopavogur[tw] OR Hafnarfjordur[tw])) OR (Iceland[ad] OR Icelandic*[ad] OR islenska*[ad] OR Icelander*[ad] OR islendinga*[ad] OR Reykjavik[ad] OR Reykjavikurborg[ad] OR Hofudborgarsvaedid[ad] OR Sudurnes[ad] OR Vesturland[ad] OR Vestfirdir[ad] OR Westfjords[ad] OR Nordurland[ad] OR Austurland[ad] OR Sudurland[ad] OR Kopavogur[ad] OR Hafnarfjordur[ad])) OR (Switzerland[tw] OR Schweiz[tw] OR Schweizerische[tw] OR Swiss[tw] OR Suisse[tw] OR Aargau[tw] OR Argovia[tw] OR Ausserrhoden[tw] OR "Outer Rhodes"[tw] OR Innerrhoden[tw] OR "Inner Rhodes"[tw] OR Basel[tw] OR Bern[tw] OR Berne[tw] OR Fribourg[tw] OR Freiburg[tw] OR Geneva[tw] OR Geneve[tw] OR Glarus[tw] OR Graubunden[tw] OR Grisons[tw] OR Grigioni[tw] OR jura[tw] OR Lucerne[tw] OR Luzern[tw] OR Neuchatel[tw] OR Zurich[tw] OR (Uri[tw] AND (canton[tw] OR Kanton[tw])) OR Schwyz[tw] OR Obwalden[tw] OR Nidwalden[tw] OR Zug[tw] OR Solothurn[tw] OR Schaffhausen[tw] OR Thurgau[tw] OR Thurgovia[tw] OR Ticino[tw] OR Tessin[tw] OR Vaud[tw] OR Valais[tw] OR Wallis[tw] OR "St Gallen"[tw] OR Lausanne[tw] OR Winterthur[tw] OR Winterthour[tw] OR Lugano[tw] OR Biel[tw] OR Bienne[tw])) OR (Switzerland[ad] OR Schweiz[ad] OR Schweizerische[ad] OR Swiss[ad] OR Suisse[ad] OR Aargau[ad] OR Argovia[ad] OR Ausserrhoden[ad] OR "Outer Rhodes"[ad] OR Innerrhoden[ad] OR "Inner Rhodes"[ad] OR Basel[ad] OR Bern[ad] OR Berne[ad] OR Fribourg[ad] OR Freiburg[ad] OR Geneva[ad] OR Geneve[ad] OR Glarus[ad] OR Graubunden[ad] OR Grisons[ad] OR Grigioni[ad] OR jura[ad] OR Lucerne[ad] OR Luzern[ad] OR Neuchatel[ad] OR Zurich[ad] OR (Uri[ad] AND (canton[ad] OR Kanton[ad])) OR Schwyz[ad] OR Obwalden[ad] OR Nidwalden[ad] OR Zug[ad] OR Solothurn[ad] OR Schaffhausen[ad] OR Thurgau[ad] OR Thurgovia[ad] OR Ticino[ad] OR Tessin[ad] OR Vaud[ad] OR Valais[ad] OR Wallis[ad] OR "St Gallen"[ad] OR Lausanne[ad] OR Winterthur[ad] OR Winterthour[ad] OR Lugano[ad] OR Biel[ad] OR Bienne[ad])) OR (Norway[tw] OR Norwegian*[tw] OR Norge[tw] OR Noreg[tw] OR Norgga[tw] OR Ostfold[tw] OR Akershus[tw] OR Oslo[tw] OR Hedmark[tw] OR Oppland[tw] OR Buskerud[tw] OR Vestfold[tw] OR Telemark[tw] OR "Aust Agder"[tw] OR "Vest Agder"[tw] OR Rogaland[tw] OR Hordaland[tw] OR "Sogn og fjordane"[tw] OR "Sogn and fjordane"[tw] OR "sogn fjordane"[tw] OR "More og Romsdal"[tw] OR "More and Romsdal"[tw] OR "More Romsdal"[tw] OR Trondelag[tw] OR Nordland[tw] OR Troms[tw] OR Finnmark[tw] OR Bergen[tw] OR Stavanger[tw] OR Sandnes[tw] OR Trondheim[tw] OR Kristiansand[tw] OR Drammen[tw] OR Fredrikstad[tw] OR Sarpsborg[tw] OR Porsgrunn[tw] OR Skien[tw] OR Tonsberg[tw] OR Alesund[tw])) OR (Norway[ad] OR Norwegian*[ad] OR Norge[ad] OR Noreg[ad] OR Norgga[ad] OR Ostfold[ad] OR Akershus[ad] OR Oslo[ad] OR Hedmark[ad] OR Oppland[ad] OR Buskerud[ad] OR Vestfold[ad] OR Telemark[ad] OR "Aust Agder"[ad] OR "Vest Agder"[ad] OR Rogaland[ad] OR Hordaland[ad] OR "Sogn og fjordane"[ad] OR "Sogn and fjordane"[ad] OR "sogn fjordane"[ad] OR "More og Romsdal"[ad] OR "More and Romsdal"[ad] OR "More Romsdal"[ad] OR Trondelag[ad] OR Nordland[ad] OR Troms[ad] OR Finnmark[ad] OR Bergen[ad] OR Stavanger[ad] OR Sandnes[ad] OR Trondheim[ad] OR Kristiansand[ad] OR Drammen[ad] OR Fredrikstad[ad] OR Sarpsborg[ad] OR Porsgrunn[ad] OR Skien[ad] OR Tonsberg[ad] OR Alesund[ad])) OR (Liechtenstein[tw] OR Vaduz[tw] OR Triesenberg[tw] OR Triesen[tw] OR Schellenberg[tw] OR Schaan[tw] OR Ruggell[tw] OR Planken[tw] OR Mauren[tw] OR Gamprin[tw] OR Eschen[tw] OR Balzers[tw])) OR (Liechtenstein[ad] OR Vaduz[ad] OR Triesenberg[ad] OR Triesen[ad] OR Schellenberg[ad] OR Schaan[ad] OR Ruggell[ad] OR Planken[ad] OR Mauren[ad] OR Gamprin[ad] OR Eschen[ad] OR Balzers[ad])) OR ("European Union"[Mesh] OR "Europe"[MeSH] OR Europa[tw] OR Europe*[tw] OR Scandinavia*[tw] OR Scandinavia*[ad] OR Mediterranean[tw] OR "EEA countries"[tw] OR "EU country"[tw] OR "EU countries"[tw] OR Mediterranean[ad] OR Europe*[ad] OR Baltic[tw] OR Baltic[ad] OR Yugoslavia[tw] OR Jugoslavija[tw] OR Jugoslavija[AD] OR Yugoslavia[ad] OR "EU country"[tw] OR "Eu countries"[tw] OR global*[tw] OR world[tw] OR worldwide[tw])** |  |

## Embase search (Embase.com)

Date of the search: 16/03/2015

Language limit: no limits

Date limits: from 2005 to 2015

Number of results: 7801

| No. | Query | Results |
| --- | --- | --- |
| #9 | **#1** AND **#7** AND [2005-2015]/py | **7801** |
| #8 | **#1** AND **#7** | **12837** |
| #7 | **#4** OR **#5** OR **#6** | **24163** |
| #6 | (**epidemiolog*** NEAR/5 (**'hepatitis b'** OR **'hepatitis c'** OR **hepaciviru*** OR **'hbv'** OR **'hcv'** OR **hbsag** OR **'hbs ag'** OR **'australia antigen'** OR **'australia antigens'**)):ti | **1146** |
| #5 | (**prevalence** NEAR/5 (**'hepatitis b'** OR **'hepatitis c'** OR **hepaciviru*** OR **'hbv'** OR **'hcv'** OR **hbsag** OR **'hbs ag'** OR **'australia antigen'** OR **'australia antigens'**)):ab,ti | **10785** |
| #4 | **#2** AND **#3** | **20368** |
| #3 | **'hepatitis b'**/exp OR **'hepatitis c'**/exp OR **'hepatitis b virus'**/exp OR **'hepatitis c virus'**/exp OR **'hepatitis b'**:ab,ti OR **'hepatitis c'**:ab,ti OR **hepaciviru***:ab,ti OR **'hbv'**:ab,ti OR **'hcv'**:ab,ti OR **hbsag**:ab,ti OR **'hbs ag'**:ab,ti OR **'hepatitis b antibody'**/exp OR **'hepatitis b surface antigen'**/exp OR **'australia antigen'**:ab,ti OR **'australia antigens'**:ab,ti OR **'hepatitis c antigen'**/exp OR **'hepatitis c antibody'**/exp | **201216** |
| #2 | **'prevalence'**/exp OR **'seroepidemiology'**/exp OR **'disease surveillance'**/exp OR **'sero epidemiology'**:ab,ti OR **'sero epidemiological'**:ab,ti OR **'sero epidemiologic'**:ab,ti OR **seroepidemiolog***:ab,ti OR **surveillance**:ti OR **serolog***:ti OR **serosurvey***:ab,ti OR **prevalence***:ab,ti OR **'population surveillance'**:ab,ti | **513612** |
| #1 |  | **11070462** |
|  | \| #1.8 \| **#1.1** OR **#1.3** OR **#1.4** OR **#1.5** OR **#1.6** OR **#1.7** \| **11070462** \| \| --- \| --- \| --- \| \| #1.7 \| **'united kingdom'**:ad OR **britain**:ad OR **british**:ad OR (**england**:ad NOT **'new england'**:ad) OR **english**:ad OR **scotland**:ad OR **scottish**:ad OR **wales**:ad OR **welsh**:ad OR **'northen ireland'**:ad OR **london**:ad OR **'east midlands'**:ad OR **'west midlands'**:ad OR **yorkshire**:ad OR **'east anglia'**:ad OR **bedfordshire**:ad OR **hertfordshire**:ad OR **essex**:ad OR **peterborough**:ad OR **cambridgeshire**:ad OR **norfolk**:ad OR **suffolk**:ad OR **luton**:ad OR **bedford**:ad OR **'southend on sea'**:ad OR **thurrock**:ad OR **derbyshire**:ad OR **nottinghamshire**:ad OR **leicestershire**:ad OR **rutland**:ad OR **lincolnshire**:ad OR **derby**:ad OR **leicester**:ad OR **northamptonshire**:ad OR **nottingham**:ad OR **'tyne and wear'**:ad OR **'tees valley'**:ad OR **'durham'**:ad OR **darlington**:ad OR **hartlepool**:ad OR **'stockton on tees'**:ad OR **northumberland**:ad OR **teesside**:ad OR **sunderland**:ad OR **cumbria**:ad OR **cheshire**:ad OR **manchester**:ad OR **lancashire**:ad OR **merseyside**:ad OR (**blackburn**:ad AND **darwen**:ad) OR **blackpool**:ad OR **chester**:ad OR **liverpool**:ad OR **sefton**:ad OR **warrington**:ad OR **wirral**:ad OR **berkshire**:ad OR **buckinghamshire**:ad OR **oxfordshire**:ad OR **hampshire**:ad OR **'isle of wight'**:ad OR **kent**:ad OR **surrey**:ad OR **sussex**:ad OR (**brighton**:ad AND **hove**:ad) OR **'milton keynes'**:ad OR **portsmouth**:ad OR **southampton**:ad OR **devon**:ad OR **dorset**:ad OR **somerset**:ad OR **gloucestershire**:ad OR **wiltshire**:ad OR **bath**:ad OR **bournemouth**:ad OR **poole**:ad OR **bristol**:ad OR **plymouth**:ad OR **swindon**:ad OR **torbay**:ad OR **herefordshire**:ad OR **staffordshire**:ad OR **birmingham**:ad OR **coventry**:ad OR **dudley**:ad OR **sandwell**:ad OR **shropshire**:ad OR **solihull**:ad OR **'stoke on trent'**:ad OR **telford**:ad OR **wrekin**:ad OR **walsall**:ad OR **warwickshire**:ad OR **wolverhampton**:ad OR **worcestershire**:ad OR **barnsley**:ad OR **doncaster**:ad OR **rotherham**:ad OR **bradford**:ad OR **calderdale**:ad OR **kirklees**:ad OR **kingston**:ad OR **leeds**:ad OR **sheffield**:ad OR **wakefield**:ad OR (**york**:ad NOT **'new york'**:ad) OR **antrim**:ad OR **ards**:ad OR **armagh**:ad OR **ballymena**:ad OR **ballymoney**:ad OR **banbridge**:ad OR **carrickfergus**:ad OR **castlereagh**:ad OR **coleraine**:ad OR **cookstown**:ad OR **craigavon**:ad OR (**down**:ad AND (**district**:ad OR **council**:ad)) OR **dungannon**:ad OR **fermanagh**:ad OR **larne**:ad OR **limavady**:ad OR **lisburn**:ad OR **magherafelt**:ad OR **moyle**:ad OR (**newry**:ad AND **mourne**:ad) OR **newtownabbey**:ad OR **omagh**:ad OR **strabane**:ad OR **londonderry**:ad OR **tyrone**:ad OR **belfast**:ad OR **aberdeen**:ad OR **aberdeenshire**:ad OR **angus**:ad OR **dundee**:ad OR (**argyll**:ad AND **bute**:ad) OR **clackmannanshire**:ad OR **fife**:ad OR **ayrshire**:ad OR **dunbartonshire**:ad OR **lothian**:ad OR **renfrewshire**:ad OR **edinburgh**:ad OR **falkirk**:ad OR **glasgow**:ad OR **highland***:ad OR **inverclyde**:ad OR **midlothian**:ad OR **moray**:ad OR **lanarkshire**:ad OR (**perth**:ad AND **kinross**:ad) OR **stirling**:ad OR **'orkney islands'**:ad OR **'eileanan siar'**:ad OR **'shetland islands'**:ad OR **bridgend**:ad OR **'neath port talbot'**:ad OR **cardiff**:ad OR (**vale**:ad AND **glamorgan**:ad) OR **'central valleys'**:ad OR **conwy**:ad OR **denbighshire**:ad OR **flintshire**:ad OR **wrexham**:ad OR **'gwent valleys'**:ad OR **gwynedd**:ad OR (**isle**:ad AND **anglesey**:ad) OR **'monmouthshire'**:ad OR **'newport'**:ad OR **powys**:ad OR **swansea**:ad OR **ceredigion**:ad OR **carmarthenshire**:ad OR **pembrokeshire**:ad OR **'merthyr tydfil'**:ad OR **'rhondda cynon taff'**:ad OR **'blaenau gwent'**:ad OR **caerphilly**:ad OR **torfaen**:ad OR **caithness**:ad OR **'sutherland and ross'**:ad OR **cromarty**:ad OR **teeside**:ad OR **tyneside**:ad OR **wearside**:ad OR **'west mercia'**:ad OR **avon**:ad OR **ulster**:ad OR **derry**:ad OR **medway**:ad OR **'east riding'**:ad OR **'west riding'**:ad OR **'lake district'**:ad OR **'peak district'**:ad OR **cumberland**:ad OR **dartmoor**:ad OR **exmoor**:ad OR **'united kingdom'**:ab,ti OR **britain**:ab,ti OR **british**:ab,ti OR (**england**:ab,ti NOT **'new england'**:ab,ti) OR **english**:ab,ti OR **scotland**:ab,ti OR **scottish**:ab,ti OR **wales**:ab,ti OR **welsh**:ab,ti OR **'northen ireland'**:ab,ti OR **london**:ab,ti OR **'east midlands'**:ab,ti OR **'west midlands'**:ab,ti OR **yorkshire**:ab,ti OR **'east anglia'**:ab,ti OR **bedfordshire**:ab,ti OR **hertfordshire**:ab,ti OR **essex**:ab,ti OR **peterborough**:ab,ti OR **cambridgeshire**:ab,ti OR **norfolk**:ab,ti OR **suffolk**:ab,ti OR **luton**:ab,ti OR **bedford**:ab,ti OR **'southend on sea'**:ab,ti OR **thurrock**:ab,ti OR **derbyshire**:ab,ti OR **nottinghamshire**:ab,ti OR **leicestershire**:ab,ti OR **rutland**:ab,ti OR **lincolnshire**:ab,ti OR **derby**:ab,ti OR **leicester**:ab,ti OR **northamptonshire**:ab,ti OR **nottingham**:ab,ti OR **'tyne and wear'**:ab,ti OR **'tees valley'**:ab,ti OR **'durham'**:ab,ti OR **darlington**:ab,ti OR **hartlepool**:ab,ti OR **'stockton on tees'**:ab,ti OR **northumberland**:ab,ti OR **teesside**:ab,ti OR **sunderland**:ab,ti OR **cumbria**:ab,ti OR **cheshire**:ab,ti OR **manchester**:ab,ti OR **lancashire**:ab,ti OR **merseyside**:ab,ti OR (**blackburn**:ab,ti AND **darwen**:ab,ti) OR **blackpool**:ab,ti OR **chester**:ab,ti OR **liverpool**:ab,ti OR **sefton**:ab,ti OR **warrington**:ab,ti OR **wirral**:ab,ti OR **berkshire**:ab,ti OR **buckinghamshire**:ab,ti OR **oxfordshire**:ab,ti OR **hampshire**:ab,ti OR **'isle of wight'**:ab,ti OR **kent**:ab,ti OR **surrey**:ab,ti OR **sussex**:ab,ti OR (**brighton**:ab,ti AND **hove**:ab,ti) OR **'milton keynes'**:ab,ti OR **portsmouth**:ab,ti OR **southampton**:ab,ti OR **devon**:ab,ti OR **dorset**:ab,ti OR **somerset**:ab,ti OR **gloucestershire**:ab,ti OR **wiltshire**:ab,ti OR **bath**:ab,ti OR **bournemouth**:ab,ti OR **poole**:ab,ti OR **bristol**:ab,ti OR **plymouth**:ab,ti OR **swindon**:ab,ti OR **torbay**:ab,ti OR **herefordshire**:ab,ti OR **staffordshire**:ab,ti OR **birmingham**:ab,ti OR **coventry**:ab,ti OR **dudley**:ab,ti OR **sandwell**:ab,ti OR **shropshire**:ab,ti OR **solihull**:ab,ti OR **'stoke on trent'**:ab,ti OR **telford**:ab,ti OR **wrekin**:ab,ti OR **walsall**:ab,ti OR **warwickshire**:ab,ti OR **wolverhampton**:ab,ti OR **worcestershire**:ab,ti OR **barnsley**:ab,ti OR **doncaster**:ab,ti OR **rotherham**:ab,ti OR **bradford**:ab,ti OR **calderdale**:ab,ti OR **kirklees**:ab,ti OR **kingston**:ab,ti OR **leeds**:ab,ti OR **sheffield**:ab,ti OR **wakefield**:ab,ti OR (**york**:ab,ti NOT **'new york'**:ab,ti) OR **antrim**:ab,ti OR **ards**:ab,ti OR **armagh**:ab,ti OR **ballymena**:ab,ti OR **ballymoney**:ab,ti OR **banbridge**:ab,ti OR **carrickfergus**:ab,ti OR **castlereagh**:ab,ti OR **coleraine**:ab,ti OR **cookstown**:ab,ti OR **craigavon**:ab,ti OR (**down**:ab,ti AND (**district**:ab,ti OR **council**:ab,ti)) OR **dungannon**:ab,ti OR **fermanagh**:ab,ti OR **larne**:ab,ti OR **limavady**:ab,ti OR **lisburn**:ab,ti OR **magherafelt**:ab,ti OR **moyle**:ab,ti OR (**newry**:ab,ti AND **mourne**:ab,ti) OR **newtownabbey**:ab,ti OR **omagh**:ab,ti OR **strabane**:ab,ti OR **londonderry**:ab,ti OR **tyrone**:ab,ti OR **belfast**:ab,ti OR **aberdeen**:ab,ti OR **aberdeenshire**:ab,ti OR **angus**:ab,ti OR **dundee**:ab,ti OR (**argyll**:ab,ti AND **bute**:ab,ti) OR **clackmannanshire**:ab,ti OR **fife**:ab,ti OR **ayrshire**:ab,ti OR **dunbartonshire**:ab,ti OR **lothian**:ab,ti OR **renfrewshire**:ab,ti OR **edinburgh**:ab,ti OR **falkirk**:ab,ti OR **glasgow**:ab,ti OR **highland***:ab,ti OR **inverclyde**:ab,ti OR **midlothian**:ab,ti OR **moray**:ab,ti OR **lanarkshire**:ab,ti OR (**perth**:ab,ti AND **kinross**:ab,ti) OR **stirling**:ab,ti OR **'orkney islands'**:ab,ti OR **'eileanan siar'**:ab,ti OR **'shetland islands'**:ab,ti OR **bridgend**:ab,ti OR **'neath port talbot'**:ab,ti OR **cardiff**:ab,ti OR (**vale**:ab,ti AND **glamorgan**:ab,ti) OR **'central valleys'**:ab,ti OR **conwy**:ab,ti OR **denbighshire**:ab,ti OR **flintshire**:ab,ti OR **wrexham**:ab,ti OR **'gwent valleys'**:ab,ti OR **gwynedd**:ab,ti OR (**isle**:ab,ti AND **anglesey**:ab,ti) OR **'monmouthshire'**:ab,ti OR **'newport'**:ab,ti OR **powys**:ab,ti OR **swansea**:ab,ti OR **ceredigion**:ab,ti OR **carmarthenshire**:ab,ti OR **pembrokeshire**:ab,ti OR **'merthyr tydfil'**:ab,ti OR **'rhondda cynon taff'**:ab,ti OR **'blaenau gwent'**:ab,ti OR **caerphilly**:ab,ti OR **torfaen**:ab,ti OR **caithness**:ab,ti OR **'sutherland and ross'**:ab,ti OR **cromarty**:ab,ti OR **teeside**:ab,ti OR **tyneside**:ab,ti OR **wearside**:ab,ti OR **'west mercia'**:ab,ti OR **avon**:ab,ti OR **ulster**:ab,ti OR **derry**:ab,ti OR **medway**:ab,ti OR **'east riding'**:ab,ti OR **'west riding'**:ab,ti OR **'lake district'**:ab,ti OR **'peak district'**:ab,ti OR **cumberland**:ab,ti OR **dartmoor**:ab,ti OR **exmoor**:ab,ti OR **sweden**:ad OR **sverige**:ad OR **swedish**:ad OR **svenska**:ad OR **stockholm***:ad OR **norrland**:ad OR **svealand**:ad OR **mellansverige**:ad OR **smaland**:ad OR **sydsverige**:ad OR **vastsverige**:ad OR **orebro**:ad OR **ostergotland***:ad OR **vastergotland***:ad OR **skara***:ad OR **bohus***:ad OR **dalsland**:ad OR **narke**:ad OR **sodermanland**:ad OR **uppsala**:ad OR **uppland**:ad OR **vastmanland***:ad OR **jamtland***:ad OR **harjedalen**:ad OR **vasternorrland***:ad OR **dalarna**:ad OR **kopparberg**:ad OR **gavleborg***:ad OR **gastrikland**:ad OR **halsingland**:ad OR **varmland***:ad OR **gotland***:ad OR **oland**:ad OR **jonkoping***:ad OR **kalmar***:ad OR **kronoberg***:ad OR **blekinge**:ad OR **skane***:ad OR **norrbotten***:ad OR **vasterbotten***:ad OR **lappland**:ad OR **angermanland**:ad OR **medelpad**:ad OR **halland***:ad OR **gotaland***:ad OR **gothenburg**:ad OR **goteborg***:ad OR **malmo***:ad OR **vasteras**:ad OR **linkoping**:ad OR **helsingborg**:ad OR **halsingborg**:ad OR **norrkoping**:ad OR **gavle**:ad OR **umea**:ad OR **lulea**:ad OR **karlstad**:ad OR **kalmar**:ad OR **huddinge**:ad OR **solna**:ad OR **ostersjo***:ad OR **malaren***:ad OR **malardalen**:ad OR **sweden**:ab,ti OR **sverige**:ab,ti OR **swedish**:ab,ti OR **svenska**:ab,ti OR **stockholm***:ab,ti OR **norrland**:ab,ti OR **svealand**:ab,ti OR **mellansverige**:ab,ti OR **smaland**:ab,ti OR **sydsverige**:ab,ti OR **vastsverige**:ab,ti OR **orebro**:ab,ti OR **ostergotland***:ab,ti OR **vastergotland***:ab,ti OR **skara***:ab,ti OR **bohus***:ab,ti OR **dalsland**:ab,ti OR **narke**:ab,ti OR **sodermanland**:ab,ti OR **uppsala**:ab,ti OR **uppland**:ab,ti OR **vastmanland***:ab,ti OR **jamtland***:ab,ti OR **harjedalen**:ab,ti OR **vasternorrland***:ab,ti OR **dalarna**:ab,ti OR **kopparberg**:ab,ti OR **gavleborg***:ab,ti OR **gastrikland**:ab,ti OR **halsingland**:ab,ti OR **varmland***:ab,ti OR **gotland***:ab,ti OR **oland**:ab,ti OR **jonkoping***:ab,ti OR **kalmar***:ab,ti OR **kronoberg***:ab,ti OR **blekinge**:ab,ti OR **skane***:ab,ti OR **norrbotten***:ab,ti OR **vasterbotten***:ab,ti OR **lappland**:ab,ti OR **angermanland**:ab,ti OR **medelpad**:ab,ti OR **halland***:ab,ti OR **gotaland***:ab,ti OR **gothenburg**:ab,ti OR **goteborg***:ab,ti OR **malmo***:ab,ti OR **vasteras**:ab,ti OR **linkoping**:ab,ti OR **helsingborg**:ab,ti OR **halsingborg**:ab,ti OR **norrkoping**:ab,ti OR **gavle**:ab,ti OR **umea**:ab,ti OR **lulea**:ab,ti OR **karlstad**:ab,ti OR **kalmar**:ab,ti OR **huddinge**:ab,ti OR **solna**:ab,ti OR **ostersjo***:ab,ti OR **malaren***:ab,ti OR **malardalen**:ab,ti OR **spain**:ad OR **espana**:ad OR **spanish**:ad OR **espanol***:ad OR **spaniard***:ad OR **madrid**:ad OR **andalucia**:ad OR **andalusia**:ad OR **aragon**:ad OR **cantabria**:ad OR **canarias**:ad OR **'canary islands'**:ad OR **'castile and leon'**:ad OR **'castilla y leon'**:ad OR **'castile la mancha'**:ad OR **'castilla la mancha'**:ad OR **cataluna**:ad OR **catalonia**:ad OR **ceuta**:ad OR **melilla**:ad OR **navarra**:ad OR **navarre**:ad OR **valencian**:ad OR **extremadura**:ad OR **galicia**:ad OR **balears**:ad OR **'balearic islands'**:ad OR **baleares**:ad OR **'la rioja'**:ad OR **'pais vasco'**:ad OR **'basque country'**:ad OR **coruna**:ad OR **alava**:ad OR **araba**:ad OR **albacete**:ad OR **alicante**:ad OR **alacant**:ad OR **almeria**:ad OR **asturias**:ad OR **avila**:ad OR **badajoz**:ad OR **badajos**:ad OR **barcelona**:ad OR **burgos**:ad OR **caceres**:ad OR **cadiz**:ad OR **castellon**:ad OR **castello**:ad OR **'ciudad real'**:ad OR **cordoba**:ad OR **cuenca**:ad OR **eivissa**:ad OR **ibiza**:ad OR **formentera**:ad OR **'el hierro'**:ad OR **fuerteventura**:ad OR **girona**:ad OR **gerona**:ad OR **'gran canaria'**:ad OR **granada**:ad OR **guadalajara**:ad OR **guipuzcoa**:ad OR **gipuzkoa**:ad OR **huelva**:ad OR **huesca**:ad OR **jaen**:ad OR **'la gomera'**:ad OR **'la palma'**:ad OR **lanzarote**:ad OR **leon**:ad OR **lleida**:ad OR **lerida**:ad OR **lugo**:ad OR **malaga**:ad OR **mallorca**:ad OR **majorca**:ad OR **menorca**:ad OR **minorca**:ad OR **murcia**:ad OR **ourense**:ad OR **orense**:ad OR **palencia**:ad OR **pontevedra**:ad OR **salamanca**:ad OR **segovia**:ad OR **sevilla**:ad OR **seville**:ad OR **soria**:ad OR **tarragona**:ad OR **tenerife**:ad OR **teruel**:ad OR **toledo**:ad OR **valencia**:ad OR **valladolid**:ad OR **vizcaya**:ad OR **biscay**:ad OR **zamora**:ad OR **zaragoza**:ad OR **saragossa**:ad OR **bilbao**:ad OR **bilbo**:ad OR **compostela**:ad OR **'san sebastian'**:ad OR **donostia**:ad OR **vitoria**:ad OR **oviedo**:ad OR **pamplona**:ad OR **logrono**:ad OR **gasteiz**:ad OR **spain**:ab,ti OR **espana**:ab,ti OR **spanish**:ab,ti OR **espanol***:ab,ti OR **spaniard***:ab,ti OR **madrid**:ab,ti OR **andalucia**:ab,ti OR **andalusia**:ab,ti OR **aragon**:ab,ti OR **cantabria**:ab,ti OR **canarias**:ab,ti OR **'canary islands'**:ab,ti OR **'castile and leon'**:ab,ti OR **'castilla y leon'**:ab,ti OR **'castile la mancha'**:ab,ti OR **'castilla la mancha'**:ab,ti OR **cataluna**:ab,ti OR **catalonia**:ab,ti OR **ceuta**:ab,ti OR **melilla**:ab,ti OR **navarra**:ab,ti OR **navarre**:ab,ti OR **valencian**:ab,ti OR **extremadura**:ab,ti OR **galicia**:ab,ti OR **balears**:ab,ti OR **'balearic islands'**:ab,ti OR **baleares**:ab,ti OR **'la rioja'**:ab,ti OR **'pais vasco'**:ab,ti OR **'basque country'**:ab,ti OR **coruna**:ab,ti OR **alava**:ab,ti OR **araba**:ab,ti OR **albacete**:ab,ti OR **alicante**:ab,ti OR **alacant**:ab,ti OR **almeria**:ab,ti OR **asturias**:ab,ti OR **avila**:ab,ti OR **badajoz**:ab,ti OR **badajos**:ab,ti OR **barcelona**:ab,ti OR **burgos**:ab,ti OR **caceres**:ab,ti OR **cadiz**:ab,ti OR **castellon**:ab,ti OR **castello**:ab,ti OR **'ciudad real'**:ab,ti OR (**cordoba**:ab,ti NOT **argent***:ab,ti) OR **cuenca**:ab,ti OR **eivissa**:ab,ti OR **ibiza**:ab,ti OR **formentera**:ab,ti OR **'el hierro'**:ab,ti OR **fuerteventura**:ab,ti OR **girona**:ab,ti OR **gerona**:ab,ti OR **'gran canaria'**:ab,ti OR **granada**:ab,ti OR (**guadalajara**:ab,ti NOT **mexic***:ab,ti) OR **guipuzcoa**:ab,ti OR **gipuzkoa**:ab,ti OR **huelva**:ab,ti OR **huesca**:ab,ti OR **jaen**:ab,ti OR **'la gomera'**:ab,ti OR **'la palma'**:ab,ti OR **lanzarote**:ab,ti OR **leon**:ab,ti OR **lleida**:ab,ti OR **lerida**:ab,ti OR **lugo**:ab,ti OR **malaga**:ab,ti OR **mallorca**:ab,ti OR **majorca**:ab,ti OR **menorca**:ab,ti OR **minorca**:ab,ti OR **murcia**:ab,ti OR **ourense**:ab,ti OR **orense**:ab,ti OR **palencia**:ab,ti OR **pontevedra**:ab,ti OR **salamanca**:ab,ti OR **segovia**:ab,ti OR **sevilla**:ab,ti OR **seville**:ab,ti OR **soria**:ab,ti OR **tarragona**:ab,ti OR **tenerife**:ab,ti OR **teruel**:ab,ti OR **toledo**:ab,ti OR **valencia**:ab,ti OR **valladolid**:ab,ti OR **vizcaya**:ab,ti OR **biscay**:ab,ti OR **zamora**:ab,ti OR **zaragoza**:ab,ti OR **saragossa**:ab,ti OR **bilbao**:ab,ti OR **bilbo**:ab,ti OR **compostela**:ab,ti OR **'san sebastian'**:ab,ti OR **donostia**:ab,ti OR **vitoria**:ab,ti OR **oviedo**:ab,ti OR **pamplona**:ab,ti OR **logrono**:ab,ti OR **gasteiz**:ab,ti OR **slovenia***:ad OR **slovenija**:ad OR **ljubljana**:ad OR **gorenjska**:ad OR **carniola**:ad OR **goriska**:ad OR **gorizia**:ad OR **koroska**:ad OR **carinthia**:ad OR **'notranjsko kraska'**:ad OR **'obalno kraska'**:ad OR **'coastal krast'**:ad OR **podravska**:ad OR **pomurska**:ad OR **savinjska**:ad OR **spodnjeposavska**:ad OR **zasavska**:ad OR **osrednjeslovenska**:ad OR **maribor**:ad OR **celje**:ad OR **kranj**:ad OR **velenje**:ad OR **koper**:ad OR **capodistria**:ad OR **'novo mesto'**:ad OR **ptuj**:ad OR **trbovlje**:ad OR **kamnik**:ad OR **murska**:ad OR **sobota**:ad OR **'nova gorica'**:ad OR **slovenia***:ab,ti OR **slovenija**:ab,ti OR **ljubljana**:ab,ti OR **gorenjska**:ab,ti OR **carniola**:ab,ti OR **goriska**:ab,ti OR **gorizia**:ab,ti OR **koroska**:ab,ti OR **carinthia**:ab,ti OR **'notranjsko kraska'**:ab,ti OR **'obalno kraska'**:ab,ti OR **'coastal krast'**:ab,ti OR **podravska**:ab,ti OR **pomurska**:ab,ti OR **savinjska**:ab,ti OR **spodnjeposavska**:ab,ti OR **zasavska**:ab,ti OR **osrednjeslovenska**:ab,ti OR **maribor**:ab,ti OR **celje**:ab,ti OR **kranj**:ab,ti OR **velenje**:ab,ti OR **koper**:ab,ti OR **capodistria**:ab,ti OR **'novo mesto'**:ab,ti OR **ptuj**:ab,ti OR **trbovlje**:ab,ti OR **kamnik**:ab,ti OR **murska**:ab,ti OR **sobota**:ab,ti OR **'nova gorica'**:ab,ti OR **slovakia**:ab,ti OR **slovensk***:ab,ti OR **slovak***:ab,ti OR **bratislav***:ab,ti OR **nitrian***:ab,ti OR **nitra**:ab,ti OR **trencian***:ab,ti OR **trencin**:ab,ti OR **banskobystri***:ab,ti OR **'banska bystrica'**:ab,ti OR **zilina**:ab,ti OR **zilin***:ab,ti OR **trnava**:ab,ti OR **trnav***:ab,ti OR **presov**:ab,ti OR **presov***:ab,ti OR **kosic***:ab,ti OR (**martin**:ab,ti AND (**city**:ab,ti OR **svaty**:ab,ti)) OR **poprad**:ab,ti OR **slovakia**:ad OR **slovensk***:ad OR **slovak***:ad OR **bratislav***:ad OR **nitrian***:ad OR **nitra**:ad OR **trencian***:ad OR **trencin**:ad OR **banskobystri***:ad OR **'banska bystrica'**:ad OR **zilina**:ad OR **zilin***:ad OR **trnava**:ad OR **trnav***:ad OR **presov***:ad OR **kosic***:ad OR (**martin**:ad AND (**city**:ad OR **svaty**:ad)) OR **poprad**:ad \|  \| \| #1.6 \| **italy**:ab,ti OR **italia***:ab,ti OR **rome**:ab,ti OR **roma**:ab,ti OR **abruzzo**:ab,ti OR **abruzzi**:ab,ti OR **basilicata**:ab,ti OR **lucania**:ab,ti OR **calabria**:ab,ti OR **campania**:ab,ti OR **'emilia romagna'**:ab,ti OR **'friuli venezia giulia'**:ab,ti OR **lazio**:ab,ti OR **latium**:ab,ti OR **liguria***:ab,ti OR **lombardy**:ab,ti OR **lombardia**:ab,ti OR **marche**:ab,ti OR **marches**:ab,ti OR **molisano**:ab,ti OR **molise**:ab,ti OR **piedmont***:ab,ti OR **piemonte**:ab,ti OR **sardinia**:ab,ti OR **sardegna**:ab,ti OR **sicily**:ab,ti OR **sicilia**:ab,ti OR **toscana**:ab,ti OR **tuscany**:ab,ti OR **trentino**:ab,ti OR **trento**:ab,ti OR **umbria**:ab,ti OR **veneto**:ab,ti OR **triveneto**:ab,ti OR **puglia**:ab,ti OR **apulia**:ab,ti OR **bolzano**:ab,ti OR **bozen**:ab,ti OR **milan**:ab,ti OR **milano**:ab,ti OR **naples**:ab,ti OR **napoli**:ab,ti OR **turin**:ab,ti OR **torino**:ab,ti OR **palermo**:ab,ti OR **genoa**:ab,ti OR **genova**:ab,ti OR **florence**:ab,ti OR **firenze**:ab,ti OR **bari**:ab,ti OR **catania**:ab,ti OR **venezia**:ab,ti OR **venice**:ab,ti OR **padova**:ab,ti OR **padua**:ab,ti OR **siena**:ab,ti OR **bologna**:ab,ti OR **trieste**:ab,ti OR **urbino**:ab,ti OR **aosta**:ab,ti OR **aoste**:ab,ti OR **perugia**:ab,ti OR **brescia**:ab,ti OR **cagliari**:ab,ti OR **catanzaro**:ab,ti OR **'l aquila'**:ab,ti OR **ancona**:ab,ti OR **italy**:ad OR **italia***:ad OR **rome**:ad OR **roma**:ad OR **abruzzo**:ad OR **abruzzi**:ad OR **basilicata**:ad OR **lucania**:ad OR **calabria**:ad OR **campania**:ad OR **'emilia romagna'**:ad OR **'friuli venezia giulia'**:ad OR **lazio**:ad OR **latium**:ad OR **liguria***:ad OR **lombardy**:ad OR **lombardia**:ad OR **marche**:ad OR **marches**:ad OR **molisano**:ad OR **molise**:ad OR **piedmont***:ad OR **piemonte**:ad OR **sardinia**:ad OR **sardegna**:ad OR **sicily**:ad OR **sicilia**:ad OR **toscana**:ad OR **tuscany**:ad OR **trentino**:ad OR **trento**:ad OR **umbria**:ad OR **veneto**:ad OR **triveneto**:ad OR **puglia**:ad OR **apulia**:ad OR **bolzano**:ad OR **bozen**:ad OR **milan**:ad OR **milano**:ad OR **naples**:ad OR **napoli**:ad OR **turin**:ad OR **torino**:ad OR **palermo**:ad OR **genoa**:ad OR **genova**:ad OR **florence**:ad OR **firenze**:ad OR **bari**:ad OR **catania**:ad OR **venezia**:ad OR **venice**:ad OR **padova**:ad OR **padua**:ad OR **siena**:ad OR **bologna**:ad OR **trieste**:ad OR **urbino**:ad OR **aosta**:ad OR **aoste**:ad OR **perugia**:ad OR **brescia**:ad OR **cagliari**:ad OR **catanzaro**:ad OR **'l aquila'**:ad OR **ancona**:ad OR **ireland**:ab,ti OR **eire**:ab,ti OR **irish***:ab,ti OR **dublin**:ab,ti OR **fingal**:ab,ti OR **'dun laoghaire'**:ab,ti OR **wicklow**:ab,ti OR **wexford**:ab,ti OR **carlow**:ab,ti OR **kildare**:ab,ti OR **meath**:ab,ti OR **louth**:ab,ti OR **monaghan**:ab,ti OR **cavan**:ab,ti OR **longford**:ab,ti OR **westmeath**:ab,ti OR **offaly**:ab,ti OR **laois**:ab,ti OR **kilkenny**:ab,ti OR **waterford**:ab,ti OR **cork**:ab,ti OR **kerry**:ab,ti OR **limerick**:ab,ti OR **tipperary**:ab,ti OR **clare**:ab,ti OR **galway**:ab,ti OR **mayo**:ab,ti OR **roscommon**:ab,ti OR **sligo**:ab,ti OR **leitrim**:ab,ti OR **donegal**:ab,ti OR **drogheda**:ab,ti OR **dundalk**:ab,ti OR **swords**:ab,ti OR **bray**:ab,ti OR **navan**:ab,ti OR **leinster**:ab,ti OR **connacht**:ab,ti OR **ireland**:ad OR **eire**:ad OR **irish***:ad OR **dublin**:ad OR **fingal**:ad OR **'dun laoghaire'**:ad OR **wicklow**:ad OR **wexford**:ad OR **carlow**:ad OR **kildare**:ad OR **meath**:ad OR **louth**:ad OR **monaghan**:ad OR **cavan**:ad OR **longford**:ad OR **westmeath**:ad OR **offaly**:ad OR **laois**:ad OR **kilkenny**:ad OR **waterford**:ad OR **cork**:ad OR **kerry**:ad OR **limerick**:ad OR **tipperary**:ad OR **clare**:ad OR **galway**:ad OR **mayo**:ad OR **roscommon**:ad OR **sligo**:ad OR **leitrim**:ad OR **donegal**:ad OR **drogheda**:ad OR **dundalk**:ad OR **swords**:ad OR **bray**:ad OR **navan**:ad OR **leinster**:ad OR **connacht**:ad OR **hungar***:ab,ti OR **budapest**:ab,ti OR **transdanubia**:ab,ti OR **magyarorszag**:ab,ti OR **magyar**:ab,ti OR **dunantuli**:ab,ti OR **dunantul**:ab,ti OR **'great plain'**:ab,ti OR **'alfold es eszak'**:ab,ti OR **'eszak alfold'**:ab,ti OR **'del alfold'**:ab,ti OR **bacs**:ab,ti OR **kiskun**:ab,ti OR **'northen alfold'**:ab,ti OR **'sourthen alfold'**:ab,ti OR **baranya**:ab,ti OR **bekes**:ab,ti OR **borsod**:ab,ti OR **abauj**:ab,ti OR **zemplen**:ab,ti OR **fovaros**:ab,ti OR **csongrad**:ab,ti OR **fejer**:ab,ti OR **moson**:ab,ti OR **sopron**:ab,ti OR **hajdu**:ab,ti OR **bihar**:ab,ti OR **heves**:ab,ti OR **'jasz nagykun szolnok'**:ab,ti OR **komarom**:ab,ti OR **esztergom**:ab,ti OR **nograd**:ab,ti OR **pest**:ab,ti OR **somogy**:ab,ti OR **szabolcs**:ab,ti OR **szatmar**:ab,ti OR **bereg**:ab,ti OR **tolna**:ab,ti OR **vas**:ab,ti OR **veszprem**:ab,ti OR **zala**:ab,ti OR **zalaegerszeg**:ab,ti OR **debrecen**:ab,ti OR **miskolc**:ab,ti OR **szeged**:ab,ti OR **pecs**:ab,ti OR **gyor**:ab,ti OR **nyiregyhaza**:ab,ti OR **kecskemet**:ab,ti OR **szekesfehervar**:ab,ti OR **szombathely**:ab,ti OR **bekescsaba**:ab,ti OR **eger**:ab,ti OR **tatabanya**:ab,ti OR **salgotarjan**:ab,ti OR **kaposvar**:ab,ti OR **szekszard**:ab,ti OR **hungar***:ad OR **budapest**:ad OR **transdanubia**:ad OR **magyarorszag**:ad OR **magyar**:ad OR **dunantuli**:ad OR **dunantul**:ad OR **'great plain'**:ad OR **'alfold es eszak'**:ad OR **'eszak alfold'**:ad OR **'del alfold'**:ad OR **bacs**:ad OR **kiskun**:ad OR **'northen alfold'**:ad OR **'sourthen alfold'**:ad OR **baranya**:ad OR **bekes**:ad OR **borsod**:ad OR **abauj**:ad OR **zemplen**:ad OR **fovaros**:ad OR **csongrad**:ad OR **fejer**:ad OR **moson**:ad OR **sopron**:ad OR **hajdu**:ad OR **bihar**:ad OR **heves**:ad OR **'jasz nagykun szolnok'**:ad OR **komarom**:ad OR **esztergom**:ad OR **nograd**:ad OR **pest**:ad OR **somogy**:ad OR **szabolcs**:ad OR **szatmar**:ad OR **bereg**:ad OR **tolna**:ad OR **vas**:ad OR **veszprem**:ad OR **zala**:ad OR **zalaegerszeg**:ad OR **debrecen**:ad OR **miskolc**:ad OR **szeged**:ad OR **pecs**:ad OR **gyor**:ad OR **nyiregyhaza**:ad OR **kecskemet**:ad OR **szekesfehervar**:ad OR **szombathely**:ad OR **bekescsaba**:ad OR **eger**:ad OR **tatabanya**:ad OR **salgotarjan**:ad OR **kaposvar**:ad OR **szekszard**:ad OR **greece**:ad OR **'hellenic republic'**:ad OR **greek***:ad OR **ellada**:ad OR **'elliniki dimokratia'**:ad OR **hellas**:ad OR **hellenes**:ad OR **attica**:ad OR **attiki**:ad OR **makedonia**:ad OR **macedonia**:ad OR **thraki**:ad OR **thrace**:ad OR **crete**:ad OR **kriti**:ad OR **epirus**:ad OR **ipeiros**:ad OR **'ionia nisia'**:ad OR **'ionion neson'**:ad OR **'ionian islands'**:ad OR **'north aegean'**:ad OR **'aegean islands'**:ad OR **'nisoi agaiou'**:ad OR **'notio aigaio'**:ad OR **peloponnese**:ad OR **peloponnisos**:ad OR **'voreio aigaio'**:ad OR **'south aegean'**:ad OR **thessaly**:ad OR **thessalia**:ad OR **cycklades**:ad OR **kiklades**:ad OR **dodecanese**:ad OR **dodekanisa**:ad OR **'mount athos'**:ad OR **'omicronros alphathos'**:ad OR **athens**:ad OR **athina**:ad OR **thessaloniki**:ad OR **thessalonica**:ad OR **patras**:ad OR **patra**:ad OR **heraklion**:ad OR **heraclion**:ad OR **iraklion**:ad OR **irakleion**:ad OR **iraklio**:ad OR **larissa**:ad OR **larisa**:ad OR **volos**:ad OR **rhodes**:ad OR **rodos**:ad OR **ioannina**:ad OR **janina**:ad OR **yannena**:ad OR **chania**:ad OR **chalcis**:ad OR **chalkida**:ad OR **alexandroupoli**:ad OR **greece**:ab,ti OR **'hellenic republic'**:ab,ti OR **greek***:ab,ti OR **ellada**:ab,ti OR **'elliniki dimokratia'**:ab,ti OR **hellas**:ab,ti OR **hellenes**:ab,ti OR **attica**:ab,ti OR **attiki**:ab,ti OR **makedonia**:ab,ti OR **macedonia**:ab,ti OR **thraki**:ab,ti OR **thrace**:ab,ti OR **crete**:ab,ti OR **kriti**:ab,ti OR **epirus**:ab,ti OR **ipeiros**:ab,ti OR **'ionia nisia'**:ab,ti OR **'ionion neson'**:ab,ti OR **'ionian islands'**:ab,ti OR **'north aegean'**:ab,ti OR **'aegean islands'**:ab,ti OR **'nisoi agaiou'**:ab,ti OR **'notio aigaio'**:ab,ti OR **peloponnese**:ab,ti OR **peloponnisos**:ab,ti OR **'voreio aigaio'**:ab,ti OR **'south aegean'**:ab,ti OR **thessaly**:ab,ti OR **thessalia**:ab,ti OR **cycklades**:ab,ti OR **kiklades**:ab,ti OR **dodecanese**:ab,ti OR **dodekanisa**:ab,ti OR **'mount athos'**:ab,ti OR **'omicronros alphathos'**:ab,ti OR **athens**:ab,ti OR **athina**:ab,ti OR **thessaloniki**:ab,ti OR **thessalonica**:ab,ti OR **patras**:ab,ti OR **patra**:ab,ti OR **heraklion**:ab,ti OR **heraclion**:ab,ti OR **iraklion**:ab,ti OR **irakleion**:ab,ti OR **iraklio**:ab,ti OR **larissa**:ab,ti OR **larisa**:ab,ti OR **volos**:ab,ti OR **rhodes**:ab,ti OR **rodos**:ab,ti OR **ioannina**:ab,ti OR **janina**:ab,ti OR **yannena**:ab,ti OR **chania**:ab,ti OR **chalcis**:ab,ti OR **chalkida**:ab,ti OR **alexandroupoli**:ab,ti OR **german***:ad OR **deutschland**:ad OR **deutsch***:ad OR **bundesrepublik**:ad OR **westdeutschland**:ad OR **ostdeutschland**:ad OR **baden**:ad OR **wuerttemberg**:ad OR **wurttemberg**:ad OR **bayern**:ad OR **bavaria**:ad OR **berlin**:ad OR **brandenburg**:ad OR **bremen**:ad OR **hamburg**:ad OR **hessen**:ad OR **hesse**:ad OR **hessia**:ad OR **mecklenburg**:ad OR **vorpommern**:ad OR **pomerania**:ad OR **niedersachsen**:ad OR **neddersassen**:ad OR **saxony**:ad OR **niederbayern**:ad OR **'north rhine'**:ad OR **westphalia**:ad OR **westfalen**:ad OR **'rhineland palatinate'**:ad OR **'rheinland pfalz'**:ad OR **saarland**:ad OR **sachsen**:ad OR **'schleswig holstein'**:ad OR **thuringia**:ad OR **thuringen**:ad OR **thueringen**:ad OR **freiburg**:ad OR **karlsruhe**:ad OR **calsruhe**:ad OR **stuttgart**:ad OR **tubingen**:ad OR **oberbayern**:ad OR **'upper palatinate'**:ad OR **oberpfalz**:ad OR **franken**:ad OR **franconia**:ad OR **oberfranken**:ad OR **mittelfranken**:ad OR **schwaben**:ad OR **unterfranken**:ad OR **swabia**:ad OR **darmstadt**:ad OR **giessen**:ad OR **kassel**:ad OR **arnsberg**:ad OR **cologne**:ad OR **koln**:ad OR **koeln**:ad OR **detmold**:ad OR **dusseldorf**:ad OR **duesseldorf**:ad OR **munster**:ad OR **muenster**:ad OR **munich**:ad OR **munchen**:ad OR **muenchen**:ad OR **frankfurt**:ad OR **dortmund**:ad OR **essen**:ad OR **nurnberg**:ad OR **nuernberg**:ad OR **nuremberg**:ad OR **hanover**:ad OR **hannover**:ad OR **leipzig**:ad OR **dresden**:ad OR **ruhrgebiet**:ad OR **revier**:ad OR **ruhrpott**:ad OR **pott**:ad OR **ruhr**:ad OR **german***:ab,ti OR **deutschland**:ab,ti OR **deutsch***:ab,ti OR **bundesrepublik**:ab,ti OR **westdeutschland**:ab,ti OR **ostdeutschland**:ab,ti OR **baden**:ab,ti OR **wuerttemberg**:ab,ti OR **wurttemberg**:ab,ti OR **bayern**:ab,ti OR **bavaria**:ab,ti OR **berlin**:ab,ti OR **brandenburg**:ab,ti OR **bremen**:ab,ti OR **hamburg**:ab,ti OR **hessen**:ab,ti OR **hesse**:ab,ti OR **hessia**:ab,ti OR **mecklenburg**:ab,ti OR **vorpommern**:ab,ti OR **pomerania**:ab,ti OR **niedersachsen**:ab,ti OR **neddersassen**:ab,ti OR **saxony**:ab,ti OR **niederbayern**:ab,ti OR **'north rhine'**:ab,ti OR **westphalia**:ab,ti OR **westfalen**:ab,ti OR **'rhineland palatinate'**:ab,ti OR **'rheinland pfalz'**:ab,ti OR **saarland**:ab,ti OR **sachsen**:ab,ti OR **'schleswig holstein'**:ab,ti OR **thuringia**:ab,ti OR **thuringen**:ab,ti OR **thueringen**:ab,ti OR **freiburg**:ab,ti OR **karlsruhe**:ab,ti OR **calsruhe**:ab,ti OR **stuttgart**:ab,ti OR **tubingen**:ab,ti OR **oberbayern**:ab,ti OR **'upper palatinate'**:ab,ti OR **oberpfalz**:ab,ti OR **franken**:ab,ti OR **franconia**:ab,ti OR **oberfranken**:ab,ti OR **mittelfranken**:ab,ti OR **schwaben**:ab,ti OR **unterfranken**:ab,ti OR **swabia**:ab,ti OR **darmstadt**:ab,ti OR **giessen**:ab,ti OR **kassel**:ab,ti OR **arnsberg**:ab,ti OR **cologne**:ab,ti OR **koln**:ab,ti OR **koeln**:ab,ti OR **detmold**:ab,ti OR **dusseldorf**:ab,ti OR **duesseldorf**:ab,ti OR **munster**:ab,ti OR **muenster**:ab,ti OR **munich**:ab,ti OR **munchen**:ab,ti OR **muenchen**:ab,ti OR **frankfurt**:ab,ti OR **dortmund**:ab,ti OR **essen**:ab,ti OR **nurnberg**:ab,ti OR **nuernberg**:ab,ti OR **nuremberg**:ab,ti OR **hanover**:ab,ti OR **hannover**:ab,ti OR **leipzig**:ab,ti OR **dresden**:ab,ti OR **ruhrgebiet**:ab,ti OR **revier**:ab,ti OR **ruhrpott**:ab,ti OR **pott**:ab,ti OR **ruhr**:ab,ti OR **france**:ad OR **french***:ad OR **francais**:ad OR **alsace**:ad OR **aquitaine**:ad OR **auvergne**:ad OR **brittany**:ad OR **bretagne**:ad OR **bourgogne**:ad OR **burgundy**:ad OR **'champagne ardenne'**:ad OR **'franche comte'**:ad OR **'ile de france'**:ad OR **'languedoc roussillon'**:ad OR **limousin**:ad OR **lorraine**:ad OR **normandie**:ad OR **normandy**:ad OR **'midi pyrenees'**:ad OR **'nord pas de calais'**:ad OR **picardie**:ad OR **picardy**:ad OR **'poitou charentes'**:ad OR **provence**:ad OR **'rhone alpes'**:ad OR **corse**:ad OR **corsica**:ad OR **guiana**:ad OR **guyane**:ad OR **guadeloupe**:ad OR **martinique**:ad OR **reunion**:ad OR **mayotte**:ad OR **ain**:ad OR **aisne**:ad OR **allier**:ad OR **'alpes de haute provence'**:ad OR **'haute alpes'**:ad OR **'alpes maritimes'**:ad OR **ardeche**:ad OR **ardennes**:ad OR **ariege**:ad OR **aube**:ad OR **aude**:ad OR **aveyron**:ad OR **'bas rhin'**:ad OR **'bouches du rhone'**:ad OR **calvados**:ad OR **cantal**:ad OR **charente**:ad OR **cher**:ad OR **correze**:ad OR **'corse du sud'**:ad OR **'cote d or'**:ad OR **'cotes d armor'**:ad OR **'cote d azur'**:ad OR **creuse**:ad OR **'deux sevres'**:ad OR **dordogne**:ad OR **doubs**:ad OR **drome**:ad OR **essonne**:ad OR **eure**:ad OR **finistere**:ad OR **gard**:ad OR **gers**:ad OR **gironde**:ad OR **'haute corse'**:ad OR **'haute garonne'**:ad OR **'haute marne'**:ad OR **'hautes alpes'**:ad OR **'haute saone'**:ad OR **'haute savoie'**:ad OR **'hautes pyrenees'**:ad OR **'haute vienne'**:ad OR **'haut rhin'**:ad OR **'hauts de seine'**:ad OR **herault**:ad OR **'ille et vilaine'**:ad OR **indre**:ad OR **isere**:ad OR **jura**:ad OR **landes**:ad OR **loire**:ad OR **loiret**:ad OR (**lot**:ad AND (**departement**:ad OR **department**:ad)) OR **'lot et garonne'**:ad OR **'loir et cher'**:ad OR **lozere**:ad OR **manche**:ad OR **marne**:ad OR **mayenne**:ad OR **'meurthe et moselle'**:ad OR **meuse**:ad OR **morbihan**:ad OR **moselle**:ad OR (**nord**:ad AND (**department**:ad OR **departement**:ad)) OR **nievre**:ad OR **oise**:ad OR **orne**:ad OR **'pas de calais'**:ad OR **paris**:ad OR **'puy de dome'**:ad OR **'pyrenees atlantiques'**:ad OR **'pyrenees orientales'**:ad OR **rhone**:ad OR **sarthe**:ad OR **savoie**:ad OR **'seine et marne'**:ad OR **'seine maritime'**:ad OR **somme**:ad OR **tarn**:ad OR **'territoire de belfort'**:ad OR **'val de marne'**:ad OR **'val d oise'**:ad OR **var**:ad OR **vaucluse**:ad OR **vendee**:ad OR **vienne**:ad OR **vosges**:ad OR **yonne**:ad OR **yvelines**:ad OR **marseille**:ad OR **lyon**:ad OR **nice**:ad OR **nantes**:ad OR **strasbourg**:ad OR **montpellier**:ad OR **bordeaux**:ad OR **lille**:ad OR **toulouse**:ad OR **france**:ab,ti OR **french***:ab,ti OR **francais**:ab,ti OR **alsace**:ab,ti OR **aquitaine**:ab,ti OR **auvergne**:ab,ti OR **brittany**:ab,ti OR **bretagne**:ab,ti OR **bourgogne**:ab,ti OR **burgundy**:ab,ti OR **'champagne ardenne'**:ab,ti OR **'franche comte'**:ab,ti OR **'ile de france'**:ab,ti OR **'languedoc roussillon'**:ab,ti OR **limousin**:ab,ti OR **lorraine**:ab,ti OR **normandie**:ab,ti OR **normandy**:ab,ti OR **'midi pyrenees'**:ab,ti OR **'nord pas de calais'**:ab,ti OR **picardie**:ab,ti OR **picardy**:ab,ti OR **'poitou charentes'**:ab,ti OR **provence**:ab,ti OR **'rhone alpes'**:ab,ti OR **corse**:ab,ti OR **corsica**:ab,ti OR **guiana**:ab,ti OR **guyane**:ab,ti OR **guadeloupe**:ab,ti OR **martinique**:ab,ti OR **reunion**:ab,ti OR **mayotte**:ab,ti OR **ain**:ab,ti OR **aisne**:ab,ti OR **allier**:ab,ti OR **'alpes de haute provence'**:ab,ti OR **'haute alpes'**:ab,ti OR **'alpes maritimes'**:ab,ti OR **ardeche**:ab,ti OR **ardennes**:ab,ti OR **ariege**:ab,ti OR **aube**:ab,ti OR **aude**:ab,ti OR **aveyron**:ab,ti OR **'bas rhin'**:ab,ti OR **'bouches du rhone'**:ab,ti OR **calvados**:ab,ti OR **cantal**:ab,ti OR **charente**:ab,ti OR **cher**:ab,ti OR **correze**:ab,ti OR **'corse du sud'**:ab,ti OR **'cote d or'**:ab,ti OR **'cotes d armor'**:ab,ti OR **'cote d azur'**:ab,ti OR **creuse**:ab,ti OR **'deux sevres'**:ab,ti OR **dordogne**:ab,ti OR **doubs**:ab,ti OR **drome**:ab,ti OR **essonne**:ab,ti OR **eure**:ab,ti OR **finistere**:ab,ti OR **gard**:ab,ti OR **gers**:ab,ti OR **gironde**:ab,ti OR **'haute corse'**:ab,ti OR **'haute garonne'**:ab,ti OR **'haute marne'**:ab,ti OR **'hautes alpes'**:ab,ti OR **'haute saone'**:ab,ti OR **'haute savoie'**:ab,ti OR **'hautes pyrenees'**:ab,ti OR **'haute vienne'**:ab,ti OR **'haut rhin'**:ab,ti OR **'hauts de seine'**:ab,ti OR **herault**:ab,ti OR **'ille et vilaine'**:ab,ti OR **indre**:ab,ti OR **isere**:ab,ti OR **jura**:ab,ti OR **landes**:ab,ti OR **loire**:ab,ti OR **loiret**:ab,ti OR (**lot**:ab,ti AND (**departement**:ab,ti OR **department**:ab,ti)) OR **'lot et garonne'**:ab,ti OR **'loir et cher'**:ab,ti OR **lozere**:ab,ti OR **manche**:ab,ti OR **marne**:ab,ti OR **mayenne**:ab,ti OR **'meurthe et moselle'**:ab,ti OR **meuse**:ab,ti OR **morbihan**:ab,ti OR **moselle**:ab,ti OR (**nord**:ab,ti AND (**department**:ab,ti OR **departement**:ab,ti)) OR **nievre**:ab,ti OR **oise**:ab,ti OR **orne**:ab,ti OR **'pas de calais'**:ab,ti OR **paris**:ab,ti OR **'puy de dome'**:ab,ti OR **'pyrenees atlantiques'**:ab,ti OR **'pyrenees orientales'**:ab,ti OR **rhone**:ab,ti OR **sarthe**:ab,ti OR **savoie**:ab,ti OR **'seine et marne'**:ab,ti OR **'seine maritime'**:ab,ti OR **somme**:ab,ti OR **tarn**:ab,ti OR **'territoire de belfort'**:ab,ti OR **'val de marne'**:ab,ti OR **'val d oise'**:ab,ti OR **var**:ab,ti OR **vaucluse**:ab,ti OR **vendee**:ab,ti OR **vienne**:ab,ti OR **vosges**:ab,ti OR **yonne**:ab,ti OR **yvelines**:ab,ti OR **marseille**:ab,ti OR **lyon**:ab,ti OR **nice**:ab,ti OR **nantes**:ab,ti OR **strasbourg**:ab,ti OR **montpellier**:ab,ti OR **bordeaux**:ab,ti OR **lille**:ab,ti OR **toulouse**:ab,ti OR **finland**:ab,ti OR **finnish***:ab,ti OR **suomi***:ab,ti OR **lapland**:ab,ti OR **lappi**:ab,ti OR **lappland**:ab,ti OR **ostrobothnia**:ab,ti OR **pohjanmaa**:ab,ti OR **osterbotten**:ab,ti OR **kainuu**:ab,ti OR **kajanaland***:ab,ti OR **karelia**:ab,ti OR **karjala**:ab,ti OR **karelen**:ab,ti OR **savonia**:ab,ti OR **savo**:ab,ti OR **savolax**:ab,ti OR **pirkanmaa**:ab,ti OR **birkaland**:ab,ti OR **satakunta**:ab,ti OR **satakunda**:ab,ti OR **tavastia**:ab,ti OR **tavastland**:ab,ti OR **'paijat hame'**:ab,ti OR **'kanta hame'**:ab,ti OR **uusimaa**:ab,ti OR **nyland**:ab,ti OR **kymenlaakso**:ab,ti OR **kymmenedalen**:ab,ti OR **aland**:ab,ti OR **ahvenanmaa**:ab,ti OR **helsinki**:ab,ti OR **helsingfors**:ab,ti OR **espoo**:ab,ti OR **esbo**:ab,ti OR **tampere**:ab,ti OR **tammerfors**:ab,ti OR **vantaa**:ab,ti OR **vanda**:ab,ti OR **oulu**:ab,ti OR **uleaborg**:ab,ti OR **turku**:ab,ti OR **abo**:ab,ti OR **jyvaskyla**:ab,ti OR **kuopio**:ab,ti OR **lathi**:ab,ti OR **lahtis**:ab,ti OR **kouvola**:ab,ti OR **finland**:ad OR **finnish***:ad OR **suomi***:ad OR **lapland**:ad OR **lappi**:ad OR **lappland**:ad OR **ostrobothnia**:ad OR **pohjanmaa**:ad OR **osterbotten**:ad OR **kainuu**:ad OR **kajanaland***:ad OR **karelia**:ad OR **karjala**:ad OR **karelen**:ad OR **savonia**:ad OR **savo**:ad OR **savolax**:ad OR **pirkanmaa**:ad OR **birkaland**:ad OR **satakunta**:ad OR **satakunda**:ad OR **tavastia**:ad OR **tavastland**:ad OR **'paijat hame'**:ad OR **'kanta hame'**:ad OR **uusimaa**:ad OR **nyland**:ad OR **kymenlaakso**:ad OR **kymmenedalen**:ad OR **aland**:ad OR **ahvenanmaa**:ad OR **helsinki**:ad OR **helsingfors**:ad OR **espoo**:ad OR **esbo**:ad OR **tampere**:ad OR **tammerfors**:ad OR **vantaa**:ad OR **vanda**:ad OR **oulu**:ad OR **uleaborg**:ad OR **turku**:ad OR **abo**:ad OR **jyvaskyla**:ad OR **kuopio**:ad OR **lathi**:ad OR **lahtis**:ad OR **kouvola**:ad OR **estonia***:ab,ti OR **eesti**:ab,ti OR **esti**:ab,ti OR **tallinn**:ab,ti OR **harju**:ab,ti OR **harjumaa**:ab,ti OR **hiiu**:ab,ti OR **hiiumaa**:ab,ti OR **'ida viru'**:ab,ti OR **'ida virumaa'**:ab,ti OR **jarvamaa**:ab,ti OR **jarva**:ab,ti OR **jogevamaa**:ab,ti OR **jogeva**:ab,ti OR **laanemma**:ab,ti OR **laane**:ab,ti OR **parnumaa**:ab,ti OR **polva**:ab,ti OR **polvamaa**:ab,ti OR **rapla**:ab,ti OR **raplamaa**:ab,ti OR **saare**:ab,ti OR **saaremaa**:ab,ti OR **tartu**:ab,ti OR **tartumaa**:ab,ti OR **valga**:ab,ti OR **valgamaa**:ab,ti OR **valgamaakond**:ab,ti OR **viljandimaa**:ab,ti OR **voru**:ab,ti OR **vorumaa**:ab,ti OR **narva**:ab,ti OR **parnu**:ab,ti OR **'kohtla jarve'**:ab,ti OR **viljandi**:ab,ti OR **rakvere**:ab,ti OR **maardu**:ab,ti OR **sillamae**:ab,ti OR **kuressaare**:ab,ti OR **estonia***:ad OR **eesti**:ad OR **esti**:ad OR **tallinn**:ad OR **harju**:ad OR **harjumaa**:ad OR **hiiu**:ad OR **hiiumaa**:ad OR **'ida viru'**:ad OR **'ida virumaa'**:ad OR **jarvamaa**:ad OR **jarva**:ad OR **jogevamaa**:ad OR **jogeva**:ad OR **laanemma**:ad OR **laane**:ad OR **parnumaa**:ad OR **polva**:ad OR **polvamaa**:ad OR **rapla**:ad OR **raplamaa**:ad OR **saare**:ad OR **saaremaa**:ad OR **tartu**:ad OR **tartumaa**:ad OR **valga**:ad OR **valgamaa**:ad OR **valgamaakond**:ad OR **viljandimaa**:ad OR **voru**:ad OR **vorumaa**:ad OR **narva**:ad OR **parnu**:ad OR **'kohtla jarve'**:ad OR **viljandi**:ad OR **rakvere**:ad OR **maardu**:ad OR **sillamae**:ad OR **kuressaare**:ad \|  \| \| #1.5 \| **romania**:ab,ti OR **rumania**:ab,ti OR **roumania**:ab,ti OR **romanian**:ab,ti OR **roman**:ab,ti OR **bucharest**:ab,ti OR **bucuresti**:ab,ti OR **alba**:ab,ti OR **brasov**:ab,ti OR **covasna**:ab,ti OR **harghita**:ab,ti OR **mures**:ab,ti OR **sibiu**:ab,ti OR **bacau**:ab,ti OR **botosani**:ab,ti OR **iasi**:ab,ti OR **neamt**:ab,ti OR **suceava**:ab,ti OR **vaslui**:ab,ti OR **bihor**:ab,ti OR **'bistrita nasaud'**:ab,ti OR **cluj**:ab,ti OR **maramures**:ab,ti OR **salaj**:ab,ti OR **'satu mare'**:ab,ti OR **arges**:ab,ti OR **calarasi**:ab,ti OR **dambovita**:ab,ti OR **giurgiu**:ab,ti OR **ialomita**:ab,ti OR **prahova**:ab,ti OR **teleorman**:ab,ti OR **braila**:ab,ti OR **buzau**:ab,ti OR **galati**:ab,ti OR **tulcea**:ab,ti OR **vrancea**:ab,ti OR **dolj**:ab,ti OR **gorj**:ab,ti OR **mehedinti**:ab,ti OR (**olt**:ab,ti AND (**river**:ab,ti OR **county**:ab,ti OR **region**:ab,ti OR **judetul**:ab,ti OR **raul**:ab,ti)) OR **valcea**:ab,ti OR **vilcea**:ab,ti OR **arad**:ab,ti OR **'caras-severin'**:ab,ti OR **hunedoara**:ab,ti OR **timis**:ab,ti OR **ilfov**:ab,ti OR **timisoara**:ab,ti OR **constanta**:ab,ti OR **craiova**:ab,ti OR **ploiesti**:ab,ti OR **oradea**:ab,ti OR **'cluj-napoca'**:ab,ti OR **deva**:ab,ti OR **romania**:ad OR **rumania**:ad OR **roumania**:ad OR **romanian**:ad OR **roman**:ad OR **bucharest**:ad OR **bucuresti**:ad OR **alba**:ad OR **brasov**:ad OR **covasna**:ad OR **harghita**:ad OR **mures**:ad OR **sibiu**:ad OR **bacau**:ad OR **botosani**:ad OR **iasi**:ad OR **neamt**:ad OR **suceava**:ad OR **vaslui**:ad OR **bihor**:ad OR **'bistrita nasaud'**:ad OR **cluj**:ad OR **maramures**:ad OR **salaj**:ad OR **'satu mare'**:ad OR **arges**:ad OR **calarasi**:ad OR **dambovita**:ad OR **giurgiu**:ad OR **ialomita**:ad OR **prahova**:ad OR **teleorman**:ad OR **braila**:ad OR **buzau**:ad OR **galati**:ad OR **tulcea**:ad OR **vrancea**:ad OR **dolj**:ad OR **gorj**:ad OR **mehedinti**:ad OR (**olt**:ad AND (**river**:ad OR **county**:ad OR **region**:ad OR **judetul**:ad OR **raul**:ad)) OR **valcea**:ad OR **vilcea**:ad OR **arad**:ad OR **'caras-severin'**:ad OR **hunedoara**:ad OR **timis**:ad OR **ilfov**:ad OR **timisoara**:ad OR **constanta**:ad OR **craiova**:ad OR **ploiesti**:ad OR **oradea**:ad OR **'cluj-napoca'**:ad OR **deva**:ad OR **portugal**:ab,ti OR **portugues***:ab,ti OR **lisboa**:ab,ti OR **lisbon**:ab,ti OR **leira**:ab,ti OR **santarem**:ab,ti OR **beja**:ab,ti OR **faro**:ab,ti OR **evora**:ab,ti OR **portalegre**:ab,ti OR **'castelo branco'**:ab,ti OR **guarda**:ab,ti OR **aveiro**:ab,ti OR **viseu**:ab,ti OR **braganca**:ab,ti OR **'vila real'**:ab,ti OR **'viana do castelo'**:ab,ti OR **alentejo**:ab,ti OR **azores**:ab,ti OR **acores**:ab,ti OR **madeira**:ab,ti OR **'os montes'**:ab,ti OR (**ave**:ab,ti AND (**community**:ab,ti OR **intermunicipal**:ab,ti OR **comunidade**:ab,ti)) OR **mondego**:ab,ti OR **vouga**:ab,ti OR **beira**:ab,ti OR **cavado**:ab,ti OR **lafoes**:ab,ti OR **douro**:ab,ti OR **porto**:ab,ti OR **tejo**:ab,ti OR **minho**:ab,ti OR **setubal**:ab,ti OR **pinhal**:ab,ti OR **'serra da estrela'**:ab,ti OR **tamega**:ab,ti OR **algarve**:ab,ti OR **gaia**:ab,ti OR **amadora**:ab,ti OR **braga**:ab,ti OR (**agualva**:ab,ti AND **cacem**:ab,ti) OR **funchal**:ab,ti OR **coimbra**:ab,ti OR **almada**:ab,ti OR **portugal**:ad OR **portugues***:ad OR **lisboa**:ad OR **lisbon**:ad OR **leira**:ad OR **santarem**:ad OR **beja**:ad OR **faro**:ad OR **evora**:ad OR **portalegre**:ad OR **'castelo branco'**:ad OR **guarda**:ad OR **aveiro**:ad OR **viseu**:ad OR **braganca**:ad OR **'vila real'**:ad OR **'viana do castelo'**:ad OR **alentejo**:ad OR **azores**:ad OR **acores**:ad OR **madeira**:ad OR **'os montes'**:ad OR (**ave**:ad AND (**community**:ad OR **intermunicipal**:ad OR **comunidade**:ad)) OR **mondego**:ad OR **vouga**:ad OR **beira**:ad OR **cavado**:ad OR **lafoes**:ad OR **douro**:ad OR **porto**:ad OR **tejo**:ad OR **minho**:ad OR **setubal**:ad OR **pinhal**:ad OR **'serra da estrela'**:ad OR **tamega**:ad OR **algarve**:ad OR **gaia**:ad OR **amadora**:ad OR **braga**:ad OR (**agualva**:ad AND **cacem**:ad) OR **funchal**:ad OR **coimbra**:ad OR **almada**:ad OR **poland**:ad OR **polska**:ad OR **polish**:ad OR **polski**:ad OR **pole**:ad OR **poles**:ad OR **polak**:ad OR **polka**:ad OR **polacy**:ad OR **warsaw**:ad OR **warszawa**:ad OR **wielkopolskie**:ad OR **pomerania***:ad OR **pomorskie**:ad OR **kuyavian**:ad OR **kujawsko**:ad OR **malopolskie**:ad OR **lodz**:ad OR **lodzkie**:ad OR **silesia***:ad OR **dolnoslaskie**:ad OR **lublin**:ad OR **lubelskie**:ad OR **lubus**:ad OR **lubusz**:ad OR **lubuskie**:ad OR **masovia**:ad OR **mazowske**:ad OR **masovian**:ad OR **mazowieckie**:ad OR **opole**:ad OR **opolskie**:ad OR **podlaskie**:ad OR **podlachia**:ad OR **podlasie**:ad OR **subcarpathian***:ad OR **carpathian***:ad OR **podkarpackie**:ad OR **swietokrzyskie**:ad OR **slaskie**:ad OR **slask**:ad OR **'varmia mazuria'**:ad OR **'varmian mazurian'**:ad OR **'varmia masuria'**:ad OR **'varmian masurian'**:ad OR **'warmia mazury'**:ad OR **'warminsko mazurskie'**:ad OR **zachodniopomorskie**:ad OR **krakow**:ad OR **cracow**:ad OR **wroclaw**:ad OR **poznan**:ad OR **gdansk**:ad OR **szczecin**:ad OR **bydgoszcz**:ad OR **katowice**:ad OR **bialystok**:ad OR **olsztyn**:ad OR **kielce**:ad OR **'zielona gora'**:ad OR **torun**:ad OR **'gorzow wielkopolski'**:ad OR **poland**:ab,ti OR **polska**:ab,ti OR **polish**:ab,ti OR **polski**:ab,ti OR **pole**:ab,ti OR **poles**:ab,ti OR **polak**:ab,ti OR **polka**:ab,ti OR **polacy**:ab,ti OR **warsaw**:ab,ti OR **warszawa**:ab,ti OR **wielkopolskie**:ab,ti OR **pomerania***:ab,ti OR **pomorskie**:ab,ti OR **kuyavian**:ab,ti OR **kujawsko**:ab,ti OR **malopolskie**:ab,ti OR **lodz**:ab,ti OR **lodzkie**:ab,ti OR **silesia***:ab,ti OR **dolnoslaskie**:ab,ti OR **lublin**:ab,ti OR **lubelskie**:ab,ti OR **lubus**:ab,ti OR **lubusz**:ab,ti OR **lubuskie**:ab,ti OR **masovia**:ab,ti OR **mazowske**:ab,ti OR **masovian**:ab,ti OR **mazowieckie**:ab,ti OR **opole**:ab,ti OR **opolskie**:ab,ti OR **podlaskie**:ab,ti OR **podlachia**:ab,ti OR **podlasie**:ab,ti OR **subcarpathian***:ab,ti OR **carpathian***:ab,ti OR **podkarpackie**:ab,ti OR **swietokrzyskie**:ab,ti OR **slaskie**:ab,ti OR **slask**:ab,ti OR **'varmia mazuria'**:ab,ti OR **'varmian mazurian'**:ab,ti OR **'varmia masuria'**:ab,ti OR **'varmian masurian'**:ab,ti OR **'warmia mazury'**:ab,ti OR **'warminsko mazurskie'**:ab,ti OR **zachodniopomorskie**:ab,ti OR **krakow**:ab,ti OR **cracow**:ab,ti OR **wroclaw**:ab,ti OR **poznan**:ab,ti OR **gdansk**:ab,ti OR **szczecin**:ab,ti OR **bydgoszcz**:ab,ti OR **katowice**:ab,ti OR **bialystok**:ab,ti OR **olsztyn**:ab,ti OR **kielce**:ab,ti OR **'zielona gora'**:ab,ti OR **torun**:ab,ti OR **'gorzow wielkopolski'**:ab,ti OR **netherlands**:ad OR **nederland***:ad OR **dutch***:ad OR **amsterdam**:ad OR **drenthe**:ad OR **flevoland**:ad OR **friesland**:ad OR **fryslan**:ad OR **gelderland**:ad OR **guelders**:ad OR **groningen**:ad OR **limburg**:ad OR **'north brabant'**:ad OR **'noord brabant'**:ad OR **holland**:ad OR **overijssel**:ad OR **overissel**:ad OR **utrecht**:ad OR **zeeland**:ad OR **rotterdam**:ad OR **hague**:ad OR **eindhoven**:ad OR **tilburg**:ad OR **almere**:ad OR **breda**:ad OR **nijmegen**:ad OR **nimeguen**:ad OR **netherlands**:ab,ti OR **nederland***:ab,ti OR **dutch***:ab,ti OR **amsterdam**:ab,ti OR **drenthe**:ab,ti OR **flevoland**:ab,ti OR **friesland**:ab,ti OR **fryslan**:ab,ti OR **gelderland**:ab,ti OR **guelders**:ab,ti OR **groningen**:ab,ti OR **limburg**:ab,ti OR **'north brabant'**:ab,ti OR **'noord brabant'**:ab,ti OR **holland**:ab,ti OR **overijssel**:ab,ti OR **overissel**:ab,ti OR **utrecht**:ab,ti OR **zeeland**:ab,ti OR **rotterdam**:ab,ti OR **hague**:ab,ti OR **eindhoven**:ab,ti OR **tilburg**:ab,ti OR **almere**:ab,ti OR **breda**:ab,ti OR **nijmegen**:ab,ti OR **nimeguen**:ab,ti OR **malta**:ab,ti OR **maltese**:ab,ti OR **valletta**:ab,ti OR **gozo**:ab,ti OR **ghawdex**:ab,ti OR **malta**:ad OR **maltese**:ad OR **valletta**:ad OR **gozo**:ad OR **ghawdex**:ad OR **luxembourg***:ab,ti OR **luxemburg**:ab,ti OR **letzebuerg**:ab,ti OR **diekirch**:ab,ti OR **grevenmacher**:ab,ti OR **luxembourg***:ad OR **luxemburg**:ad OR **letzebuerg**:ad OR **diekirch**:ad OR **grevenmacher**:ad OR **lithuania***:ab,ti OR **'lietuvos respublika'**:ab,ti OR **lietuva**:ab,ti OR **lietuviu**:ab,ti OR **vilnius**:ab,ti OR **vilniaus**:ab,ti OR **kaunas**:ab,ti OR **kauno**:ab,ti OR **klaipeda**:ab,ti OR **klaipedos**:ab,ti OR **panevezys**:ab,ti OR **panevezio**:ab,ti OR **siauliai**:ab,ti OR **siauliu**:ab,ti OR **alytus**:ab,ti OR **alytaus**:ab,ti OR **taurages**:ab,ti OR **taurage**:ab,ti OR **marijampoles**:ab,ti OR **marijampole**:ab,ti OR **telsiu**:ab,ti OR **telsiai**:ab,ti OR **utenos**:ab,ti OR **utena**:ab,ti OR **mazeikiai**:ab,ti OR **jonava**:ab,ti OR **mazeikiu**:ab,ti OR **jonavos**:ab,ti OR **lithuania***:ad OR **'lietuvos respublika'**:ad OR **lietuva**:ad OR **lietuviu**:ad OR **vilnius**:ad OR **vilniaus**:ad OR **kaunas**:ad OR **kauno**:ad OR **klaipeda**:ad OR **klaipedos**:ad OR **panevezys**:ad OR **panevezio**:ad OR **siauliai**:ad OR **siauliu**:ad OR **alytus**:ad OR **alytaus**:ad OR **taurages**:ad OR **taurage**:ad OR **marijampoles**:ad OR **marijampole**:ad OR **telsiu**:ad OR **telsiai**:ad OR **utenos**:ad OR **utena**:ad OR **mazeikiai**:ad OR **jonava**:ad OR **mazeikiu**:ad OR **jonavos**:ad OR **latvi***:ab,ti OR **latvija***:ab,ti OR **riga**:ab,ti OR **courland**:ab,ti OR **kurzeme**:ab,ti OR **kurland**:ab,ti OR **latgale**:ab,ti OR **lettgallia**:ab,ti OR **latgola**:ab,ti OR **vidzeme**:ab,ti OR **vidumo**:ab,ti OR **semigallia**:ab,ti OR **semigalia**:ab,ti OR **zemgale**:ab,ti OR **pieriga**:ab,ti OR **daugavpils**:ab,ti OR **dinaburg**:ab,ti OR **liepaja**:ab,ti OR **libau**:ab,ti OR **jelgava**:ab,ti OR **jurmala**:ab,ti OR **jekabpils**:ab,ti OR **jakobstadt**:ab,ti OR **rezekne**:ab,ti OR **rezne**:ab,ti OR **rositten**:ab,ti OR **valmiera**:ab,ti OR **wolmar**:ab,ti OR **ventspils**:ab,ti OR **windau**:ab,ti OR **latvi***:ad OR **latvija***:ad OR **riga**:ad OR **courland**:ad OR **kurzeme**:ad OR **kurland**:ad OR **latgale**:ad OR **lettgallia**:ad OR **latgola**:ad OR **vidzeme**:ad OR **vidumo**:ad OR **semigallia**:ad OR **semigalia**:ad OR **zemgale**:ad OR **pieriga**:ad OR **daugavpils**:ad OR **dinaburg**:ad OR **liepaja**:ad OR **libau**:ad OR **jelgava**:ad OR **jurmala**:ad OR **jekabpils**:ad OR **jakobstadt**:ad OR **rezekne**:ad OR **rezne**:ad OR **rositten**:ad OR **valmiera**:ad OR **wolmar**:ad OR **ventspils**:ad OR **windau**:ad \|  \| \| #1.4 \| **denmark**:ab,ti OR **danish***:ab,ti OR **danmark**:ab,ti OR **dansk***:ab,ti OR **hovedstaden**:ab,ti OR **midtjylland**:ab,ti OR **sjaelland**:ab,ti OR **sealand**:ab,ti OR **syddanmark**:ab,ti OR **jutland**:ab,ti OR **jylland**:ab,ti OR **nordjylland**:ab,ti OR **sonderjyllands**:ab,ti OR **'zealand region'**:ab,ti OR **'region zealand'**:ab,ti OR **hillerod**:ab,ti OR **viborg**:ab,ti OR **aalborg**:ab,ti OR **alborg**:ab,ti OR **soro**:ab,ti OR **vejle**:ab,ti OR **copenhagen**:ab,ti OR **kobenhavn**:ab,ti OR **arhus**:ab,ti OR **aarhus**:ab,ti OR **roskilde**:ab,ti OR **odense**:ab,ti OR **frederiksberg**:ab,ti OR **esbjerg**:ab,ti OR **gentofte**:ab,ti OR **gladsaxe**:ab,ti OR **randers**:ab,ti OR **kolding**:ab,ti OR **denmark**:ad OR **danish***:ad OR **danmark**:ad OR **dansk***:ad OR **hovedstaden**:ad OR **midtjylland**:ad OR **sjaelland**:ad OR **sealand**:ad OR **syddanmark**:ad OR **jutland**:ad OR **jylland**:ad OR **nordjylland**:ad OR **sonderjyllands**:ad OR **'zealand region'**:ad OR **'region zealand'**:ad OR **hillerod**:ad OR **viborg**:ad OR **aalborg**:ad OR **alborg**:ad OR **soro**:ad OR **vejle**:ad OR **copenhagen**:ad OR **kobenhavn**:ad OR **arhus**:ad OR **aarhus**:ad OR **roskilde**:ad OR **odense**:ad OR **frederiksberg**:ad OR **esbjerg**:ad OR **gentofte**:ad OR **gladsaxe**:ad OR **randers**:ad OR **kolding**:ad OR **czech***:ab,ti OR **cesk***:ab,ti OR **stredoces***:ab,ti OR **jihoces***:ab,ti OR **bohemia**:ab,ti OR **'bohemian region'**:ab,ti OR **kralovehradec***:ab,ti OR **'hradec kralove'**:ab,ti OR **karlovars***:ab,ti OR **'karlovy vary'**:ab,ti OR **liberec***:ab,ti OR **moravskoslezs***:ab,ti OR **'moravian silesian'**:ab,ti OR **olomouc***:ab,ti OR **pardubic***:ab,ti OR **plzen***:ab,ti OR **pilsen**:ab,ti OR **prage**:ab,ti OR **praha**:ab,ti OR **prag**:ab,ti OR **jihomorav***:ab,ti OR **moravia**:ab,ti OR **moravian**:ab,ti OR **morava**:ab,ti OR **usteck***:ab,ti OR **usti**:ab,ti OR **vysocina**:ab,ti OR **zlin**:ab,ti OR **zlinsk***:ab,ti OR **'ceske budejovice'**:ab,ti OR **budweis**:ab,ti OR **brno**:ab,ti OR **ostrava**:ab,ti OR **czech***:ad OR **cesk***:ad OR **stredoces***:ad OR **jihoce***:ad OR **bohemia**:ad OR **'bohemian region'**:ad OR **kralovehradec***:ad OR **'hradec kralove'**:ad OR **karlovars***:ad OR **'karlovy vary'**:ad OR **liberec***:ad OR **moravskoslezsk***:ad OR **'moravian silesian'**:ad OR **olomouc***:ad OR **pardubic***:ad OR **pardubice**:ad OR **plzen***:ad OR **pilsen**:ad OR **prage**:ad OR **praha**:ad OR **prag**:ad OR **jihomorav***:ad OR **moravia**:ad OR **moravian**:ad OR **morava**:ad OR **usteck***:ad OR **usti**:ad OR **vysocina**:ad OR **zlin**:ad OR **zlinsk***:ad OR **'ceske budejovice'**:ad OR **budweis**:ad OR **brno**:ad OR **ostrava**:ad OR **cyprus**:ab,ti OR **cypriot***:ab,ti OR **kypros**:ab,ti OR **kibris**:ab,ti OR **kypriaki***:ab,ti OR **nicosia**:ab,ti OR **lefkosa**:ab,ti OR **lefkosia**:ab,ti OR **famagusta**:ab,ti OR **magusa**:ab,ti OR **ammochostos**:ab,ti OR **gazimagusa**:ab,ti OR **kyrenia**:ab,ti OR **girne**:ab,ti OR **keryneia**:ab,ti OR **larnaca**:ab,ti OR **larnaka**:ab,ti OR **limassol**:ab,ti OR **lemesos**:ab,ti OR **limasol**:ab,ti OR **leymosun**:ab,ti OR **paphos**:ab,ti OR **pafos**:ab,ti OR **baf**:ab,ti OR **gazibaf**:ab,ti OR **protaras**:ab,ti OR **pergamos**:ab,ti OR **beyarmudu**:ab,ti OR **morfou**:ab,ti OR **guzelyurt**:ab,ti OR **omorfo**:ab,ti OR **morphou**:ab,ti OR **aradippou**:ab,ti OR **cyprus**:ad OR **cypriot***:ad OR **kypros**:ad OR **kibris**:ad OR **kypriaki***:ad OR **nicosia**:ad OR **lefkosa**:ad OR **lefkosia**:ad OR **famagusta**:ad OR **magusa**:ad OR **ammochostos**:ad OR **gazimagusa**:ad OR **kyrenia**:ad OR **girne**:ad OR **keryneia**:ad OR **larnaca**:ad OR **larnaka**:ad OR **limassol**:ad OR **lemesos**:ad OR **limasol**:ad OR **leymosun**:ad OR **paphos**:ad OR **pafos**:ad OR **baf**:ad OR **gazibaf**:ad OR **protaras**:ad OR **pergamos**:ad OR **beyarmudu**:ad OR **morfou**:ad OR **guzelyurt**:ad OR **omorfo**:ad OR **morphou**:ad OR **aradippou**:ad OR **croatia***:ab,ti OR **hrvatsk***:ab,ti OR **hrvat**:ab,ti OR **bjelovar**:ab,ti OR **'bjelovarsko bilogorska'**:ab,ti OR **'brod posavina'**:ab,ti OR **'brodsko posavska'**:ab,ti OR **'dubrovnik neretva'**:ab,ti OR **'dubrovacko neretvanska'**:ab,ti OR **istria**:ab,ti OR **istarska**:ab,ti OR **karlovacka**:ab,ti OR **karlovac**:ab,ti OR **'koprivnicko krizevacka'**:ab,ti OR **koprivnica**:ab,ti OR **krizevci**:ab,ti OR **'krapina zagorje'**:ab,ti OR **'krapinsko zagorska'**:ab,ti OR **'lika senj'**:ab,ti OR **'licko senjska'**:ab,ti OR **medimurska**:ab,ti OR **medimurje**:ab,ti OR **osijek**:ab,ti OR **osjecko**:ab,ti OR **baranja**:ab,ti OR **'osjecko baranjska'**:ab,ti OR **'pozega slavonia'**:ab,ti OR **'pozesko slavonska'**:ab,ti OR **'primorje gorski kotar'**:ab,ti OR **'primorsko goranska'**:ab,ti OR **'sibensko kninska'**:ab,ti OR **'sibensko kninske'**:ab,ti OR **sibenik**:ab,ti OR **knin**:ab,ti OR **sisak**:ab,ti OR **'sisacko moslavacka'**:ab,ti OR **moslavina**:ab,ti OR **'splitsko dalmatinska'**:ab,ti OR **split**:ab,ti OR **dalmatia**:ab,ti OR **varazdin**:ab,ti OR **varazdinska**:ab,ti OR **'viroviticko-podravska'**:ab,ti OR **virovitica**:ab,ti OR **podravina**:ab,ti OR **'vukovarsko srijemska'**:ab,ti OR **vukovar**:ab,ti OR **srijem**:ab,ti OR **zadar**:ab,ti OR **zadarska**:ab,ti OR **zagreb**:ab,ti OR **zagrebacka**:ab,ti OR **rijeka**:ab,ti OR **'velika gorica'**:ab,ti OR **'slavonski brod'**:ab,ti OR **pula**:ab,ti OR **croatia***:ad OR **hrvatsk***:ad OR **hrvat**:ad OR **bjelovar**:ad OR **'bjelovarsko bilogorska'**:ad OR **'brod posavina'**:ad OR **'brodsko posavska'**:ad OR **'dubrovnik neretva'**:ad OR **'dubrovacko neretvanska'**:ad OR **istria**:ad OR **istarska**:ad OR **karlovacka**:ad OR **karlovac**:ad OR **'koprivnicko krizevacka'**:ad OR **koprivnica**:ad OR **krizevci**:ad OR **'krapina zagorje'**:ad OR **'krapinsko zagorska'**:ad OR **'lika senj'**:ad OR **'licko senjska'**:ad OR **medimurska**:ad OR **medimurje**:ad OR **osijek**:ad OR **osjecko**:ad OR **baranja**:ad OR **'osjecko baranjska'**:ad OR **'pozega slavonia'**:ad OR **'pozesko slavonska'**:ad OR **'primorje gorski kotar'**:ad OR **'primorsko goranska'**:ad OR **'sibensko kninska'**:ad OR **'sibensko kninske'**:ad OR **sibenik**:ad OR **knin**:ad OR **sisak**:ad OR **'sisacko moslavacka'**:ad OR **moslavina**:ad OR **'splitsko dalmatinska'**:ad OR **split**:ad OR **dalmatia**:ad OR **varazdin**:ad OR **varazdinska**:ad OR **'viroviticko-podravska'**:ad OR **virovitica**:ad OR **podravina**:ad OR **'vukovarsko srijemska'**:ad OR **vukovar**:ad OR **srijem**:ad OR **zadar**:ad OR **zadarska**:ad OR **zagreb**:ad OR **zagrebacka**:ad OR **rijeka**:ad OR **'velika gorica'**:ad OR **'slavonski brod'**:ad OR **pula**:ad OR **bulgaria***:ab,ti OR **sofia**:ab,ti OR **gabrovo**:ab,ti OR **blagoevgrad**:ab,ti OR **'pirin macedonia'**:ab,ti OR **burgas**:ab,ti OR **dobrich**:ab,ti OR **haskovo**:ab,ti OR **kardzhali**:ab,ti OR **kurdzhali**:ab,ti OR **kyustendil**:ab,ti OR **lovech**:ab,ti OR **montana**:ab,ti OR **pazardzhik**:ab,ti OR **pernik**:ab,ti OR **pleven**:ab,ti OR **plovdiv**:ab,ti OR **razgrad**:ab,ti OR **rousse**:ab,ti OR **ruse**:ab,ti OR **shumen**:ab,ti OR **sliven**:ab,ti OR **silistra**:ab,ti OR **smolyan**:ab,ti OR **'stara zagora'**:ab,ti OR **targovishte**:ab,ti OR **varna**:ab,ti OR **tarnovo**:ab,ti OR **vidin**:ab,ti OR **vratsa**:ab,ti OR **vratza**:ab,ti OR **yambol**:ab,ti OR **bulgaria***:ad OR **sofia**:ad OR **gabrovo**:ad OR **blagoevgrad**:ad OR **'pirin macedonia'**:ad OR **burgas**:ad OR **dobrich**:ad OR **haskovo**:ad OR **kardzhali**:ad OR **kurdzhali**:ad OR **kyustendil**:ad OR **lovech**:ad OR **montana**:ad OR **pazardzhik**:ad OR **pernik**:ad OR **pleven**:ad OR **plovdiv**:ad OR **razgrad**:ad OR **rousse**:ad OR **ruse**:ad OR **shumen**:ad OR **sliven**:ad OR **silistra**:ad OR **smolyan**:ad OR **'stara zagora'**:ad OR **targovishte**:ad OR **varna**:ad OR **tarnovo**:ad OR **vidin**:ad OR **vratsa**:ad OR **vratza**:ad OR **yambol**:ad OR **belgi***:ab,ti OR **belge**:ab,ti OR **belgisch**:ab,ti OR **brussel***:ab,ti OR **bruxelles**:ab,ti OR **bruxelloise**:ab,ti OR **flemish**:ab,ti OR **flamand**:ab,ti OR **flemisch**:ab,ti OR **flanders**:ab,ti OR **flandern**:ab,ti OR **flandre**:ab,ti OR **vlaanderen**:ab,ti OR **vlaams**:ab,ti OR **flamande**:ab,ti OR **waals**:ab,ti OR **walloon***:ab,ti OR **wallon***:ab,ti OR **antwerp***:ab,ti OR **anvers**:ab,ti OR **ostflandern**:ab,ti OR **'vlaams brabant'**:ab,ti OR **limbourg**:ab,ti OR **limburg**:ab,ti OR **hainault**:ab,ti OR **hainaut**:ab,ti OR **henegouwen**:ab,ti OR **hennegau**:ab,ti OR **liege**:ab,ti OR **luik**:ab,ti OR **luttich**:ab,ti OR **namur**:ab,ti OR **namen**:ab,ti OR **westflandern**:ab,ti OR **'waals brabant'**:ab,ti OR **ghent**:ab,ti OR **gent**:ab,ti OR **gand**:ab,ti OR **charleroi**:ab,ti OR **bruges**:ab,ti OR **brugge**:ab,ti OR **schaerbeek**:ab,ti OR **schaarbeek**:ab,ti OR **anderlecht**:ab,ti OR **leuven**:ab,ti OR **louvain**:ab,ti OR **hasselt**:ab,ti OR **mons**:ab,ti OR **wavre**:ab,ti OR **waver**:ab,ti OR **belgi***:ad OR **belge**:ad OR **belgisch**:ad OR **brussel***:ad OR **bruxelles**:ad OR **bruxelloise**:ad OR **flemish**:ad OR **flamand**:ad OR **flemisch**:ad OR **flanders**:ad OR **flandern**:ad OR **flandre**:ad OR **vlaanderen**:ad OR **vlaams**:ad OR **flamande**:ad OR **waals**:ad OR **walloon***:ad OR **wallon***:ad OR **antwerp***:ad OR **anvers**:ad OR **ostflandern**:ad OR **'vlaams brabant'**:ad OR **limbourg**:ad OR **limburg**:ad OR **hainault**:ad OR **hainaut**:ad OR **henegouwen**:ad OR **hennegau**:ad OR **liege**:ad OR **luik**:ad OR **luttich**:ad OR **namur**:ad OR **namen**:ad OR **westflandern**:ad OR **'waals brabant'**:ad OR **ghent**:ad OR **gent**:ad OR **gand**:ad OR **charleroi**:ad OR **bruges**:ad OR **brugge**:ad OR **schaerbeek**:ad OR **schaarbeek**:ad OR **anderlecht**:ad OR **leuven**:ad OR **louvain**:ad OR **hasselt**:ad OR **mons**:ad OR **wavre**:ad OR **waver**:ad OR **austria***:ab,ti OR **vienna**:ab,ti OR **wien**:ab,ti OR **osterreich***:ab,ti OR **sudosterreich**:ab,ti OR **westosterreich**:ab,ti OR **niederosterreich**:ab,ti OR **burgenland**:ab,ti OR **carinthia**:ab,ti OR **karinthia**:ab,ti OR **karnten**:ab,ti OR **oberosterreich**:ab,ti OR **styria**:ab,ti OR **steiermark**:ab,ti OR **salzburg**:ab,ti OR **saizburg**:ab,ti OR **tyrol**:ab,ti OR **tirol**:ab,ti OR **becs**:ab,ti OR **vorarlberg**:ab,ti OR **bregenz**:ab,ti OR **linz**:ab,ti OR **eisenstadt**:ab,ti OR **innsbruck**:ab,ti OR **graz**:ab,ti OR **klagenfurt**:ab,ti OR **polten**:ab,ti OR **villach**:ab,ti OR **wels**:ab,ti OR **dornbirn**:ab,ti OR **feldkirch**:ab,ti OR **steyr**:ab,ti OR **austria***:ad OR **vienna**:ad OR **wien**:ad OR **osterreich***:ad OR **sudosterreich**:ad OR **westosterreich**:ad OR **niederosterreich**:ad OR **burgenland**:ad OR **carinthia**:ad OR **karnten**:ad OR **oberosterreich**:ad OR **styria**:ad OR **steiermark**:ad OR **salzburg**:ad OR **saizburg**:ad OR **tyrol**:ad OR **tirol**:ad OR **becs**:ad OR **vorarlberg**:ad OR **bregenz**:ad OR **linz**:ad OR **eisenstadt**:ad OR **innsbruck**:ad OR **graz**:ad OR **klagenfurt**:ad OR **polten**:ad OR **villach**:ad OR **wels**:ad OR **dornbirn**:ad OR **feldkirch**:ad OR **steyr**:ad \|  \| \| #1.3 \| **iceland**:ab,ti OR **icelandic***:ab,ti OR **islenska***:ab,ti OR **icelander***:ab,ti OR **islendinga***:ab,ti OR **reykjavik**:ab,ti OR **reykjavikurborg**:ab,ti OR **hofudborgarsvaedid**:ab,ti OR **sudurnes**:ab,ti OR **vesturland**:ab,ti OR **vestfirdir**:ab,ti OR **westfjords**:ab,ti OR **nordurland**:ab,ti OR **austurland**:ab,ti OR **sudurland**:ab,ti OR **kopavogur**:ab,ti OR **hafnarfjordur**:ab,ti OR **iceland**:ad OR **icelandic***:ad OR **islenska***:ad OR **icelander***:ad OR **islendinga***:ad OR **reykjavik**:ad OR **reykjavikurborg**:ad OR **hofudborgarsvaedid**:ad OR **sudurnes**:ad OR **vesturland**:ad OR **vestfirdir**:ad OR **westfjords**:ad OR **nordurland**:ad OR **austurland**:ad OR **sudurland**:ad OR **kopavogur**:ad OR **hafnarfjordur**:ad OR **switzerland**:ab,ti OR **schweiz**:ab,ti OR **schweizerische**:ab,ti OR **swiss**:ab,ti OR **suisse**:ab,ti OR **aargau**:ab,ti OR **argovia**:ab,ti OR **ausserrhoden**:ab,ti OR **'outer rhodes'**:ab,ti OR **innerrhoden**:ab,ti OR **'inner rhodes'**:ab,ti OR **basel**:ab,ti OR **bern**:ab,ti OR **berne**:ab,ti OR **fribourg**:ab,ti OR **freiburg**:ab,ti OR **geneva**:ab,ti OR **geneve**:ab,ti OR **glarus**:ab,ti OR **graubunden**:ab,ti OR **grisons**:ab,ti OR **grigioni**:ab,ti OR **jura**:ab,ti OR **lucerne**:ab,ti OR **luzern**:ab,ti OR **neuchatel**:ab,ti OR **zurich**:ab,ti OR (**uri**:ab,ti AND (**canton**:ab,ti OR **kanton**:ab,ti)) OR **schwyz**:ab,ti OR **obwalden**:ab,ti OR **nidwalden**:ab,ti OR **zug**:ab,ti OR **solothurn**:ab,ti OR **schaffhausen**:ab,ti OR **thurgau**:ab,ti OR **thurgovia**:ab,ti OR **ticino**:ab,ti OR **tessin**:ab,ti OR **vaud**:ab,ti OR **valais**:ab,ti OR **wallis**:ab,ti OR **'st gallen'**:ab,ti OR **lausanne**:ab,ti OR **winterthur**:ab,ti OR **winterthour**:ab,ti OR **lugano**:ab,ti OR **biel**:ab,ti OR **bienne**:ab,ti OR **switzerland**:ad OR **schweiz**:ad OR **schweizerische**:ad OR **swiss**:ad OR **suisse**:ad OR **aargau**:ad OR **argovia**:ad OR **ausserrhoden**:ad OR **'outer rhodes'**:ad OR **innerrhoden**:ad OR **'inner rhodes'**:ad OR **basel**:ad OR **bern**:ad OR **berne**:ad OR **fribourg**:ad OR **freiburg**:ad OR **geneva**:ad OR **geneve**:ad OR **glarus**:ad OR **graubunden**:ad OR **grisons**:ad OR **grigioni**:ad OR **jura**:ad OR **lucerne**:ad OR **luzern**:ad OR **neuchatel**:ad OR **zurich**:ad OR (**uri**:ad AND (**canton**:ad OR **kanton**:ad)) OR **schwyz**:ad OR **obwalden**:ad OR **nidwalden**:ad OR **zug**:ad OR **solothurn**:ad OR **schaffhausen**:ad OR **thurgau**:ad OR **thurgovia**:ad OR **ticino**:ad OR **tessin**:ad OR **vaud**:ad OR **valais**:ad OR **wallis**:ad OR **'st gallen'**:ad OR **lausanne**:ad OR **winterthur**:ad OR **winterthour**:ad OR **lugano**:ad OR **biel**:ad OR **bienne**:ad OR **norway**:ab,ti OR **norwegian***:ab,ti OR **norge**:ab,ti OR **noreg**:ab,ti OR **norgga**:ab,ti OR **ostfold**:ab,ti OR **akershus**:ab,ti OR **oslo**:ab,ti OR **hedmark**:ab,ti OR **oppland**:ab,ti OR **buskerud**:ab,ti OR **vestfold**:ab,ti OR **telemark**:ab,ti OR **'aust agder'**:ab,ti OR **'vest agder'**:ab,ti OR **rogaland**:ab,ti OR **hordaland**:ab,ti OR **'sogn og fjordane'**:ab,ti OR **'sogn and fjordane'**:ab,ti OR **'sogn fjordane'**:ab,ti OR **'more og romsdal'**:ab,ti OR **'more and romsdal'**:ab,ti OR **'more romsdal'**:ab,ti OR **trondelag**:ab,ti OR **nordland**:ab,ti OR **troms**:ab,ti OR **finnmark**:ab,ti OR **bergen**:ab,ti OR **stavanger**:ab,ti OR **sandnes**:ab,ti OR **trondheim**:ab,ti OR **kristiansand**:ab,ti OR **drammen**:ab,ti OR **fredrikstad**:ab,ti OR **sarpsborg**:ab,ti OR **porsgrunn**:ab,ti OR **skien**:ab,ti OR **tonsberg**:ab,ti OR **alesund**:ab,ti OR **norway**:ad OR **norwegian***:ad OR **norge**:ad OR **noreg**:ad OR **norgga**:ad OR **ostfold**:ad OR **akershus**:ad OR **oslo**:ad OR **hedmark**:ad OR **oppland**:ad OR **buskerud**:ad OR **vestfold**:ad OR **telemark**:ad OR **'aust agder'**:ad OR **'vest agder'**:ad OR **rogaland**:ad OR **hordaland**:ad OR **'sogn og fjordane'**:ad OR **'sogn and fjordane'**:ad OR **'sogn fjordane'**:ad OR **'more og romsdal'**:ad OR **'more and romsdal'**:ad OR **'more romsdal'**:ad OR **trondelag**:ad OR **nordland**:ad OR **troms**:ad OR **finnmark**:ad OR **bergen**:ad OR **stavanger**:ad OR **sandnes**:ad OR **trondheim**:ad OR **kristiansand**:ad OR **drammen**:ad OR **fredrikstad**:ad OR **sarpsborg**:ad OR **porsgrunn**:ad OR **skien**:ad OR **tonsberg**:ad OR **alesund**:ad OR **liechtenstein**:ab,ti OR **vaduz**:ab,ti OR **triesenberg**:ab,ti OR **triesen**:ab,ti OR **schellenberg**:ab,ti OR **schaan**:ab,ti OR **ruggell**:ab,ti OR **planken**:ab,ti OR **mauren**:ab,ti OR **gamprin**:ab,ti OR **eschen**:ab,ti OR **balzers**:ab,ti OR **liechtenstein**:ad OR **vaduz**:ad OR **triesenberg**:ad OR **triesen**:ad OR **schellenberg**:ad OR **schaan**:ad OR **ruggell**:ad OR **planken**:ad OR **mauren**:ad OR **gamprin**:ad OR **eschen**:ad OR **balzers**:ad \|  \| \| #1.2 \| **'turkey (republic)'**/exp OR **turkey**:ab,ti OR **turkiye**:ab,ti OR **turkish**:ab,ti OR **istanbul**:ab,ti OR **marmara**:ab,ti OR **aegean**:ab,ti OR **anatolia**:ab,ti OR **'black sea'**:ab,ti OR **tekirdag**:ab,ti OR **balikesir**:ab,ti OR **izmir**:ab,ti OR **aydin**:ab,ti OR **manisa**:ab,ti OR **bursa**:ab,ti OR **kocaeli**:ab,ti OR **ankara**:ab,ti OR **konya**:ab,ti OR **antalya**:ab,ti OR **adana**:ab,ti OR **hatay**:ab,ti OR **kirikkale**:ab,ti OR **kayseri**:ab,ti OR **zonguldak**:ab,ti OR **kastamonu**:ab,ti OR **samsun**:ab,ti OR **trabzon**:ab,ti OR **erzurum**:ab,ti OR **agri**:ab,ti OR **malatya**:ab,ti OR (**van**:ab,ti AND (**region**:ab,ti OR **subregion**:ab,ti OR **bolgesi**:ab,ti)) OR **gaziantep**:ab,ti OR **sanliurfa**:ab,ti OR **mardin**:ab,ti OR **mersin**:ab,ti OR **turkey**:ad OR **turkiye**:ad OR **turkish**:ad OR **istanbul**:ad OR **marmara**:ad OR **aegean**:ad OR **anatolia**:ad OR **'black sea'**:ad OR **tekirdag**:ad OR **balikesir**:ad OR **izmir**:ad OR **aydin**:ad OR **manisa**:ad OR **bursa**:ad OR **kocaeli**:ad OR **ankara**:ad OR **konya**:ad OR **antalya**:ad OR **adana**:ad OR **hatay**:ad OR **kirikkale**:ad OR **kayseri**:ad OR **zonguldak**:ad OR **kastamonu**:ad OR **samsun**:ad OR **trabzon**:ad OR **erzurum**:ad OR **agri**:ad OR **malatya**:ad OR (**van**:ad AND (**region**:ad OR **subregion**:ad OR **bolgesi**:ad)) OR **gaziantep**:ad OR **sanliurfa**:ad OR **mardin**:ad OR **mersin**:ad \| **292777** \| \| #1.1 \| **'europe'**/exp OR **'european union'**/exp OR **europa**:ab,ti OR **europe***:ab,ti OR **scandinavia***:ab,ti OR **scandinavia***:ad OR **mediterranean**:ab,ti OR **'eea countries'**:ab,ti OR **mediterranean**:ad OR **europe***:ad OR **baltic**:ab,ti OR **baltic**:ad OR **yugoslavia**:ab,ti OR **jugoslavija**:ab,ti OR **jugoslavija**:ad OR **yugoslavia**:ad OR **'eu country'**:ab,ti OR **'eu countries'**:ab,ti OR **global***:ab,ti OR **world**:ab,ti OR **worldwide**:ab,ti \| **2166** \| | |

## Cochrane Library (CDSR, DARE, HTA, EED)

Date of the search: 12/03/2015

Language limits: no limits

Date limits: 2005-2015

Number of results: CDSR: 3; DARE: 7; HTA: 2; EED: 25

ID Search Hits

#1 MeSH descriptor: [Prevalence] explode all trees 3943

#2 MeSH descriptor: [Population Surveillance] explode all trees 585

#3 MeSH descriptor: [Seroepidemiologic Studies] explode all trees 134

#4 prevalence* or seroepidemiolog* or "sero epidemiologic" or "sero epidemiological" 15921
or "sero epidemiology" or serosurvey* or serolog* or epidemiolog* or
surveillance:ti,ab,kw

#5 #1 or #2 or #3 or #4 19532

#6 MeSH descriptor: [Hepatitis B] explode all trees 1939

#7 MeSH descriptor: [Hepatitis B Antibodies] explode all trees 555

#8 MeSH descriptor: [Hepatitis B Antigens] explode all trees 940

#9 MeSH descriptor: [Hepatitis B virus] explode all trees 671

#10 MeSH descriptor: [Hepatitis C] explode all trees 2287

#11 MeSH descriptor: [Hepatitis C Antigens] explode all trees 15

#12 MeSH descriptor: [Hepatitis C Antibodies] explode all trees 112

#13 MeSH descriptor: [Hepacivirus] explode all trees 1001

#14 "hepatitis b" or "hepatitis c" or hepaciviru* or "hbv" or "hcv" or "hbsag" or "hbs ag"

6994
or "Australia Antigen" or "Australia Antigens":ti,ab,kw Publication

#15 #6 or #7 or #8 or #9 or #10 or #11 or #12 or #13 or #14

8380

#16 #15 and #5 517

# Annex 2: List and definition of key subgroups included in the systematic review

| **Population category** | **Definition** |
| --- | --- |
| **Prisoners** | Prison inmates and people incarcerated in custodial or prison settings including youth detention centres, excluding formerly incarcerated populations and people in other non-custodial closed/fixed institutions (such as secure psychiatric hospitals). Psychiatric prison hospitals inmates (i.e. people with severe mental illness that are serving custodial sentences) are included |
| **MSM** | Men who have sex with men |

# Annex 3: Study inclusion and exclusion criteria

| **Inclusion criteria** | **Exclusion criteria** |
| --- | --- |
| Articles published in 2005 or later reporting data from population sampled from the year 2000 onwards, including studies with data collection ending after 2000 (irrespective of start date) | Articles falling outside the specified sampling period or publication date range |
| Articles reporting data from one or more EU/EEA MS and/or any of their regions/districts | Articles reporting data on non EU/EEA countries only |
| Articles reporting HBsAg/anti-HCV (and DNA/RNA) prevalence rates in humans | Articles not reporting data on HBsAg/anti-HCV prevalence or DNA/RNA or if virological markers tested for were not specified  Article reporting only self-reported (i.e. unconfirmed) HBsAg/anti-HCV prevalence |
| Articles reporting data from prisoners and MSM (Table 1) | Articles reporting data on specific high-risk groups only (Table 3) |
|  | Articles reporting modelled data only |
|  | Articles reporting only data from a study not conducted in humans, environmental studies, technology assessments (studies on diagnostic and/or laboratory methods) |
|  | Opinion papers, editorials, guidelines or recommendations , perspectives and correspondence articles, systematic reviews or meta-analysis |

# Annex 4: PRISMA Checklist

| **Section/topic** | **#** | **Checklist item** | **Reported on page #** |
| --- | --- | --- | --- |
| **TITLE** | | |  |
| Title | 1 | Identify the report as a systematic review, meta-analysis, or both. | 1 |
| **ABSTRACT** | | |  |
| Structured summary | 2 | Provide a structured summary including, as applicable: background; objectives; data sources; study eligibility criteria, participants, and interventions; study appraisal and synthesis methods; results; limitations; conclusions and implications of key findings; systematic review registration number. | 2 |
| **INTRODUCTION** | | |  |
| Rationale | 3 | Describe the rationale for the review in the context of what is already known. | 5 |
| Objectives | 4 | Provide an explicit statement of questions being addressed with reference to participants, interventions, comparisons, outcomes, and study design (PICOS). | 5 |
| **METHODS** | | |  |
| Protocol and registration | 5 | Indicate if a review protocol exists, if and where it can be accessed (e.g., Web address), and, if available, provide registration information including registration number. | N/A |
| Eligibility criteria | 6 | Specify study characteristics (e.g., PICOS, length of follow-up) and report characteristics (e.g., years considered, language, publication status) used as criteria for eligibility, giving rationale. | 6 – 7 |
| Information sources | 7 | Describe all information sources (e.g., databases with dates of coverage, contact with study authors to identify additional studies) in the search and date last searched. | 6 – 7 |
| Search | 8 | Present full electronic search strategy for at least one database, including any limits used, such that it could be repeated. | Supplement file |
| Study selection | 9 | State the process for selecting studies (i.e., screening, eligibility, included in systematic review, and, if applicable, included in the meta-analysis). | 6 - 7 |
| Data collection process | 10 | Describe method of data extraction from reports (e.g., piloted forms, independently, in duplicate) and any processes for obtaining and confirming data from investigators. | 7 |
| Data items | 11 | List and define all variables for which data were sought (e.g., PICOS, funding sources) and any assumptions and simplifications made. | Available from the author on request |
| Risk of bias in individual studies | 12 | Describe methods used for assessing risk of bias of individual studies (including specification of whether this was done at the study or outcome level), and how this information is to be used in any data synthesis. | 8 and supplement |
| Summary measures | 13 | State the principal summary measures (e.g., risk ratio, difference in means). | 6 |
| Synthesis of results | 14 | Describe the methods of handling data and combining results of studies, if done, including measures of consistency (e.g., I^2^) for each meta-analysis. | 8 |

| **Reasons for Exclusion** | Prisoners | MSM |
| --- | --- | --- |
| Notification or incidence data/no prevalence data | 4 | 3 |
| Irrelevant/unspecified virological markers | 1 | 2 |
| Self-reported serological status/unclear confirmation of serological status | 3 | 1 |
| Duplicate reference/data | 6 | 1 |
| No original data (commentary, erratum, guidelines etc.) | 4 | 0 |
| Conference abstract but full paper has been published | 1 | 0 |
| Review article | 2 | 0 |
| Population bias (e.g. ex-prisoners/homeless population) | 6 | 0 |
| Outside date range | 0 | 0 |
| Modelled data only | 2 | 0 |
| No full text available | 3 | 0 |
| Total | 32 | 7 |

# Annex 5: Reasons for exclusion of full text for studies retrieved for MSM and prisoners

# Annex 6: Risk of bias framework for studies among MSM

| **Domain** | **Scores and Description** |
| --- | --- |
| Sampling venue coverage | 0 – Single venue;  1 – Multi-centre/single venue type;  2 – Multi-centre/multi-venue type |

# Annex 7. Risk of bias framework for studies among prisoners

| **Domain** | **Scores and description** | |
| --- | --- | --- |
| Age | 0 – Clear age bias (i.e. among juvenile offenders only); no information | 1 – No clear bias in age profile of subjects |
| Gender | 0 – Clear bias in gender or no information on male/female ratio | 1 – No clear bias in gender distribution of subjects; could be considered representative if information is limited |
| Proportion PWID | 0 – Exclusively among PWID/former PWID prisoners | 1 – Injecting drug use (current/former) not used to select subjects; no bias toward PWID only |
| Sampling method | 0 – Non-random or non-exhaustive | 1 – Exhaustive or random |
| Population coverage | 0 – Single centre/local;  1 – Multi-centre/local or regional;  2 – Multi-centre/national | |

# Annex 8: Study characteristics for HBsAg prevalence estimates in MSM

| Country | Author | Sampling period | Risk of bias score | N | Sampling method | Sampling description | Age range | Prevalence | Lower 95%CI | Upper 95%CI |
| --- | --- | --- | --- | --- | --- | --- | --- | --- | --- | --- |
| Croatia | Bozicevic et al (2009) | 2006 | 2 | 360 | Respondent-driven | Zagreb | N/R | 0.6% | 0.1% | 2.0% |
| Estonia | Rüütel et al (2015b) | 2014-2015 | 1 | 97 | Respondent-driven | Online survey with free, anonymous STI screening | 14 to 68. Mean age 30 | 1.0% | 0.0% | 5.6% |
| Estonia | Rüütel et al (2015a) | 2013 | 1 | 43 | Convenience | Online survey with free, anonymous STI screening | 18 to 67. Mean age 33 | 0.0% | 0.0% | 8.2% |
| France | Sauvage et al (2015) | 2009 | 2 | 876 | Convenience | Screening offered in 14 bars, saunas and ' backrooms' | >18 | 1.4% | 0.7% | 2.4% |
| The UK | McMillan (2006) | 2001-2003 | 0 | 575 | Exhaustive | Retrospective analysis of samples from all new clients of Edinburgh STI clinic | 15 to 64. Mean age 29 | 1.0% | 0.4% | 2.3% |
| The UK | Roy et al (2008) | 2001 | 1 | 81 | Convenience | STI clinic samples | N/R | 0.0% | 0.0% | 4.5% |

# Annex 9: Study characteristics for Anti-HCV prevalence estimates in MSM

| Country | Author (Publ.Year) | Sampling period | Risk of bias score | N | Sampling method | Sampling description | Age range | Anti-HCV Prevalence | Lower 95%CI | Upper 95%CI |
| --- | --- | --- | --- | --- | --- | --- | --- | --- | --- | --- |
| Croatia | Cavlek et al (2009) | 2003-2006 | 2 | 205 | N/R | Survey in seven cities | N/R | 2.9% | 1.1% | 6.3% |
| Croatia | Bozicevic et al (2009) | 2006 | 2 | 360 | Respondent-driven | Zagreb | N/R | 2.5% | 1.1% | 4.7% |
| Estonia | Rüütel et al (2015a) | 2013 | 1 | 43 | Convenience | Online survey with free, anonymous STI screening | 18 to 67. Mean age 33 | 4.7% | 0.6% | 15.8% |
| Estonia | Rüütel et al (2015b) | 2014-2015 | 1 | 113 | Respondent-driven | Online survey with free, anonymous STI screening | 14 to 68. Mean age 30 | 1.8% | 0.2% | 6.2% |
| France | Sauvage et al (2015) | 2009 | 2 | 876 | Convenience | Screening offered in 14 bars, saunas and ' backrooms' | >18 | 1.0% | 0.5% | 1.9% |
| Italy | Di Benedetto et al (2012) | 2010 | 2 | 74 | Convenience | Men living in Sicily for more than 6 months recruited via internet and in gay bars | 18 to 56. Mean age 30 | 0.0% | 0.0% | 4.9% |
| Sweden | Blaxhult et al (2013) | 2012-2013 | 1 | 1008 | Convenience | Attendees of a Stockholm STI clinic | 16 to 82. Mean age 33 | 0.6% | 0.2% | 1.3% |
| The Netherlands | van de Laar et al (2007) | 1984-2003 | 2 | 1836 | Convenience | Cohort study in Amsterdam | Mean age 31.8 | 1.3% | 0.8% | 1.9% |
| The Netherlands | Van Rooijen et al (2013) | 2007 | 1 | 450 | Convenience | STI clinic attendees opting out of HIV testing | N/R | 0.7% | 0.1% | 1.9% |
| The UK | Price et al (2013) | 2008 | 2 | 1121 | Convenience | Multi-centre study in gay bars, saunas and clubs | 16 to 51 | 2.1% | 1.4% | 3.2% |
| The UK | Donson et al (2012) | 2009-2011 | 0 | 3395 | Convenience | STI clinic attendees | N/R | 1.6% | 1.2% | 2.0% |

# Annex 10: Study characteristics for HBsAg prevalence estimates in people in prison

| Country | Author (Publ.Year) | Sampling period | Risk of bias score | N | Sampling method | Sampling description | Age range | HBsAg Prevalence | Lower 95%CI | Upper 95%CI |
| --- | --- | --- | --- | --- | --- | --- | --- | --- | --- | --- |
| Bulgaria | Popov et al (2012) | 2010 | 3 | 258 | Convenience | Study in two juvenile facilities | N/R (juvenile) | 25.2% | 20.0% | 31.0% |
| ***Croatia*** | ***Pooled (Burekt et al 2009; Burek et al 2010)*** | ***2004-2007*** | ***N/A*** | ***6508*** | ***Pooled*** | ***Pooled*** | ***Adults*** | ***1.3%*** | ***1.0%*** | ***1.6%*** |
| Croatia | Burek et al (2010) | 2005-2007 | 4 | 140 | Convenience | Multi-centre study in 20 prisons. Sample includes PWID | 16 to 18 | 1.4% | 0.2% | 5.1% |
| Finland | Viitanen et al (2011) | 2006 | 6 | 383 | Mixed methods | National coverage | 16 to 69 | 0.5% | 0.1% | 1.9% |
| France | Abergel et al (2014) | 2012-2013 | 5 | 347 | Exhaustive | Two *maisons d'arret* in Clermont-Ferrand and Riom. 97% male | 25 to 39. Mean age 30 | 0.6% | 0.1% | 2.1% |
| Hungary | Treso et al (2012) | 2007-2009 | 6 | 4894 | Exhaustive | All inmates | 21 to 60 | 1.5% | 1.2% | 1.8% |
| Ireland | Drummond et al (2014) | 2011 | 6 | 777 | Random (49.5% response) | National coverage - all adult inmates (sentenced and remand) | Mean age 31 | 0.3% | 0.0% | 0.9% |
| Italy | Babudieri et al (2005) | 2001-2002 | 4 | 973 | Convenience | Multi-centre study in eight prisons. Mixed gender. Includes PWID | Mean age 36 | 6.7% | 5.2% | 8.4% |
| Luxembourg | Removille et al (2011) | 2005 | 4 | 115 | Convenience | Multi-centre study in the two prisons. Population of problem drug users (not all PWID) | N/R | 7.0% | 3.1% | 13.2% |
| Portugal | Marques et al (2011) | 2007-2008 | 4 | 151 | Exhaustive (71.6% response) | Study in regional prison of Coimbra. Includes PWID | 19 to 75. Mean age 34.1 | 0.7% | 0.0% | 3.6% |
| Romania | Nazare (2011) | 2007-2010 | 1 | 197 | Convenience | Single prison screening study | N/R | 10.7% | 6.7% | 15.8% |
| Spain | Garcia-Guerrero et al (2010) | 2008 | 6 | N/R | Random (92% response) | Multi-centre study in 18 prisons across Spain | Mean age 35.7 | 2.6% | 0.8% | 4.5% |
| ***The UK*** | ***Pooled*** | ***2010-2013*** | ***N/A*** | ***640*** | ***Pooled*** | ***Pooled*** | ***Pooled*** | ***1.6%*** | ***0.8%*** | ***2.9%*** |

# Annex 11: Study characteristics for anti-HCV prevalence estimates in people in prison

| **Country** | **Author (Publ.Year)** | **Sampling period** | **Risk of bias score** | **N** | **Sampling method** | **Sampling description** | **Age range** | **Prevalence** | **Lower 95%CI** | **Upper 95%CI** |
| --- | --- | --- | --- | --- | --- | --- | --- | --- | --- | --- |
| Bulgaria | Pooled estimates | N/A | N/A | 1156 | Pooled | Pooled | Pooled | 26.31% | 23.48% | 29.29% |
| Bulgaria | Popov et al (2012) | 2010 | 3 | 258 | Convenience | Study in two juvenile detention facilities | N/R (juveniles) | 20.54% | 15.78% | 25.99% |
| Croatia | Pooled | 2004-2007 | N/A | 6696 | Pooled | Pooled (Burek, 2009 and Burek, 2010) | Pooled | 13.34% | 12.53% | 14.17% |
| Croatia | Burek et al (2010) | 2005-2007 | 4 | 140 | Convenience | Multi-centre study in 20 prisons. Sample includes PWID. Juvenile | 16 to 18 | 4.29% | 1.59% | 9.09% |
| Finland | Viitanen et al (2011) | 2006 | 6 | 383 | Mixed methods | National coverage | 16 to 69 | 45.83% | 40.77% | 50.96% |
| France | Pooled | 2000-2013 | N/A | 68797 | Pooled | Pooled | Pooled | 6.28% | 6.10% | 6.46% |
| Germany | Meyer (2007) | 2002 | 3 | 1125 | Exhaustive (96% response) | Largest German juvenile prison. All new inmates offered screening | >16 | 8.62% | 7.05% | 10.42% |
| Hungary | Treso et al (2012) | 2007-2009 | 6 | 4894 | Exhaustive | All inmates | 21 to 60 | 4.92% | 4.34% | 5.57% |
| Ireland | Drummond et al (2014) | 2011 | 6 | 777 | Random (49.5% response) | National coverage - all adult inmates (sentenced and remand) | Mean age 31 | 12.87% | 10.60% | 15.43% |
| Italy | Babudieri et al (2005) | 2001-2002 | 4 | 973 | Convenience | Multi-centre study in eight prisons. Mixed gender. Includes PWID | Mean age 36 | 38.03% | 34.97% | 41.16% |
| Luxembourg | Removille et al (2011) | 2005 | 4 | 122 | Convenience | Multi-centre study in the two prisons. Population of problem drug users (not all PWID) | N/R | 86.29% | 78.96% | 91.81% |
| Portugal | Marques et al (2011) | 2007-2008 | 4 | 151 | Exhaustive (71.6% response) | Study in regional prison of Coimbra. Includes PWID | 19 to 75.  Mean age 34.1 | 34.44% | 26.90% | 42.60% |
| Spain | Hernandez-Fernandez (2010) | 2009 | 5 | N/R | Other | Data from the National Centre for Prison Health Co-ordination | N/R | 25.30% |  |  |
| Spain | Garcia-Guerrero et al (2010) | 2008 | 6 | N/R | Random (92% response) | Multi-centre study in 18 prisons across Spain | Mean age 35.7 | 22.70% | 18.30% | 27.10% |
| Spain | Pooled | 2000-2009 | N/A | 3062 | Pooled | Pooled | Pooled | 20.28% | 18.87% | 21.75% |
| The UK | Pooled | 2010-2013 | N/A | 5450 | Pooled | Pooled | Pooled | 17.39% | 16.40% | 18.43% |
